# Supplementary material for: Experiences, barriers, and facilitators of health data use among performance monitoring teams (PMT) of health facilities in Eastern Ethiopia: A qualitative study
Source: PLoS One. 2023 May 11;18(5):e0285662. doi: 10.1371/journal.pone.0285662 (PMC10174501; doi:10.1371/journal.pone.0285662)
Supplement: Attachment — Submitted filename: PONE-D-22-10622 (1)_SC.pdf [file pone.0285662.s001.pdf]

PLOS ONE

Experience, Challenges, and Facilitators of Health Data Use among Performance Monitoring Teams (PMT) of Health Facilities in Eastern Ethiopia: A Qualitative Study.

--Manuscript Draft--

**Manuscript Number:**	PONE-D-22-10622
**Article Type:**	Research Article
**Full Title:**	Experience, Challenges, and Facilitators of Health Data Use among Performance Monitoring Teams (PMT) of Health Facilities in Eastern Ethiopia: A Qualitative Study.
**Short Title:**	Barriers and facilitators of health data use by Performance Monitoring Teams
**Corresponding Author:**	Admas Abera, MSc in Epidemiology<br>Haramaya University College of Health and Medical Sciences<br>HARAR, ETHIOPIA
**Keywords:**	Key words: Data use, Performance monitoring teams, challenge and facilitators, healthcare facilities, Ethiopia.
**Abstract:**	Abstract<br>Background: Using routine health data plays a crucial role in decisions towards improved health outcomes. Despite significant investments in improving Ethiopia's Performance Monitoring Teams (PMT), there is limited evidence on the facilitators and barriers to data utilization by this team in responding to present and developing health challenges. This study, therefore, is aimed at exploring PMT experiences, facilitators and barriers to information use in healthcare facilities in eastern Ethiopia.<br>Method: This study employed a phenomenological study design . Consolidated Framework for Implementation Research (CFIR) framework was adapted to identify the most relevant constructs with the aim of describing data use approaches. Key informant interviews were conducted among purposively selected key informants using an interview guide. Thematic coding was applied by using a partially deductive approach, informed by findings from previous studies, and an inductive coding technique with the creation of themes as they emerged from the transcripts. Data were analyzed thematically using ATLAS.ti.<br>Result: Study participants responded that the main function of PMT is improving service delivery. The engagement of PMT on multiple committees that overloaded members with additional meetings and assignments had a negative impact on data use. Data use was thwarted by a lack of recognition, a lack of resources, poor quality routine data, insufficient human power, a lack of accountability, and inadequate follow-up.<br>Conclusion: The PMT's data use culture was influenced by myriad of organizational, behavioral, and technical factors. There is a need to improve health professionals' perceptions towards health data, and skills for incorporating data into daily work. Non-monetary motivation should be used to develop contextually relevant tactics to improve the PMT's data use culture.
**Order of Authors:**	Admas Abera, MSc in Epidemiology
	Tilahun Shiferaw, MSc in Information Science
	Abebe Tolera, MPH EPidemiology
	Biruk Shalmeno
	Adisu Birhanu Weldesenbet
	Assefa Tola
	Alemayehu Girma
	Rania Mohammed
	Yadeta Dessie
**Additional Information:**	
**Question**	**Response**

Powered by Editorial Manager® and ProduXion Manager® from Aries Systems Corporation

**Financial Disclosure**<br><br>Enter a financial disclosure statement that describes the sources of funding for the work included in this submission. Review the submission guidelines for detailed requirements. View published research articles from *PLOS ONE* for specific examples.<br><br>This statement is required for submission and **will appear in the published article** if the submission is accepted. Please make sure it is accurate.<br><br>**Unfunded studies**<br>Enter: *The author(s) received no specific funding for this work.*<br><br>**Funded studies**<br>Enter a statement with the following details:<br>• Initials of the authors who received each award<br>• Grant numbers awarded to each author<br>• The full name of each funder<br>• URL of each funder website<br>• Did the sponsors or funders play any role in the study design, data collection and analysis, decision to publish, or preparation of the manuscript?<br>• **NO** - Include this sentence at the end of your statement: *The funders had no role in study design, data collection and analysis, decision to publish, or preparation of the manuscript.*<br>• **YES** - Specify the role(s) played.<br><br>* typeset	This work would not be possible without the financial support of the Doris Duke Charitable Foundation (DDCF) under grant number 2017187. The mission of the Doris Duke Charitable Foundation is to improve the quality of people's lives through grants supporting the performing arts, environmental conservation, medical research, and child well-being, and through the preservation of the cultural and environmental legacy of Doris Duke's properties. The funders had no role in study design, data collection and analysis, decision to publish, or preparation of the manuscript. The funder website can be found here: https://www.ddcf.org/
**Competing Interests**<br><br>Use the instructions below to enter a competing interest statement for this submission. On behalf of all authors, disclose any competing interests that could be perceived to bias this work—acknowledging all financial support and any other relevant financial or non-financial competing interests.<br><br>This statement is required for submission	The authors have declared that no competing interests exist.

Powered by Editorial Manager® and ProduXion Manager® from Aries Systems Corporation

and **will appear in the published article** if the submission is accepted. Please make sure it is accurate and that any funding sources listed in your Funding Information later in the submission form are also declared in your Financial Disclosure statement.<br><br>View published research articles from *PLOS ONE* for specific examples.<br><br>**NO authors have competing interests**<br><br>Enter: *The authors have declared that no competing interests exist.*<br><br>**Authors with competing interests**<br><br>Enter competing interest details beginning with this statement:<br><br>*I have read the journal's policy and the authors of this manuscript have the following competing interests: [insert competing interests here]*<br><br>* typeset	
**Ethics Statement**<br><br>Enter an ethics statement for this submission. This statement is required if the study involved:<br><br>• Human participants<br>• Human specimens or tissue<br>• Vertebrate animals or cephalopods<br>• Vertebrate embryos or tissues<br>• Field research<br><br>Write "N/A" if the submission does not require an ethics statement.<br><br>General guidance is provided below. Consult the submission guidelines for detailed instructions. **Make sure that all information entered here is included in the Methods section of the manuscript.**	The ethical approval and clearance for this research was obtained from Institutional Health Research Ethics Review Committee (IHRERC) of Haramaya University with reference number IHRERC/196/2020. Permission was sought from regional health bureaus and the health facilities. Before data collection, informed written consent was obtained from the study participants after explaining the study aims and their right to withdraw from the study at any time.

**Format for specific study types**<br><br>**Human Subject Research (involving human participants and/or tissue)**<br>• Give the name of the institutional review board or ethics committee that approved the study<br>• Include the approval number and/or a statement indicating approval of this research<br>• Indicate the form of consent obtained (written/oral) or the reason that consent was not obtained (e.g. the data were analyzed anonymously)<br><br>**Animal Research (involving vertebrate animals, embryos or tissues)**<br>• Provide the name of the Institutional Animal Care and Use Committee (IACUC) or other relevant ethics board that reviewed the study protocol, and indicate whether they approved this research or granted a formal waiver of ethical approval<br>• Include an approval number if one was obtained<br>• If the study involved *non-human primates*, add *additional details* about animal welfare and steps taken to ameliorate suffering<br>• If anesthesia, euthanasia, or any kind of animal sacrifice is part of the study, include briefly which substances and/or methods were applied<br><br>**Field Research**<br><br>Include the following details if this study involves the collection of plant, animal, or other materials from a natural setting:<br>• Field permit number<br>• Name of the institution or relevant body that granted permission	
**Data Availability**<br><br>Authors are required to make all data underlying the findings described fully available, without restriction, and from the time of publication. PLOS allows rare exceptions to address legal and ethical concerns. See the PLOS Data Policy and FAQ for detailed information.	Yes - all data are fully available without restriction

A Data Availability Statement describing where the data can be found is required at submission. Your answers to this question constitute the Data Availability Statement and **will be published in the article**, if accepted.<br><br>**Important:** Stating 'data available on request from the author' is not sufficient. If your data are only available upon request, select 'No' for the first question and explain your exceptional situation in the text box.<br><br>Do the authors confirm that all data underlying the findings described in their manuscript are fully available without restriction?	
**Describe where the data may be found in full sentences. If you are copying our sample text, replace any instances of XXX with the appropriate details.**<br><br>• If the data are **held or will be held in a public repository**, include URLs, accession numbers or DOIs. If this information will only be available after acceptance, indicate this by ticking the box below. For example: *All XXX files are available from the XXX database (accession number(s) XXX, XXX.).*<br>• If the data are all contained **within the manuscript and/or Supporting Information files**, enter the following: *All relevant data are within the manuscript and its Supporting Information files.*<br>• If neither of these applies but you are able to provide **details of access elsewhere**, with or without limitations, please do so. For example:<br><br>*Data cannot be shared publicly because of [XXX]. Data are available from the XXX Institutional Data Access / Ethics Committee (contact via XXX) for researchers who meet the criteria for access to confidential data.*<br><br>*The data underlying the results presented in the study are available from (include the name of the third party*	All relevant data are within the manuscript.

and contact information or URL).<br>• This text is appropriate if the data are owned by a third party and authors do not have permission to share the data.<br><br>* typeset	
Additional data availability information:	

Powered by Editorial Manager® and ProduXion Manager® from Aries Systems Corporation

Full Manuscript
Click here to access/download;Manuscript;Challenges and facilitators of data use_PLOS ONE.docx

Experience, Challenges, and Facilitators of Health Data Use among Performance Monitoring Teams (PMT) of Health Facilities in Eastern Ethiopia: *A Qualitative Study.*

Admas Abera[1*], Abebe Tolera[1], Biruk Shalmeno[1], Adisu Birhanu Weldesenbet[1], Assefa Tola[1], Tilahun Shiferaw[2], Alemayehu Girma[3], Rania Mohammed[4] and Yadeta Dessie[1]

[1]Epidemiology and Biostatistics Unit, School of Public Health, College of Health and Medical Sciences, Haramaya University, Harar, Ethiopia.

[2]Department of Information Sciences, College of Computing and Informatics, Haramaya University, Haramaya, Ethiopia.

[3]Policy and Plan Directorate, Dire Dawa Administration Health Bureau, Dire Dawa, Ethiopia.

[4]Policy and Plan Directorate, Harari Regional Health Bureau, Harar, Ethiopia.

*Corresponding author:

Email: admasabera10@gmail.com

Abstract

Background: Using routine health data plays a crucial role in decisions towards improved health outcomes. Despite significant investments in improving Ethiopia's Performance Monitoring Teams (PMT), there is limited evidence on the facilitators and barriers to data utilization by this team in responding to present and developing health challenges. This study, therefore, is aimed at exploring PMT experiences, facilitators and barriers to information use in healthcare facilities in eastern Ethiopia.

Method: This study employed a phenomenological study design**.** Consolidated Framework for Implementation Research (CFIR) framework was adapted to identify the most relevant constructs with the aim of describing data use approaches. Key informant interviews were conducted among purposively selected key informants using an interview guide. Thematic coding was applied by using a partially deductive approach, informed by findings from previous studies, and an inductive coding technique with the creation of themes as they emerged from the transcripts. Data were analyzed thematically using ATLAS.ti.

Result: Study participants responded that the main function of PMT is improving service delivery. The engagement of PMT on multiple committees that overloaded members with additional meetings and assignments had a negative impact on data use. Data use was thwarted by a lack of recognition, a lack of resources, poor quality routine data, insufficient human power, a lack of accountability, and inadequate follow-up.

Conclusion: The PMT's data use culture was influenced by myriad of organizational, behavioral, and technical factors. There is a need to improve health professionals' perceptions towards health data, and skills for incorporating data into daily work. Non-monetary motivation should be used to develop contextually relevant tactics to improve the PMT's data use culture.

Key words: Data use, Performance monitoring teams, challenge and facilitators, healthcare facilities, Ethiopia.

Introduction

Health systems are complex and continually changing to adapt to changing political, economic, social, technological, and epidemiological realities within constrained resources, particularly in low and middle-income countries. (1-3) Health facilities need to cope with these changing realities through organized management and leadership, which require use of reliable data for the development of a comprehensive policy package for health sector reforms; improving planning and continuous monitoring. (2, 4)

There has been an increased need for strengthening health service performance to manage population needs through effective leadership (5-7) and improved health facility data use culture. [8-10] The increasing demand for and capacity to use data appears more important than the augmenting supply of evidence (3,13) for improved access to and quality of care. (4, 14) Collection, processing, transforming, communicating, and using service delivery data and administrative reports play crucial roles in decisions towards improved health outcomes. (15, 16)

Data use is the process through which decisions-makers and stakeholders explicitly evaluate information in one or more steps of the process of policymaking, program planning and management, or service provision. (6) In the Ethiopian context, the Performance Monitoring Team (PMT) is a team of multidisciplinary health workforce that is primarily responsible for data use. (11, 12)

Effective use of the data can enable targeted improvements in health service delivery to meet a community's needs. (17) However, the strength and quality of the evidence needed to get valid information to make decisions about health programs is either weak or insufficient in many developing countries (18-20) mainly due to ineffective data use culture. (20) Inadequate infrastructure, leadership turnover, dysfunctional external relations (26), poor production of information and limited resources (15) were identified as major bottlenecks for information use.

In addition to shortage of skilled workforce, imbalances in skill mix, geographical misdistribution, difficulty in inter-professional collaboration, inefficient use of resources, and burnout were found to affect service quality. (21) So far, substantial efforts to strengthen health information systems mainly focused on digitization, improving data quality and analysis, and identifying problems, but the ultimate goal is using information to solve problems, which requires building an information

use culture over time. (22) Therefore, engagement (including involvement, commitment, effort or observable behaviour, positive affect or some combination of these of healthcare leaders and managers is pivotal if we are to improve the Health Information System (HIS) and hence the health service delivery. (4, 13)

The engagement level and capacities needed by health managers and performance monitoring teams to respond to current and emerging issues are not yet well studied. (21) Without radical structural and systemic changes, existing governance structures and management systems will continue to fail to address the existing gaps in health service delivery (2) Furthermore, despite large investments in performance management team improvement in Ethiopia, a common performance renewal effort is lacking. Little is known about the factors that influence the use of health data to improve primary health care delivery in low- and middle-income countries. This study, therefore, is aimed at identifying experiences of the PMT, and facilitators and barriers to information use process in healthcare facilities in East Ethiopia.

Methods and Materials

Study Settings

The study was conducted in selected public health facilities of Dire Dawa and Harari regions, in Eastern part of Ethiopia. Located 515 kilometers from Addis Ababa, Dire Dawa has an estimated population of 341,834, with 68.23% of them living in urban area. There are about 15 health centers (8 urban and 7 rural), two hospitals, and 32 health posts under the city administration. There are a total of 622 health professionals and 209 health extension workers, and these health facilities serve a total of 480,000 people. Harari is one of the eleven regional states of Ethiopia, and is located about 517Km east of Addis Ababa and 48 km south of Dire Dawa. The Region has a total population of 183,415 people, of whom 92,316 are men and 91,099 are women. (23) The majority of its population live in urban areas, which accounts for 54.18% of the population. (24) There are three government hospitals, one university teaching hospital, two private hospitals, eight health centers, and 24 health posts in the region.

Study Design

This study used a phenomenological study design. Consolidated Framework for Implementation Research (CFIR) framework was adapted to identify the most relevant constructs with the aim of describing data use approaches. The CFIR framework is an evidence-based framework drawing from multiple disciplines that provides a comprehensive arrangement of paradigms influencing complex implementations. The framework has five major domains with associated components, all of which were used in this study.

Study participants and data collection

We conducted a key informant interview using a semi-structured interview tools. The guide is comprised of questions related to socio-demographic characteristics of the respondents, process of the PMT establishment, data use processes and practices, challenges to data use, motivating factors for data use, and recommended motivation mechanisms. The interview guides were prepared in English and translated to local languages (Amharic and Afan Oromo). The key informants include medical directors, health center heads, department heads (pharmacy, laboratory, nursing, human resource and finance team leads), and the HMIS officers.

Two hospitals and four health centers were randomly selected from list of facilities in the two regions. A purposive sampling technique was employed to identify the key informants. Around eighteen interviews with key informants were conducted based on level of data saturation. Data were collected between July 05, 2021 and July 24, 2021 by interviewers fluent in Amharic, Afan Oromo and English, with graduate level education and experience with qualitative data collection. All members of the data collection team were provided a one day workshop and training to have a common understanding of all aspects of the interview guide and objectives of the study. The data collection activities were supervised on daily basis by the study investigators. Overall 18 key informants were selected including medical directors, health center heads, HMIS officers, case team leaders, laboratory and pharmacy heads, and human resource unit heads. All interviews were audio-recorded in private place suitable for the respondent and the interviews last about 20-60 minutes.

Data Analysis

All interviews were tape-recorded, verbatim transcribed in the original language and translated into English. Transcripts were analyzed by two researchers (AA, AT) who developed a codebook which was validated by the whole research team. ATLAS.ti software was used to analyze the data thematically. Thematic coding was applied by using a partially deductive approach, informed by findings from previous studies, and an inductive coding technique with creation of themes as they emerged from the transcripts.

Ethical Considerations

The ethical approval and clearance for this research was obtained from Institutional Health Research Ethics Review Committee (IHRERC) of Haramaya University with reference number IHRERC/196/2020. Permission was sought from regional health bureaus and the health facilities. Before data collection, informed written consent was obtained from the study participants after explaining the study aims and their right to withdraw from the study at any time.

Results

Socio-demographic characteristics

A total of 18 in-depth interviews were conducted in 2 Hospitals and 4 health centers to explore the PMT experience, barriers and facilitators of data use. Of these participants 8 were females, 3 were health facility heads, and has median age (IQR) of 31 (7) years. (Table 1)

Table 1: Socio-demographic characteristics of key informants at the health facilities, Eastern Ethiopia 2021.

Participant ID	Sex	Age in years	Educational status	Work experience in years	Facility type
KI1	Female	34	BSc	11	Health center
KI2	Female	25	Diploma	2	Health center
KI3	Male	27	BSc	9	Health center
KI4	Female	38	BSc	20	Health center
KI5	Male	26	BSc	7	Health center
KI6	Male	34	BSc	13	Health center
KI7	Male	28	Master’s	3	Health center
KI8	Female	25	BSc	1	Health center
KI9	Female	29	BSc	6	Referral Hospital
KI10	Male	28	BSc	32	Referral Hospital
KI11	Male	32	BSc	13	Health center
KI12	Male	30	BSc	7	Health center
KI13	Female	34	BSc	8	Health center
KI14	Female	39	BSc	12	Health center
KI15	Male	42	BSc	19	General Hospital
KI16	Male	35	BSc	3	General Hospital
KI17	Female	35	BSc	12	General Hospital

KI18	Female	30	BSc	10	General Hospital

Key Themes emerged

The main themes that emerged from the analysis of the interview with PMT members were:

Key Theme 1: Current status and experience of PMT at the health facility

Key Theme 2: Barriers to data use in the facility

Key Theme 3: Motivational mechanisms and demotivating factors in enhancing data use practices in place for PMT members

Key theme 4: Recommended mechanisms to improve the data use practices

The current PMT status at the health facilities

In Ethiopia, PMT at health facilities is established based on the standards set by the Ministry of Health. In the study, participants stated that the health facilities use certain criteria to select the members of the PMT. The main criteria reported by the participants to establish the PMT at their health facility include being the head of the quality department, HMIS unit team leader, department head, management team, and staff with good performance.

A matron nurse at a hospital reported "*…since the performance of every department is reviewed in the meeting, the main criteria for PMT member is being a department head. The heads of management unit, HMIS unit and quality department are also included. I became a member because I serve as a vice matron for the last one and half year.*" (Matron Nurse, 29 years old)

Another case team leader from a health center stated

"*…any member of the case team can be a member of PMT. It is not necessarily the case team leader. That means there will be one representative from each case team in PMT in respective of his/her status.*" (Program planning team leader, 42 years old)

Roles and responsibilities of PMT members is to use health data and improve the service performance of the health facilities. The specific roles of PMT includes preparing action plan, put directions to improve the service delivery, compare performance overtime, monitor monthly

performance, provide feedback to other employees, support staffs with skill gap and preparing the health facility regular reports. The present study also found that the PMT members are aware of their responsibilities.

> One of the participants stated that "…*each member of the PMT is expected to prepare and submit their case team or department reports to the head of the health facility on time. Then all PMT members will discuss on the submitted report during the monthly PMT meeting in which the reports are crosschecked for its consistency and quality. In general, the main function of PMT in our facility is monitoring the performance of each unit. When the achievements are lower than expected, the reasons for underachievement are sorted out and future directions are put to improve the performance*." (EPI focal person, 38 years old)

The participants' opinions on the alignment of the PMT's roles and responsibilities at the facility with their activities were mixed. While the majority of respondents stated that PMT tasks and responsibilities are totally matched with their actual activities, some stated that they are not completely aligned with their actual activities. There is overlapping of duties and roles, according to these participants.

The benefits of PMT for the facility include improving the service delivery and customer satisfaction. In addition, PMT is vital for maintaining the well-functioning of the departments and case teams. Respondents understand the benefits of PMT for facilities, which include planning, identifying gaps and intervening on the problem, ensuring the facility's progress to success, and monitoring and evaluating program implementations.

The modality of PMT meetings at the health facility was the other focus area studied. This subtheme investigated the adequacy and convenience of meeting time, the level of PMT member participation, meeting focus areas, and the effect of multiple committees in the facility on PMT.

Adequacy and convenience of time of PMT meeting is essential in order to identify the most important problems in the facility. In this study, there were contrasting views regarding the adequacy and convenience of meeting time. The majority of the study participants said there is monthly meeting plan in their health facilities. This meeting is usually prepared in advance on the

annual plan. This annual PMT meeting schedule is usually posted in visible area. Most of the respondents reported that the monthly meetings were conducted immediately after each unit submitted their monthly report. In some health facilities, there is also good practice for announcing any changes in meeting days in advance.

> *"Our PMT meeting is conducted on monthly basis. Immediately after the submission of the monthly report (usually at 21st day of the month), we hold our PMT meeting at 23th or 24th day of the month. Substitution date will be posted on our telegram page if the meeting is not conducted based on the schedule."* (Pharmacy head, 30 years old)

On the other hand, some study participants indicated that the time allocated for the meetings was inadequate and inconvenient. Sometimes these meetings were conducted during weekends or when PMT members are occupied with other duties. Some participants discussed how holding the meeting on weekends could reduce absenteeism and unnecessary interruptions. . All the participants anonymously agreed that enough time should be assigned and convenient time should be selected to conducting the meeting. Participants also reported that conducting the meeting during the working hours is usually inconvenient.

> "…*previously, we used to hold the PMT meeting during working hours and hence the duration of the meetings was not adequate. This was due to high work load of the PMT members. However, currently we are conducting our PMT meeting on Saturdays. Since it is not a working day, we are paying some pocket money for the PMT members."* (OPD case team leader, 25 years old)

The focuses of PMT meetings reported include data quality, performance activities (monthly, quarterly or annual performance), service quality, and the service improvement strategies. According the participants, the first agenda of the PMT meeting is evaluating the previous report, and then comparing it with the current performance. Afterward, the team develops an action plan based on the identified gaps. However, in some health facility the previous performance is not conducted at all.

> A participant from a hospital stated "*In our monthly PMT meeting, the first thing is presenting the monthly performance report each units. Based on the report, the gaps and*

challenges are identified, before action plan are drafted on the identified gaps". (Matron Nurse, 29 years old)

The PMTs mostly utilize routine data for evaluation and monitoring of service delivery programs. There was no culture of using health data from other sources such as surveys, assessments, research findings.

Another participant from a health center reported "*In our health center, the main target of PMT is the routine data. The data accuracy and completeness are checked. Service improvement strategies are also discussed.*" (TB/HIV focal person, 26 years old)

Most study participants stated that poor tracking of problems and monitoring action plans is frequently observed. No adequate monitoring is made to check progress of identified problems for the next meeting. Despite this, some study participants reported that presence of good participation by PMT members during action plan implementation. Absenteeism and interruption of the meeting were the main challenges during PMT meeting according to the participants.

Effect of multiple committees in the facility on PMT Most participants reported that they involve in at least two other committees in their health facility. This in turn caused: poor follow up of activities set out by the PMT; being overburdened by committee meetings and assignments; creates fatigue and shortage of time to accomplish PMT assignments. Being involved in multiple committees can negatively affect the effectiveness of the PMT activities.

"…PMT members have their own duties in addition to the PMT which makes it difficult to collect quality data due to competing priorities. If someone is engaged with overlapping duties, the desired outcome may not be achieved". (Laboratory head, 27 years old)

A health center HMIS officer reported "*…being the member of several committees affects the PMT activities. The aim of including a person in a given team or committee is to perform the task effectively and efficiently in order to bring the intended changes. However, the focus of the individual is usually divided to different committees. In most cases the meeting times ~~is overlapping~~ between different committees"* (HMIS officer, 25 years old)

Major barriers to data use in the facility

Lack of understanding of data value by healthcare workers was indicated as the major challenge faced when using data for action. According the participants, most of health professionals have poor understanding and poor attitude on the importance of data in improving quality of health services. Skill gaps in analyzing data using electronic computing software such as DHIS2 were also mentioned by the participants. The overall barriers PMT members faced when using data for decision making are categorized under the following sub-themes.

Organizational barriers

The main organizational barriers to data use at the facility level were poor data quality, competing priorities (being a members of many committees and high patient flow), human resource related issues (shortage, lack of adequate skills among PMT, etc.), input related issues (ICT, registers, logbooks, guidelines, etc.), infrastructure related issues (inadequate working space), inadequate budget allocation, as well as lack of performance based incentives, recognition and motivation mechanisms.

The quality of the routine data collected at health facilities has been reported as a barrier for effective data use.

> *"There is a gap in data quality including data inconsistency and incompleteness. Healthcare providers perform their daily activity but they do not document it on the register regularly. The data from HMIS may contradict from your observation every day. Hence it is difficult to use our data for decision making due to its poor quality."* (HMIS officer, 25 years old)
>
> Another participant also reported that *"A timely, reliable and high-quality data should be generated in order to use the data for decision. Monitoring and supporting the staffs is also critical to obtain high quality data."* (MCH department head, 35 years old)

Working environment Respondents also mentioned unsuitable working environment and work overload in recent years to have significantly affected data quality and use.

“This hospital building was constructed in 1950s. At that time, the population of Dire Dawa was estimated to be less than hundred thousand. Currently, the city population alone surpasses five hundred thousand. So, the infrastructure is not suitable to perform our activities including HIS activities.” (Quality team leader, 35 years old)

Input related factors Despite some health facilities printing their own registers, others reported that they usually faced shortage of the registration books.

A supervisor of health extension workers stated *“we have an old register called daily register for documentation of our data. This register was supplied by the NGO. However, currently this funding has stopped and we faced shortage of the daily register. This shortage affected our documentation.*” (HEW supervisor, 34 years old)

Incentives, Recognitions and motivational mechanism in the health facility can promote employees to strive for better performance. Absence of performance-based incentives, recognition and motivation mechanisms was one of the main organizational challenges mentioned participants. If the health facility designs performance-based recognition and motivational programs related to data use, it has the potential to promote others to improve their data use culture. While participants reported that some sort of recognitions and motivation mechanisms were implemented in their facilities, others believe that incentive ~~by themselves~~ may not necessarily motivate staffs to use the data or improve service performance.

Healthcare workers behavioral barriers

Poor of commitment of PMT members, knowledge and attitude gap of managers or other health workers, lack of accountability for failing to accomplish HIS tasks, poor documentation practice, and resource mismanagement were cited as challenges for an effective information use.

Poor commitment and accountability from healthcare providers and PMT members were the most commonly reported challenge. One respondents ~~from hospital~~ explained ~~regarding the~~ commitment and competing priorities of PMT members as

“…sometimes we reviewed the same problem repeatedly without a solution. We start to get fed up and start to wonder when it will be solved. Sometimes, we skip the meeting

intentionally for this reason. In addition, some of us are leading three to four case teams and ~~involving~~ in several committees in the facility. (Matron Nurse, 29 years old)

Poor documentation Practice The other challenge was poor documentation practice of healthcare providers. Some staffs do not properly record the service provided on the registries. Staff attitude towards data was also mentioned as a challenge for evidence based decision making. In addition, some staffs carry out their routine activities such as registering and reporting carelessly. The participants suggested that in order to change data use culture, the first priority should be changing the attitude of the health care workers before other interventions are introduced.

"I*t is hard to say that presence of incentives only positively affects data use. Health professionals should believe documenting the data primarily benefits patients, themselves and their health facility. Therefore, working on the attitude change should come first. Providing incentives before attitude change may even adversely affect the data use practice."* (Health center head, 32 years old)

COVID19 effect on data use COVID-19 pandemic directly and indirectly affected data use practices at the health facilities. An informant explained the effect of COVID-19 pandemic on their day-to-day activity as

"*Due to the occurrence of COVID -19 outbreaks, the nearby hospital entirely devoted to provide health care service for COVID-19 cases. Hence, all patients in the catchment area of that hospital came to our health center. These conditions increased the work load and compromised our routine activities*." (EPI focal person, 39 years old)

Motivation mechanism in place for PMT members at the facilities

This study identified several motivation mechanisms in place though not ~~all~~ across all facilities as well as not conducted in a regular manner. The main motivation strategies reported were capacity building and performance-based recognition.

Capacity building practices for PMT members According the participants the main capacity building practices for PMT members were career development opportunities, in-service trainings,

recognition for good performance, appropriate selection of training participants, high quality of trainings, post training follow up, and linked to professional development of the health workers.

Recognition and non-monetary incentives The participants listed several non-monetary incentive mechanisms that can motivate the PMT members in the facility. The main non-monetary are provision of mobile cards, and recognition of good performers, as well as awarding the facilities based by regional health bureau. Participants also indicated that these motivational mechanisms become effective only as far as the information use culture of the facilities is improved.

> A participant suggested that "*Performance-based recognition and motivation of staffs can improve the data quality and subsequently the data use in the facility.*" (Health Center head, 34 years old)

However, in some health facilities there were no any motivation measures specific to PMT members.

> *"There are no capacity buildings, recognition mechanisms, and career enhancing educational chance to motivate PMT members."* (TB/HIV focal person, 38 years old)

Demotivating factors in enhancing data use practices

Several demotivating factors were cited by the study participants. The main demotivating factors include shortages of resources, poor quality of routine data, insufficient human power, and lack of accountability, inadequate salary, and inadequate follow up. Some study participants mentioned the follow up from Woreda or regional health bureau was not adequate.

> "*salary is one of the demotivating factors because it does not fit with the job we undertake.*" (NCD focal person, 28 years old)

The leadership skill of the managers was one of the factors to promote the data use in health facilities. Health managers' and case-team/departments' poor leadership skills, and lack of value for data were also mentioned as demotivating factors.

> "*If the head of the department or the facility do not utilize data properly, other employees will not care about data use. Hence, the head at woreda or region or at any other level should be*

~~example~~ *for the other staffs in order to improve the data use culture."* (HMIS officer, 25 years old)

Recommended motivation mechanisms for an improved data use mentioned in this study include performance-based recognition, arranging meeting sites and time, capacity building, continuous supply of resources, improving the infrastructures, and strengthening the HIS.

> "*Since the PMT members are the backbone of the health facilities, designing motivating mechanisms is important. Ensuring continuous supply of necessary materials, and recognizing best performers should be introduced.*" (Matron Nurse, 29 years old)

Discussion

Adopting the Consolidated Framework for Implementation Research (CFIR) framework, the present study aimed to explore experiences, facilitators and barriers of PMT to use information for decision making among healthcare facilities in Eastern Ethiopia. The study found that

performance monitoring teams in most facilities were established according to the standard criteria for establishment set by the Ministry of Health (MOH) of Ethiopia.(12) The PMT members in this study also reported that the main role and responsibility of PMT was to improve the performance of the health facilities through identifying gaps, preparing improvement action plans, and monitoring performance. Previous studies also indicated the primary responsibility of PMT is to improve data quality and use information regularly to monitor progress and improve health service performance. (11, 12) Few PMT members also did not have clear ideas about how they could align their roles and responsibilities with their usual professional duties. The national guideline clearly indicates that roles responsibilities must be assigned to members of PMTs through terms of references and membership assignment letters. (12)

Participants in the study had differing opinions on the adequacy and convenience of the PMT meetings. Some of the participants stated that the time allotted for meetings was insufficient and inconvenient, requiring health facilities to schedule some meeting sessions on weekends to reduce absenteeism and undue disruption.

In line with the national guideline, although some irregularities were reported, the meetings were conducted monthly immediately after each unit submitted their service and diseases reports, and before submitting the report to the next level to monitor progress and improve performance. The MOH guideline indicates that the meeting dates, venue and its members should be officially communicated in advance and the meeting should be conducted at least a day ahead of submission of the monthly report to the next level. (11, 12, 25)

The present study also found that the major focus of PMT meeting includes data quality, performance, service quality, and patient and staff satisfaction. Evaluation of the previous reports was identified as the first agenda of the monthly meetings. However, in some health facilities, there was no assessment of the previous month's performance. Follow-up activities for gaps highlighted in the previous PMT meeting should always be the first item on the agenda, followed by an assessment of progress on those gaps. (12, 25) Meetings and collected data have no value in themselves. There ~~are~~ enough evidence ~~that indicate~~ the value and relevance of meetings come only after analysis, and transformation into meaningful actions. (26)

Poor tracking of the gaps identified by the PMT, including poor follow-up and inadequate monitoring of action plans on identified problems were the other findings. In some health facilities, only few dedicated PMT members were participating regularly in PMT meetings. This might be associated with work overload of the PMT. Most members are usually involved in at least two other committees in their health facility. This has its own share of poor follow up of activities set out by the PMT; being overburdened by committee meetings and assignments; creating fatigue and a shortage of time to accomplish PMT assignments. Health facilities should design strategies to minimize the number of committees and integrate similar committees to improve their performance.

Poor practice of information use from external sources was also a robust finding in the present study. Routine internal data was the only source for most of the decisions that the PMT members use. Evidence indicate data triangulation through the use of other sources such as original research, community feedback, expert opinions, vital registration, censuses and routine HMIS data can yield better results. (12, 25, 26)

The major challenges of data use emanate from organizational, behavioral and environmental sources. In our study, the main organizational challenges that negatively affect data use at the facility level were poor data quality, competing priorities, shortage of skilled human power, inadequate budget allocation for data use, and lack of performance-based incentives, recognition and motivation mechanisms. Previous studies indicated that an organizational context that supports data collection, availability, and use, the technical aspects of data processes and tools, and the behavior of individuals who produce and/or use data are the main elements of health information use. (25, 27)

Healthcare organizations are increasingly required to gather and report data about their performance and respond to the results of consequential quality measurements. (28) Excellence in data quality enables healthcare organizations to plan and provide effective and efficient service for users and to meet their targets. (29) Our study revealed that the poor quality of data was one of the major organizational challenges for using information for decision making in the health facilities. A mixed-methods study in Addis Ababa indicated that the PMT meetings that were designed for the sole purpose of improving data quality are not effective. (30) Another qualitative study that

explored the barriers and facilitators of digital health technology use also identified data quality as a potential barrier for data use. (31) Lack of available data for several indicators and a lack of validated indicators for important dimensions of quality PHC were identified as major challenges to improving the Primary Health Care (PHC) Performance. (6)

Evidences suggest lack of standards with respect to data quality was one of the organizational factors that had a direct influence on the use of data. (25) Other organizational challenges were shortages of essential inputs like ICT, registers, logbooks, and guidelines. In another study, inadequate ICT infrastructure and computer deployment were the main reasons for the low use of information. (26)

In the present study, lack of commitment, knowledge and attitude gap, lack of accountability for failing to accomplish tasks, poor understanding of data value, skill gaps, work overload, poor documentation, and resource mismanagement were the main individual level challenges identified for information use at facility level in this study. Behavioral factors provide crucial insight into the way in which health workers, managers and policymakers use information. (25) The attitude, motivation, and value the decision makers attach to information play a big role in determining the data use. (2, 25) Healthcare workers can be motivated to perform their duty if they are properly appreciated, encouraged, supported, recognized, respected, and admired by supervisors, colleagues, clients, family, and the community. (32)

The PMT members have a number of competing priorities. In addition to being member of the PMT, they have their own duties and responsibilities. This increased their workload and compromised the effectiveness of data use at facility level. Participation in quality improvement collaborative activities may improve health professionals' knowledge, problem-solving skills and attitude; teamwork; shared leadership and habits for improvement. A review of literature indicated that shifting roles and expectations in the workplace for health care leadership and management as major challenge for health leadership and workforce. (1) Interaction across quality improvement teams may generate normative pressure and opportunities for capacity building and peer recognition. (11)

Health professionals either do not consider recording and reporting data as part of their routine activities or they give more priority to the clinical provision and lesser attention to data. (30)

Hence, it is mandatory to work on the attitude and knowledge of health professionals to enhance the value they attach to data. Although there is a need to make significant investments in workforce development and training (33), tackling of behavioral factors require interventions that go beyond simple trainings and should focus more on initiatives that enhances positive attitude towards data value. (25)

The individual determinant of motivation among healthcare workers are altruism, attaining professionalism, educational opportunity, and being more experienced. (32) In the present study, health facilities use various motivational mechanisms including capacity buildings, recognition and non-monetary incentives to improve performance. Career development opportunities, in-service trainings, and regular recognition for good performance were also reported to be practiced at the health facilities.

Provision of inputs necessary for HIS such as laptops, internet modems; as well as awarding the best performing health facilities were other motivational methods indicated in our study. Since health professionals can be motivated by a range of extrinsic and intrinsic factors, policy makers need to look beyond traditional financial incentives when designing policies to improve health service performance. (34) Studies indicate training opportunities, transformative leaders, and nonmonetary incentives, staff development and promotion, availing the necessary resources, and ease of communication are found to be effective motivation mechanisms for health care workers. (32, 35)

On the other hand, health workers reported the desire to engage in on-the-job learning, workshops, seminars, refresher courses and continuous training. (32) Incentives usually target one aspect of behaviour, but they are introduced into a complex system with an existing set of cultures and constraints that may affect the willingness and ability of practitioners to respond as intended. This makes it difficult to predict the outcome of any intervention, or to generalize about the transferability of findings across systems. (34) Savage et al. recommended healthcare leadership would benefit from a more integrative model of management which requires participatory leadership enabled through long-term investments at the individual, organizational and system levels. (12)

Conclusion

This study found that various organizational, behavioral and technical factors affected the data use culture of the health facilities. Poor data quality was also one of the major challenges reported for using data by the PMT in this study. Moreover, competing priorities and presence of multiple committees in facilities overburdened PMT members which has a negative influence on effective data use. The use of health data should be viewed as a public good and that achieving the broad benefits will require understanding and support from all concerned bodies. The non-monetary means of motivation should be used to create contextually relevant strategies to enhance the data use cultures. Different motivational strategies such as capacity building programs, recognition and non-monetary incentives were recommended to improve the performance.

Acknowledgements

The authors are thankful to Ministry of Health of Ethiopia, Dire Dawa and Harari regions health bureaus, JSI-Data Use Partnership Ethiopia, health facilities, data collectors, and study participants.

Reference

1. Figueroa CA, Harrison R, Chauhan A, Meyer L. Priorities and challenges for health leadership and workforce management globally: a rapid review. *BMC Health Services Research*. 2019;19(1):239.

2. Senkubuge F, Modisenyane M, Bishaw T. Strengthening health systems by health sector reforms. *Global health action*. 2014;7(1):23568.

3. Witter S, Anderson I, Annear P, Awosusi A, Bhandari NN, Brikci N, et al. What, why and how do health systems learn from one another? Insights from eight low- and middle-income country case studies. *Health Research Policy and Systems*. 2019;17(1):9.

4. Nordsteien A, Byström K. Transitions in workplace information practices and culture. *Journal of Documentation*. 2018;74(4):827-43.

5. Veillard J, Cowling K, Bitton A, Ratcliffe H, Kimball M, Barkley S, et al. Better measurement for performance improvement in Low-and Middle-Income countries: the primary health care performance Initiative (PHCPI) experience of conceptual framework development and indicator selection. *The Milbank quarterly*. 2017;95(4):836-83.

6. Tantivess S, Walt G. The role of state and non-state actors in the policy process: the contribution of policy networks to the scale-up of antiretroviral therapy in Thailand. *Health policy and planning*. 2008;23(5):328-38.

7. Fryatt R, Bennett S, Soucat A. Health sector governance: should we be investing more? *BMJ global health*. 2017;2(2):e000343.

8. Lippeveld T. Routine health facility and community information systems: creating an information use culture. Global Health: Science and Practice; 2017. p. 338-40.

9. Nordsteien A, Byström K. Transitions in workplace information practices and culture: The influence of newcomers on information use in healthcare. *Journal of Documentation*. 2018.

10. Jylhä V. Information management in health care: a model for connecting information culture and patient safety. 2017.

11. Adane T, Tadesse T, Endazenaw G. Assessment on Utilization of Health Management Information System at Public Health Centers Addis Ababa City Administrative, Ethiopia. *Internet Things Cloud Comput*. 2017;5(1):7-18.

12. Health FMo. Information Use Training Module. In: Policy PaMED, editor. Addis AbabaJune 2018.

13. Aqil A, Hozumi D, Lippeveld T. Tools for data demand and use in the health sector: Performance of Routine Information Systems Management (PRISM) Tools. *Measure Evaluation*. 2011.

14. Watson DE, Broemeling A-M, Reid RJ, Black C. A results-based logic model for primary health care : laying an evidence-based foundation to guide performance measurement, monitoring and evaluation. 2004.

15. Endriyas M, Alano A, Mekonnen E, Ayele S, Kelaye T, Shiferaw M, et al. Understanding performance data: health management information system data accuracy in Southern Nations Nationalities and People's Region, Ethiopia. *BMC Health Services Research*. 2019;19(1):175.

16. Health I, Quality A. Guidance on developing key performance indicators and minimum data sets to monitor healthcare quality. 2013.

17. Rendell N, Lokuge K, Rosewell A, Field E. Factors That Influence Data Use to Improve Health Service Delivery in Low- and Middle-Income Countries. *Global Health: Science and Practice*. 2020;8(3):566.

18. Maphumulo WT, Bhengu BR. Challenges of quality improvement in the healthcare of South Africa post-apartheid: A critical review. *Curationis*. 2019;42(1):1-9.

19. Lee CH, Yoon H-J. Medical big data: promise and challenges. *Kidney research and clinical practice*. 2017;36(1):3.

20. Zamboni K, Baker U, Tyagi M, Schellenberg J, Hill Z, Hanson C. How and under what circumstances do quality improvement collaboratives lead to better outcomes? A systematic review. *Implementation Science*. 2020;15(1):27.

21. West M, Dawson J. Employee engagement and NHS performance: King's Fund London; 2012.

22. Lippeveld T. Routine Health Facility and Community Information Systems: Creating an Information Use Culture. *Global Health: Science and Practice*. 2017;5(3):338.

23. CSA. POPULATION and HOUSING CENSUS OF ETHIOPIA. 2007.

24. Commission PC. Summary and statistical report of the 2007 population and housing census. Population size by age and sex. 2008.

25. MOH. Information use participants manual,Training Module. In: Policy Pam, and evaluation Directorate editor. Addis Ababa JUNE, 2018.

26. Obwocha W, Ayodo G, Nyangura A, Thomas O. Utilization of Healthcare Information Among Healthcare Workers in Gucha Subcounty, Kisii County, Kenya. *J Health Edu Res Dev*. 2016;4(192):2.

27. Vaughn VM, Saint S, Krein SL, Forman JH, Meddings J, Ameling J, et al. Characteristics of healthcare organisations struggling to improve quality: results from a systematic review of qualitative studies. *BMJ Quality & Safety*. 2019;28(1):74.

28. Pine KH, Bossen C, Chen Y, Ellingsen G, Grisot M, Mazmanian M, et al., editors. Data work in healthcare: challenges for patients, clinicians and administrators 2018.

29. MOH. Health Data Quality Training Module Participant Manual. In: Policy P, and monitoring and evaluation Directorate editor. Addis Ababa: Federal democratic republic of Ethiopia ministry of health; Noveber 2018.

30. Haftu B, Taye G, Ayele W, Habtamu T, Biruk E. A mixed-methods assessment of routine health information system (RHIS) data quality and factors affecting it, Addis Ababa City administration, Ethiopia, 2020. *Ethiopian Journal of Health Development*. 2021;35(1).

31. Slevin P, Kessie T, Cullen J, Butler MW, Donnelly SC, Caulfield B. Exploring the barriers and facilitators for the use of digital health technologies for the management of COPD: a qualitative study of clinician perceptions. *Qjm*. 2020;113(3):163-72.

32. Muthuri RNDK, Senkubuge F, Hongoro C, editors. Determinants of motivation among healthcare workers in the East African Community between 2009–2019: a systematic review 2020: Multidisciplinary Digital Publishing Institute.

33. Hripcsak G, Bloomrosen M, FlatelyBrennan P, Chute CG, Cimino J, Detmer DE, et al. Health data use, stewardship, and governance: ongoing gaps and challenges: a report from AMIA's 2012 Health Policy Meeting. *Journal of the American Medical Informatics Association*. 2014;21(2):204-11.

34. Lagarde M, Huicho L, Papanicolas I. Motivating provision of high quality care: it is not all about the money. *bmj*. 2019;366.

35. Weldegebriel Z, Ejigu Y, Weldegebreal F, Woldie M. Motivation of health workers and associated factors in public hospitals of West Amhara, Northwest Ethiopia. *Patient Prefer Adherence*. 2016;10:159-69.
