# Supplementary material for: Experiences, barriers, and facilitators of health data use among performance monitoring teams (PMT) of health facilities in Eastern Ethiopia: A qualitative study
Source: PLoS One. 2023 May 11;18(5):e0285662. doi: 10.1371/journal.pone.0285662 (PMC10174501; doi:10.1371/journal.pone.0285662)
Supplement: Attachment — Submitted filename: 1 PONE-D-22-10622_R1 (2).pdf [file pone.0285662.s004.pdf]

PLOS ONE

Experience, Barriers, and Facilitators of Health Data Use among Performance Monitoring Teams (PMT) of Health Facilities in Eastern Ethiopia: A Qualitative Study.

--Manuscript Draft--

**Manuscript Number:**	PONE-D-22-10622R1
**Article Type:**	Research Article
**Full Title:**	Experience, Barriers, and Facilitators of Health Data Use among Performance Monitoring Teams (PMT) of Health Facilities in Eastern Ethiopia: A Qualitative Study.
**Short Title:**	Barriers and facilitators of health data use by Performance Monitoring Teams
**Corresponding Author:**	Admas Abera, MSc in Epidemiology<br>Haramaya University College of Health and Medical Sciences<br>HARAR, ETHIOPIA
**Keywords:**	Performance monitoring team, data use, barriers, facilitators, health facilities, Ethiopia.
**Abstract:**	Abstract<br>Background: Using routine health data plays a crucial role in decisions towards improved health outcomes. Despite significant investments in improving Ethiopia's Performance Monitoring Team (PMT), there is limited evidence on the level of involvement, implementation strategies as well as the facilitators and barriers to data utilization by these teams in responding to present and emerging health challenges. This study, therefore, is aimed at exploring PMT experiences, facilitators and barriers to information use in healthcare facilities in Eastern Ethiopia.<br>Method: This study employed a phenomenological study design . Consolidated Framework for Implementation Research (CFIR) framework was adapted to identify the most relevant constructs with the aim of describing data use approaches at six facilities in Dire Dawa and Harari regions in July, 2021. Key informant interview was conducted among 18 purposively selected key informants using a semi-structured interview guide. Thematic coding was applied by using a partially deductive approach, informed by findings from previous studies, and an inductive coding technique with the creation of themes as they emerged from the transcripts. Data were analyzed thematically using ATLAS.ti.<br>Results: Study participants responded that the main function of PMT is improving service delivery. This study also revealed that data quality, performance, service quality and improvement strategies were among the major focus areas of the PMT. Data use by the PMT was affected by poor quality routine data, lack of accountability, and lack of recognition for good performers. The engagement of PMT members on multiple committees had a negative impact on data use that led to inadequate follow-up of PMT activities, weariness, and lack of time to complete responsibilities.<br>Conclusion: Performance monitoring teams in health facilities were established and functioning according to the national standard. Barriers to effective data use included engagement of PMT with multiple committees, poor quality of collected data, lack of accountability, and poor documentation practices. Addressing the potential barriers by leveraging the PMT and existing structures have the potential to improve data use and health service performance.
**Order of Authors:**	Admas Abera, MSc in Epidemiology
	Tilahun Shiferaw, MSc in Information Science
	Abebe Tolera, MPH EPidemiology
	Biruk Shalmeno
	Adisu Birhanu Weldesenbet
	Assefa Tola
	Alemayehu Girma
	Rania Mohammed
	Yadeta Dessie

Powered by Editorial Manager® and ProduXion Manager® from Aries Systems Corporation

Response to Reviewers:	All the comments raised by the reviewer have been addressed and attached in a point-by-point response in the attached "response to Reviewers" word document. Kindly refer the responses on the document as they are detail and long. Thank you.
Additional Information:	
**Question**	**Response**
**Financial Disclosure**<br><br>Enter a financial disclosure statement that describes the sources of funding for the work included in this submission. Review the submission guidelines for detailed requirements. View published research articles from *PLOS ONE* for specific examples.<br><br>This statement is required for submission and **will appear in the published article** if the submission is accepted. Please make sure it is accurate.<br><br>**Unfunded studies**<br>Enter: *The author(s) received no specific funding for this work.*<br><br>**Funded studies**<br>Enter a statement with the following details:<br>• Initials of the authors who received each award<br>• Grant numbers awarded to each author<br>• The full name of each funder<br>• URL of each funder website<br>• Did the sponsors or funders play any role in the study design, data collection and analysis, decision to publish, or preparation of the manuscript?<br>• **NO** - Include this sentence at the end of your statement: *The funders had no role in study design, data collection and analysis, decision to publish, or preparation of the manuscript.*<br>• **YES** - Specify the role(s) played.<br><br>* typeset	This work would not be possible without the financial support of the Doris Duke Charitable Foundation (DDCF) under grant number 2017187. The mission of the Doris Duke Charitable Foundation is to improve the quality of people's lives through grants supporting the performing arts, environmental conservation, medical research, and child well-being, and through the preservation of the cultural and environmental legacy of Doris Duke's properties. The funders had no role in study design, data collection and analysis, decision to publish, or preparation of the manuscript. The funder website can be found here: https://www.ddcf.org/
**Competing Interests**<br><br>Use the instructions below to enter a competing interest statement for this submission. On behalf of all authors, disclose any competing interests that could be perceived to bias this work—acknowledging all financial support	The authors have declared that no competing interests exist.

and any other relevant financial or non-financial competing interests.<br><br>This statement is required for submission and **will appear in the published article** if the submission is accepted. Please make sure it is accurate and that any funding sources listed in your Funding Information later in the submission form are also declared in your Financial Disclosure statement.<br><br>View published research articles from *PLOS ONE* for specific examples.<br><br>**NO authors have competing interests**<br><br>Enter: *The authors have declared that no competing interests exist.*<br><br>**Authors with competing interests**<br><br>Enter competing interest details beginning with this statement:<br><br>*I have read the journal's policy and the authors of this manuscript have the following competing interests: [insert competing interests here]*<br><br>* typeset	
**Ethics Statement**<br><br>Enter an ethics statement for this submission. This statement is required if the study involved:<br><br>• Human participants<br>• Human specimens or tissue<br>• Vertebrate animals or cephalopods<br>• Vertebrate embryos or tissues<br>• Field research<br><br>Write "N/A" if the submission does not require an ethics statement.<br><br>General guidance is provided below.	The ethical approval and clearance for this research was obtained from Institutional Health Research Ethics Review Committee (IHRERC) of Haramaya University with reference number IHRERC/196/2020. Permission was sought from regional health bureaus and the health facilities. Before data collection, informed written consent was obtained from the study participants after explaining the study aims and their right to withdraw from the study at any time.

Powered by Editorial Manager® and ProduXion Manager® from Aries Systems Corporation

Consult the submission guidelines for detailed instructions. **Make sure that all information entered here is included in the Methods section of the manuscript.**<br><br>**Format for specific study types**<br><br>**Human Subject Research (involving human participants and/or tissue)**<br>• Give the name of the institutional review board or ethics committee that approved the study<br>• Include the approval number and/or a statement indicating approval of this research<br>• Indicate the form of consent obtained (written/oral) or the reason that consent was not obtained (e.g. the data were analyzed anonymously)<br><br>**Animal Research (involving vertebrate animals, embryos or tissues)**<br>• Provide the name of the Institutional Animal Care and Use Committee (IACUC) or other relevant ethics board that reviewed the study protocol, and indicate whether they approved this research or granted a formal waiver of ethical approval<br>• Include an approval number if one was obtained<br>• If the study involved *non-human primates*, add *additional details* about animal welfare and steps taken to ameliorate suffering<br>• If anesthesia, euthanasia, or any kind of animal sacrifice is part of the study, include briefly which substances and/or methods were applied<br><br>**Field Research**<br><br>Include the following details if this study involves the collection of plant, animal, or other materials from a natural setting:<br>• Field permit number<br>• Name of the institution or relevant body that granted permission	
**Data Availability**<br><br>Authors are required to make all data underlying the findings described fully available, without restriction, and from the	Yes - all data are fully available without restriction

Powered by Editorial Manager® and ProduXion Manager® from Aries Systems Corporation

time of publication. PLOS allows rare exceptions to address legal and ethical concerns. See the PLOS Data Policy and FAQ for detailed information.<br><br>A Data Availability Statement describing where the data can be found is required at submission. Your answers to this question constitute the Data Availability Statement and **will be published in the article**, if accepted.<br><br>**Important:** Stating 'data available on request from the author' is not sufficient. If your data are only available upon request, select 'No' for the first question and explain your exceptional situation in the text box.<br><br>Do the authors confirm that all data underlying the findings described in their manuscript are fully available without restriction?	
**Describe where the data may be found in full sentences. If you are copying our sample text, replace any instances of XXX with the appropriate details.**<br><br>• If the data are **held or will be held in a public repository**, include URLs, accession numbers or DOIs. If this information will only be available after acceptance, indicate this by ticking the box below. For example: *All XXX files are available from the XXX database (accession number(s) XXX, XXX.*).<br>• If the data are all contained **within the manuscript and/or Supporting Information files**, enter the following: *All relevant data are within the manuscript and its Supporting Information files.*<br>• If neither of these applies but you are able to provide **details of access elsewhere**, with or without limitations, please do so. For example:<br><br>*Data cannot be shared publicly because of [XXX]. Data are available from the XXX Institutional Data Access / Ethics Committee (contact via XXX) for*	All relevant data are within the manuscript.

researchers who meet the criteria for access to confidential data.<br><br>*The data underlying the results presented in the study are available from (include the name of the third party and contact information or URL).*<br>• This text is appropriate if the data are owned by a third party and authors do not have permission to share the data.<br><br>* typeset	
Additional data availability information:	

Powered by Editorial Manager® and ProduXion Manager® from Aries Systems Corporation

Manuscript Click here to access/download;Manuscript;Revised_Barriers and Facilitators_Data_Use_Clean.docx

Experiences, Barriers, and Facilitators of Health Data Use among Performance Monitoring Teams (PMT) of Health Facilities in Eastern Ethiopia: A Qualitative Study.

Admas Abera[1]*, Abebe Tolera[1], Biruk Shalmeno[1], Adisu Birhanu Weldesenbet[1], Assefa Tola[1], Tilahun Shiferaw[2], Alemayehu Girma[3], Rania Mohammed[4] and Yadeta Dessie[1]

[1]Epidemiology and Biostatistics Unit, School of Public Health, College of Health and Medical Sciences, Haramaya University, Harar, Ethiopia.

[2]Department of Information Sciences, College of Computing and Informatics, Haramaya University, Haramaya, Ethiopia.

[3]Policy and Plan Directorate, Dire Dawa Administration Health Bureau, Dire Dawa, Ethiopia.

[4]Policy and Plan Directorate, Harari Regional Health Bureau, Harar, Ethiopia.

***Corresponding author:**

Email: admasabera10@gmail.com

Abstract

Background: Using routine health data plays a crucial role in decisions towards improved health outcomes. Despite significant investments in improving Ethiopia's Performance Monitoring Team (PMT), there is limited evidence on the level of involvement, implementation strategies as well as the facilitators and barriers to data utilization by these teams in responding to present and emerging health challenges. This study, therefore, is aimed at exploring PMT experiences, facilitators and barriers to information use in healthcare facilities in Eastern Ethiopia.

Method: This study employed a phenomenological study design**.** Consolidated Framework for Implementation Research (CFIR) framework was adapted to identify the most relevant constructs with the aim of describing data use approaches at six facilities in Dire Dawa and Harari regions in July, 2021. Key informant interview was conducted among 18 purposively selected key informants using a semi-structured interview guide. Thematic coding was applied by using a partially deductive approach, informed by findings from previous studies, and an inductive coding technique with the creation of themes as they emerged from the transcripts. Data were analyzed thematically using ATLAS.ti.

Results: Study participants responded that the main function of PMT is improving service delivery. This study also revealed that data quality, performance, service quality and improvement strategies were among the major focus areas of the PMT. Data use by the PMT was affected by poor quality routine data, lack of accountability, and lack of recognition for good performers. The engagement of PMT members on multiple committees had a negative impact on data use that led to inadequate follow-up of PMT activities, weariness, and lack of time to complete responsibilities.

Conclusion: Performance monitoring teams in health facilities were established and functioning according to the national standard. Barriers to effective data use included engagement of PMT with multiple committees, poor quality of collected data, lack of accountability, and poor documentation practices. Addressing the potential barriers by leveraging the PMT and existing structures have the potential to improve data use and health service performance.

Key words: performance monitoring team, data use, barriers, facilitators, health facilities, Ethiopia.

Introduction

Health systems are complex and continually changing to adapt to changing political, economic, social, technological, and epidemiological realities within constrained resources, particularly in Low- and Middle-Income Countries (LMICs). (1-3) Health facilities need to cope with these changing realities through organized management and leadership, which require use of reliable data for the development of a comprehensive policy package for health sector reforms; improving planning and continuous monitoring. (2, 4)

There has been an increased need for strengthening health service performance to manage population needs through effective leadership (5-7) and improved health facility data use culture. (8-10) The increasing demand for and capacity to use data appears more important than the augmenting supply of evidence (3,13) for improved access to and quality of care. (4,14) Collection, processing, transforming, communicating, and using service delivery reports and administrative records play a crucial role in decisions towards improved health outcomes. (15, 16)

Data use is the process through which decisions-makers and stakeholders explicitly evaluate information in one or more steps of the process of policymaking, program planning and management, or service provision. (6) In the Ethiopian context, the PMT is a team of multidisciplinary health workforce that is primarily responsible for data use. (11, 12) Effective use of the data can enable targeted improvements in health service delivery to meet the population needs. (17)

However, the strength and quality of the evidence needed to get valid information to make decisions about health programs is either weak or insufficient in many developing countries (18-20) mainly due to ineffective data use culture. (20) Inadequate infrastructure, leadership turnover, dysfunctional external relations (26), poor production of information and limited resources (15) were identified as major bottlenecks for information use. In addition to shortage of skilled workforce, imbalances in skill mix, geographical misdistribution, difficulty in inter-professional collaboration, inefficient use of resources, and burnout were found to affect health service quality. (21)

So far, substantial efforts to strengthen health information systems mainly focused on digitization, improving data quality and analysis, and identifying problems, but the ultimate goal is using

information to solve problems, which requires building an information use culture over time. (22) Therefore, engagement (including involvement, commitment, effort or observable behaviour, positive effect or some combination of these) of healthcare leaders and managers is pivotal if we are to improve the Health Information System (HIS) and hence the health service delivery. (4, 13)

The engagement level and capacities needed by health managers and performance monitoring teams to respond to current and emerging issues are not yet well understood. (21) Without radical structural and systemic changes, existing governance structures and management systems will continue to fail to address the existing gaps in health service delivery. (2) Furthermore, despite large investments in performance monitoring team improvement in Ethiopia, a common performance renewal effort is lacking. There is paucity of studies about facilitators and challenges that influence the use of health data to improve primary health care delivery in LMICs. This study, therefore, is aimed at identifying experiences of the PMT, facilitators and barriers to information use process in healthcare facilities in Harari region and Dire Dawa city administration, Eastern Ethiopia.

Methods and Materials

Study Settings

The study was conducted in selected public health facilities of Dire Dawa and Harari regions. Located 515 kilometers from Addis Ababa, Dire Dawa has an estimated population of 341,834, with 68.23% of them living in urban area. Data on public health facilities indicate that, there are about 15 health centers (8 urban and 7 rural), two hospitals, and 32 health posts under the city administration. There are a total of 622 health professionals and 209 health extension workers, and these health facilities serve a total of 480,000 people. Harar is a capital of Harari, one of the regional states of Ethiopia, and is located about 517Km east of Addis Ababa and 48 km south of Dire Dawa. According to the 2007 census, the region has a total population of 183,415 people, of whom 92,316 are men (23) and 54.18% living in urban area. (24) There are three government hospitals, one university teaching hospital, two private hospitals, eight health centers, and 24 health posts in the region.

Study Design

This study used a phenomenological study design. Consolidated Framework for Implementation Research (CFIR) framework to identify the most relevant constructs with the aim of describing data use approaches. The CFIR framework is an evidence-based framework drawing from multiple disciplines that provides a comprehensive arrangement of paradigms influencing complex implementations. The framework has five major domains (Inner setting, Outer setting, Intervention characteristics, Individuals involved and Implementation process) with associated components, all of which were used in this study.

Study Sampling and data collection

We conducted a key informant interview using a semi-structured interview guide. The tool was comprised of questions related to socio-demographic characteristics of the respondents, process of the PMT establishment and implementation strategy, data use processes and practices, barriers to data use, motivating factors for data use, and recommended mechanisms to improve the data use practice.

Two hospitals and four health centers were randomly selected from list of facilities in the two regions. A purposive sampling technique was employed to select the key informants. Eighteen interviews were conducted until data saturation is reached. Data were collected between July 05, 2021 and July 24, 2021 by interviewers fluent in local languages (Amharic and Afan Oromo) and English, with graduate level education and experience in qualitative data collection. All members of the data collection team undergone a one day training workshop to have a common understanding on the interview guide and objectives of the study. The data collection activities were supervised on daily basis by the study investigators. The key informants were PMT members of the health facilities including medical directors of hospitals, heads of health centers, heads of departments in the facilities (outpatient, inpatient, emergency, maternal and child health, pharmacy, laboratory, nursing, human resource and finance team leads), and the Health Management Information System (HMIS) officers. All interviews were conducted in private place suitable for the respondent and the interviews lasted about 20-60 minutes.

Data Analysis

The interviews were transcribed verbatim and field notes were expanded while on the field. The interviews were translated to English and again translated back to the original languages to confirm the accuracy of the meanings. A codebook was developed based on initial review of the transcripts and transcripts were systematically coded using ATLAS.ti software. Double coding was initially used with coding disagreements resolved by discussion and updates to the code definitions where needed. Double coding continued until no new disagreements were identified. Subsequently, summaries for each transcript were written under each code using a matrix. Thematic data analysis was used to describe and compare general statements as relationships and themes and sub-themes present on the data. Accordingly, findings were categorized into five themes. Under each theme, sub-themes were defined and verbatim quotes that represented opinions were applied to substantiate the results. Rigor was enhanced through regular discussions between researchers who read all interview transcripts, counter checked the transcripts, coded the data and agreed on the emerging themes and sub-themes.

Key Themes and Sub-themes

In this study five key themes and about 16 sub-themes have merged. The main themes and sub-themes emerged from the study are presented in Table 1.

Table 1: Categories of Key themes and sub-themes emerged.

**Themes**	**Sub-themes**
Membership and roles of PMT members at the health facilities	• *Roles and responsibilities of PMT members*<br>• *The current PMT status and experiences*
Relevance of PMT strategy and its implementation	• *Perceived benefits of PMT*<br>• *The modality of PMT meetings*<br>• *Adequacy and convenience of time of PMT meeting*<br>• *The focuses of PMT meetings*
Barriers to data use in the facility	• *Organizational barriers*<br>• *Healthcare workers' work ethic and behavioral barriers*<br>• *COVID19 effect on data use*
Motivational mechanisms and demotivating factors in enhancing data use practices in place for PMT members	• *Capacity building practices for PMT members*<br>• *Recognition and non-monetary incentives*<br>• *Demotivating factors*
Recommended mechanisms to improve the data use practices	• *Performance-based recognition*<br>• *Continuous supply of resources, and improving HIS infrastructures*<br>• *Strengthening the overall HIS*<br>• *Enhancing the quality of routine data*

Ethical Considerations

The ethical clearance was obtained from Haramaya University, College of Health and Medical Sciences, Institutional Health Research Ethics Review Committee (IHRERC) with reference number **IHRERC/196/2020**. Permission was sought from all concerned offices, including Dire Dawa Administration and Harari regional health bureaus, and the health facilities. Before data collection, informed written consent was obtained the study participants. Furthermore, participants has been assured of confidentiality of information and their right to withdraw from the study at any time during the study. In this paper, personal identifiers has been omitted to maintain confidentiality, while neutral identifier and age of the participants were mentioned in direct quotes.

Results

Socio-demographic characteristics

A total of 18 in-depth interviews were conducted in two Hospitals and four health centers to explore the PMT experience, barriers and facilitators of data use. Of these participants, 8 were females, 3 were health facility heads, and they have median age (IQR) of 31 (7) years. (Table 2)

Table 2: Socio-demographic characteristics of key informants at the health facilities, Eastern Ethiopia 2021.

Participant ID	Sex	Age in years	Educational status	Work experience in years	Facility type
KI1	Female	34	BSc	11	Health center
KI2	Female	25	Diploma	2	Health center
KI3	Male	27	BSc	9	Health center
KI4	Female	38	BSc	20	Health center
KI5	Male	26	BSc	7	Health center
KI6	Male	34	BSc	13	Health center
KI7	Male	28	Master’s	3	Health center
KI8	Female	25	BSc	1	Health center
KI9	Female	29	BSc	6	Referral Hospital
KI10	Male	28	BSc	32	Referral Hospital
KI11	Male	32	BSc	13	Health center
KI12	Male	30	BSc	7	Health center
KI13	Female	34	BSc	8	Health center
KI14	Female	39	BSc	12	Health center
KI15	Male	42	BSc	19	General Hospital
KI16	Male	35	BSc	3	General Hospital
KI17	Female	35	BSc	12	General Hospital
KI18	Female	30	BSc	10	General Hospital

1. Membership and Roles of PMT at the health facilities

Participants reported that health facilities use certain criteria to select the members of the PMT. The main criteria reported to establish the PMT were being the head of the quality department, HMIS unit team leader, department head, management team, and staff with good performance.

> A respondent stated "*…since the performance of every department is reviewed in the meeting, the main criteria for PMT membership is being a department head. The heads of management unit, HMIS unit and quality department are also included. I became a member because I have served as a vice matron for the last one and half years*." (KI9, 29 years old)
>
> Another key informant pointed out;
>
> "*…any member of the case team can be a member of PMT. It is not necessarily be the case team leader. That means there will be one representative from each case team in PMT in respective of his/her status.*" (KI15, 42 years old)

Roles and responsibilities of PMT members include using health data in improving the health service performance. The specific roles include preparing action plan, putting directions to improve the service delivery, compare performance overtime, monitor monthly performance, provide feedback to various units, building capacity of health workers and preparing reports. The present study also found that the PMT members were aware of their responsibilities.

> One of the participants stated that "*…each member of the PMT is expected to prepare and submit their case team or department reports to the head of the health facility on time. All PMT members will discuss on the submitted report during the monthly PMT meeting in which the reports are crosschecked for its consistency and quality. When the achievements are lower than expected, the reasons for underachievement are sorted out and future directions are put to improve the performance*." (KI4, 38 years old)

The participants' opinions on the alignment of the PMT's roles and responsibilities at the facility with their activities were mixed. While the majority of respondents stated that PMT tasks and responsibilities are totally matched with their actual activities, some stated that they are not completely aligned with their actual activities. There is overlapping of duties and roles, according to these participants.

"As a case team leader, my responsibility is identify the challenges in my activities bur sometimes the roles and activities rendered to us are not related to service performance and members get frustrated due to that" (KI16, 35 years old)

2. Relevance of PMT strategy and its implementation

The perceived benefits of PMT for the facility include improving the service delivery and customer satisfaction. This team is also vital for sharing experiences and skill among the departments and case teams.

> *"… the members of PMT are from different department; there is knowledge sharing on different topics including preparation of reports and how to improve service performance."* (KI11, 32 years old)

Planning, identifying gaps and intervening on the identified problems, and monitoring and evaluating specific activities.

> The key informants further pointed out *"…we select poorly performed activities and the responsible departments will design an action plan for the identified problems. Then the department will be directed to monitor the implementation of the action plan and they are expected to bring the progress in the subsequent meetings."* (KI17, 35 years old)

The modality of PMT meetings. Health facilities have monthly meeting plan in their health facilities which is prepared in advance on the annual plan. Respondents pointed out that the monthly meetings were conducted immediately after each unit submitted their monthly report.

> *"Our PMT meeting is conducted on monthly basis. Immediately after the submission of the monthly report (usually at 21st day of the month), we hold our PMT meeting at 23rd or 24th day of the month. Substitution date will be posted on our telegram page if a meeting is rescheduled."* (KI12, 30 years old)

Adequacy and convenience of time of PMT meeting. On the other hand, it was indicated that the time allocated for PMT meetings was inadequate and inconvenient. All participants unanimously agreed that enough time should be assigned and convenient time should be allocated for PMT me[illegible]gs.

"The meeting time is not enough because every participant usually come to the meeting by interrupting their work. Usually the meeting date and time is decided by the head." (KI16, 35 years old)

Participants discussed how holding the meeting on weekends could reduce absenteeism and unnecessary interruptions.

"…*previously, we used to hold the PMT meeting during working hours and hence the duration of the meetings was not adequate. This was due to high work load of the PMT members. However, currently we are conducting our PMT meeting on Saturdays. Since it is not a working day, PMT members are paid some pocket money for attending the meeting on over the weekend."* (KI8, 25 years old)

The focuses of PMT meetings reported include data quality, performance activities (monthly, quarterly or annual performance), service quality, and the service improvement strategies. According to participants, the first agenda of the PMT meeting is evaluating the previous report, and then comparing it with the current performance. Afterward, the team develops an action plan based on the identified gaps. However, in some health facility the previous performance is not conducted at all.

"*In our monthly PMT meeting, the first thing is presenting the monthly performance report for each unit. Based on the report, gaps and challenges are identified, before action plans are drafted on the identified gaps*". (KI9, 29 years old)

The PMTs mostly utilize routine data for evaluation and monitoring of service delivery programs. There was no culture of using health related data from other sources such as surveys, assessments, research findings.

"*In our health center, the main target of PMT is the routine data. The data accuracy and completeness are checked. Service improvement strategies are also discussed.*" (KI5, 26 years old)

The respondent further stated *"data collected in our health facility is used for making decisions. I have not seen other data sources from researches or surveys being utilized so far."* (KI5, 26 years old)

3. Major barriers to data use in the facility

The organizational barriers to data use at the facility level were poor data quality, being a member of many committees and high patient flow), human resource related issues, input related issues, inadequate budget allocation, as well as lack of performance based incentives.

"…PMT members have their own duties in addition to the PMT which makes it difficult to collect quality data due to competing priorities. If someone is engaged with overlapping duties, the desired outcome may not be achieved". (KI3, 27 years old)

Participants reported that they were in at least two other committees in their health facility. This in turn caused: poor follow up of activities set out by the PMT; being overburdened by committee meetings and assignments. According to the participants, being involved in multiple committees can negatively affect the effectiveness of the PMT activities.

"*…being the member of several committees affects the PMT activities. The aim of including a person in a given team or committee is to perform the task effectively and efficiently in order to bring the intended changes. However, the focus of the individual is usually divided to different committees. In most cases the meeting times is overlapping between different committees"* (KI2, 25 years old)

It was further pointed out:

"Participation in multiple committees decreases the effectiveness of the PMT. Not only for PMT, but even the other committees are also adversely affected. I suggest that don't put the case team leaders on more than one committee." (KI14, 39 years old)

The quality of the routine data collected at health facilities has been reported as a barrier for effective data use.

"There is a gap in data quality including data inconsistency and incompleteness. Healthcare providers perform their daily activity but they do not document it on the register regularly. The data from HMIS may contradict from your observation every day. Hence it is difficult to use our data for decision making due to its poor quality." (KI2, 25 years old)

Another participant further pointed out *"A timely, reliable and high-quality data should be generated in order to use the data for decision making. Monitoring and supporting the staffs is also critical to obtain high quality data."* (KI17, 35 years old)

HIS Input related factors including shortage of patient registration was the other reason mentioned as barrier for data use.

"We have an old register called daily register for documentation of our data. This register was supplied by a Non-Governmental Organization. However, this supply of registers has stopped and currently, we have shortage of the daily registers which affected our documentation." (KI13, 34 years old)

Budget constraint for effective data use at the health facilities was also an influence in the facilities.

"We use the budget which given for the health center, but there is no budget allocated specifically for HMIS." (KI6, 34 years old)

Healthcare workers' work ethic and behavioural barriers were the other sub-theme emerged. Poor commitment and lack of accountability from healthcare providers and PMT members were the most commonly reported challenges. One respondent explained the commitment and competing priorities of PMT members as

"...sometimes we reviewed the same problem repeatedly without a solution. We start to get fed up and start to wonder when it will be solved. Sometimes, we skip the meeting intentionally for this reason. (KI9, 29 years old)

Another respondent pointed out that:

"…health workers that reported incomplete or false data are not given any administrative punishments for their wrong doing." (KI18, 30 years old)

Furthermore, lack of understanding of data value by healthcare workers was indicated as the major challenge faced when using data for action.

"Sometimes the PMT members' attitude towards data is a challenge by itself. The notion that a high quality data can change the hospital is not understood equally among the staffs." (KI9, 29 years old)

According to the participants, most of health professionals have poor understanding and poor attitude on the importance of data in improving quality of health services. Skill gaps in analyzing data using electronic computing software such as District Health Information System version 2 (DHIS2) were also mentioned by the participants.

"Disparity in understanding the importance of the data among our staffs is the other problem. While some people have better understanding towards data, others do not see the importance of it." (KI9, 29 years old)

The respondent further noted;

"There is a problem on registering the data and handling of a registers. Even after the data is entered into DHIS, there is skill gap in analyzing it." (KI10, 32 years old)

Poor tracking of problems, and lack of monitoring of action plans is frequently observed according to the key informants.

"The main problem is that we don't strictly follow the action plans designed in the previous PMT meetings." (KI2, 25 Years old)

Absenteeism and interruption due to competing priorities were also reported during PMT meetings.

"As I said earlier, even if it is a monthly meeting, the date and time should be notified ahead of time. Sudden meetings will affect my routine activities and as a result some

members of the PMT tend to be absent and some interrupt the meeting to go back to their urgent activities." (KI13, 34 years old)

Poor documentation Practice was the other challenge reported by PMT members. It was indicated that in order to change data use culture, the first priority should be changing the attitude of the health care workers before other interventions are introduced.

"*Health professionals should believe documenting data primarily benefits patients, themselves and their health facility. Therefore, working on the attitude change towards documentation should be given more emphasis."* (KI11, 32 years old)

COVID19 effect on data use: COVID-19 pandemic directly and indirectly affected data use practices at the health facilities. An informant explained the effect of COVID-19 pandemic on their day-to-day activity as

"*Due to the occurrence of COVID -19 outbreaks, the nearby hospital was entirely devoted to provide health care service for COVID-19 cases. Hence, all patients in the catchment area of that hospital came to our health center. These conditions increased the work load and compromised our routine activities*." (KI14, 39 years old)

Another key-informant stated **"***...due to COVID-19 the head of HMIS has been in quarantine for the last two months which negatively affected the PMT activities* (KI3, 27 years old)

4. Motivation mechanism in place for PMT members at the facilities

This study identified several motivation mechanisms in place though not across all facilities as well as not conducted in a regular manner. The main motivation strategies reported were capacity building and performance-based recognition.

Capacity building practices for PMT members: According to participants, the main capacity building practices in-service trainings, recognition for good performance, and linked to professional development of the health workers.

"Capacity building on data quality and project development for the members of PMT has been a very good incentive since we never had such experience before." (KI7, 28 years old)

Recognition and Non-monetary incentive mechanisms that can motivate the PMT members in the facility were reported. Participants indicated that these motivational mechanisms become effective if they are directed towards improving the data use culture of the facilities.

A participant pointed out that "*Performance-based recognition and motivation of staffs can improve the data quality and subsequently the data use in the facility."* (KI6, 34 years old)

However, in some health facilities there were no any motivation measures specific to PMT members.

"There are no capacity buildings, recognition mechanisms, and career enhancing educational chance to motivate PMT members." (KI4, 38 years old)

Other participants reported that some sort of recognitions and motivation mechanisms were implemented in their facilities.

"They awarded me this watch for my good performance for properly ensuring the quality of data collected in my health facility." (KI7, 30 years old)

Others believe that incentive may not necessarily motivate staffs to use the data or improve service performance.

"It is hard to say that presence of incentives only positively affects data use. Providing incentives before attitude change may even adversely affect the data use practice." (KI11, 32 years old)

Demotivating factors for effective data use practices were cited by the study participants. The main demotivating factors include shortages of resources, inadequate salary, and inadequate follow up.

“*Salary is one of the demotivating factors because it does not fit with the job we undertake.*” (KI10, 28 years old)

“There is no follow up mechanism after trainings.” (KI8, 25 years old)

The leadership skill of the managers was one of the factors affecting data use in health facilities. Health managers’ and case-team/departments’ poor leadership skills, and lack of value for data were also mentioned as demotivating factors.

“The head of the facility lacks decisiveness and leadership qualities on this job. I don’t think the problem is a gap in knowledge but rather an attitude problem.” (KI13, 34 years old)

Another respondent pointed out “*…if the head of the department or the facility do not utilize data properly, other employees will not care about data use. Hence, the head at district, region or at any other level should lead by example to motivate the other staffs.”* (KI2, 25 years old)

5. Recommended motivation mechanisms for an improved data use

Performance-based recognition was recommended by all informants in this study as major motivation mechanism to improve data use.

“There are those who register and report on time among the health extension workers, the others will be motivated to do the same if you give recognition or incentives for those health workers. The incentive can even be in the form of a certificate.” (KI15, 42 years old)

Another participant stated *“Since the PMT members are the backbone of the health facilities, designing motivating mechanisms is important.”* (KI9, 29 years old)

Continuous supply of resources, improving the infrastructures, and strengthening the HIS.

“*Ensuring continuous supply of necessary materials, and recognizing best performers should be introduced.*” (KI9, 29 years old)

Enhancing the quality of routine data collected was also unanimously believed to enhance data use practice.

> *"In order to use the data for a decision, first I have to receive the data properly. A timely, reliable and complete data should be collected."* (KI1, 34 years old)

Discussion

Adapting the Consolidated Framework for Implementation Research [illegible]IR), this study explored experiences, facilitators and barriers of PMT in the use information for decision at point of healthcare delivery. The findings revealed that PMT in most facilities were established according to the standard criteria for establishment of PMT set by the Ministry of Health (MOH) of Ethiopia. (12) In line with previous studies our study indicated that the primary responsibility of PMT was to improve data quality and use of information to regularly monitor progress and improve health service performance. (11, 12)

Although some irregularities were reported, the monthly meetings were conducted immediately after each unit submitted their monthly report and before submitting their report to the next level to monitor progress and improve performance. The MOH guideline indicates that the meeting dates, venue and its members should be officially communicated in advance and the meeting should be conducted at least a day ahead of submission of the monthly report to the next level. (11, 12, 25)

The present study found that the major focus area of PMT meeting were data quality, performance improvement, and evaluation of previous action plans. Studies on this indicate, follow-up activities for gaps highlighted in the previous PMT meeting should always be the first item on the agenda, followed by an assessment of progress on those gaps. (12, 25) Meetings and collected data have no value in themselves unless action items from meetings are implemented and data are analyzed into meaningful actions. (26)

Health facilities should design strategies to minimize the number of committees and integrate similar committees to improve their service provision. Most PMT members in our study were usually involved in at least two other committees in their health facility. This has its own share of poor follow up of activities set out by the PMT; being overburdened by committee meetings and assignments; creating fatigue and a shortage of time to accomplish PMT assignments.

Evidence indicate data triangulation through the use of other sources such as original research, community feedback, expert opinions, vital registration, censuses and routine HMIS data can yield better results. (12,25,26) Although there were practice of use of routine internal data, information use from external sources was limited.

Previous studies indicated that an organizational context that supports data collection, availability, and use, the technical aspects of data processes and tools, and the behavior of individuals who produce and/or use data are the main elements of health information use. (25, 27) The major challenges of data use reported in the present study emanate from organizational, behavioral and technical sources including poor data quality, competing priorities, shortage of skilled human power, and lack of performance-based motivation for the health workers.

Healthcare organizations are increasingly required to gather and report data about their performance and respond to the results of consequential quality measurements. (28) Excellence in data quality enables healthcare organizations to plan and provide effective and efficient service for users and to meet their targets. (29) Our study revealed that the poor quality of data was one of the major challenges for an informed decision making. A mixed-methods study in Addis Ababa indicated that the PMT meetings that were designed for the sole purpose of improving data quality are not effective. (30) Another qualitative study to explore the facilitators and barriers of digital health technology use also identified data quality as a potential barrier. (31) Lack of available data for several indicators and a lack of validated indicators for important dimensions of quality were identified as major challenges to improve Primary Health Care (PHC) Performance. (6)

Behavioral factors provide crucial insight into the way in which health workers, managers and policymakers use information. (25) The attitude, motivation, and value decision makers attach to information play a big role in determining data use. (2, 25) Concurrent with these, poor commitment, skill gap, lack of accountability for failing to accomplish tasks, work overload, poor

documentation, and resource mismanagement were the main behavioural level challenges reported in this study.

Engagement with different committees increased PMT workload and compromised the effectiveness of data use. Participation in quality improvement collaborative activities may improve health professionals' knowledge, problem-solving skills and attitude; teamwork; shared leadership and habits for improvement. A review of literature indicated shifting roles and expectations in the workplace for health care leadership and management as major challenge for health leadership and workforce. [1] Interaction across quality improvement teams may generate normative pressure and opportunities for capacity building and peer recognition. (11)

Health professionals either do not consider recording and reporting data as part of their routine activities or they just give more priority to the clinical provision and lesser attention to data. (30) Although there is a need to make significant investments in workforce development and training (33), tackling of behavioral factors require interventions that go beyond simple trainings and should focus more on initiatives that enhances positive attitude towards data value. (25)

The individual determinant of motivation among healthcare workers are altruism, attaining professionalism, educational opportunity, and being more experienced. (32) In the present study, it was indicated that health facilities use capacity buildings, and some form of non-monetary incentives to improve performance while most informants indicated dissatisfaction with the absence of such motivating mechanisms. Career development opportunities, in-service trainings, and regular recognition for good performance were also reported to be practiced at the health facilities.

Provision of inputs necessary for HIS such as laptops, internet modems; as well as awarding the best performing health facilities were other motivational methods. Since health professionals can be motivated by a range of extrinsic and intrinsic factors, policy makers need to look beyond traditional financial incentives when designing policies to improve health service performance. (34) Studies indicate training opportunities, transformative leaders, and nonmonetary incentives, staff development and promotion, availability of necessary resources, and ease of communication are found to be effective motivation mechanisms for health care workers. (32, 35)

Conclusion

This study has generated important insights into PMT establishment, its implementation strategy, barriers for data use and recommendations to enhance data use practice at a point of healthcare service delivery. The study found that most performance monitoring teams in health facilities were established and functioning according to the national standard. The study also underscored that barriers to effective data use include engagement of PMT with multiple committees, poor quality of data, lack of accountability, and poor documentation practices. Non-monetary incentives and recognitions for health workers were recommended as means of enhancing data use practice. Improving the quality of routine data, integrating different teams into PMT, establishing accountability framework, and designing documentation methods have the potential to improve informed decision making. While comprehensive countrywide study of the PMT is required, policy makers, stakeholders working in HIS, and health managers should work on improving routine data quality, and design motivational strategies including recognition and non-monetary incentives to improve data use which has the potential to improve health service performance.

Acknowledgements

The authors are thankful to Ministry of Health of Ethiopia, Dire Dawa and Harari regional health bureaus, health facilities, data collectors, and study participants.

Reference

1. Figueroa CA, Harrison R, Chauhan A, Meyer L. Priorities and challenges for health leadership and workforce management globally: a rapid review. *BMC Health Services Research*. 2019;19(1):239.

2. Senkubuge F, Modisenyane M, Bishaw T. Strengthening health systems by health sector reforms. *Global health action*. 2014;7(1):23568.

3. Witter S, Anderson I, Annear P, Awosusi A, Bhandari NN, Brikci N, et al. What, why and how do health systems learn from one another? Insights from eight low- and middle-income country case studies. *Health Research Policy and Systems*. 2019;17(1):9.

4. Nordsteien A, Byström K. Transitions in workplace information practices and culture. *Journal of Documentation*. 2018;74(4):827-43.

5. Veillard J, Cowling K, Bitton A, Ratcliffe H, Kimball M, Barkley S, et al. Better measurement for performance improvement in Low-and Middle-Income countries: the primary health care performance Initiative (PHCPI) experience of conceptual framework development and indicator selection. *The Milbank quarterly*. 2017;95(4):836-83.

6. Tantivess S, Walt G. The role of state and non-state actors in the policy process: the contribution of policy networks to the scale-up of antiretroviral therapy in Thailand. *Health policy and planning*. 2008;23(5):328-38.

7. Fryatt R, Bennett S, Soucat A. Health sector governance: should we be investing more? *BMJ global health*. 2017;2(2):e000343.

8. Lippeveld T. Routine health facility and community information systems: creating an information use culture. Global Health: Science and Practice; 2017. p. 338-40.

9. Nordsteien A, Byström K. Transitions in workplace information practices and culture: The influence of newcomers on information use in healthcare. *Journal of Documentation*. 2018.

10. Jylhä V. Information management in health care: a model for connecting information culture and patient safety. 2017.

11. Adane T, Tadesse T, Endazenaw G. Assessment on Utilization of Health Management Information System at Public Health Centers Addis Ababa City Administrative, Ethiopia. *Internet Things Cloud Comput*. 2017;5(1):7-18.

12. MOH. Information Use Training Module. In: PPMED. Addis Ababa; June 2018.

13. Aqil A, Hozumi D, Lippeveld T. Tools for data demand and use in the health sector: Performance of Routine Information Systems Management (PRISM) Tools. *Measure Evaluation*. 2011.

14. Watson DE, Broemeling A-M, Reid RJ, Black C. A results-based logic model for primary health care : laying an evidence-based foundation to guide performance measurement, monitoring and evaluation. 2004.

15. Endriyas M, Alano A, Mekonnen E, Ayele S, Kelaye T, Shiferaw M, et al. Understanding performance data: health management information system data accuracy in Southern Nations Nationalities and People's Region, Ethiopia. *BMC Health Services Research*. 2019;19(1):175.

16. MOH. Guidance on developing key performance indicators and minimum data sets to monitor healthcare quality. 2013.

17. Rendell N, Lokuge K, Rosewell A, Field E. Factors That Influence Data Use to Improve Health Service Delivery in Low- and Middle-Income Countries. *Global Health: Science and Practice*. 2020;8(3):566.

18. Maphumulo WT, Bhengu BR. Challenges of quality improvement in the healthcare of South Africa post-apartheid: A critical review. *Curationis*. 2019;42(1):1-9.

19. Lee CH, Yoon H-J. Medical big data: promise and challenges. *Kidney research and clinical practice*. 2017;36(1):3.

20. Zamboni K, Baker U, Tyagi M, Schellenberg J, Hill Z, Hanson C. How and under what circumstances do quality improvement collaboratives lead to better outcomes? A systematic review. *Implementation Science*. 2020;15(1):27.

21. West M, Dawson J. Employee engagement and NHS performance: King's Fund London; 2012.

22. Lippeveld T. Routine Health Facility and Community Information Systems: Creating an Information Use Culture. *Global Health: Science and Practice*. 2017;5(3):338.

23. CSA. Population and Housing Census Of Ethiopia. 2007.

24. Commission PC. Summary and statistical report of the 2007 population and housing census. Population size by age and sex. 2008.

25. Mnistry Of Health. Information use participants manual, Training Module. In: Policy Plan, and Evaluation Directorate. Addis Ababa. 2018.

26. Obwocha W, Ayodo G, Nyangura A, Thomas O. Utilization of Healthcare Information Among Healthcare Workers in Gucha Subcounty, Kisii County, Kenya. *J Health Edu Res Dev*. 2016;4(192):2.

27. Vaughn VM, Saint S, Krein SL, Forman JH, Meddings J, Ameling J, et al. Characteristics of healthcare organisations struggling to improve quality: results from a systematic review of qualitative studies. *BMJ Quality & Safety*. 2019;28(1):74.

28. Pine KH, Bossen C, Chen Y, Ellingsen G, Grisot M, Mazmanian M, et al., editors. Data work in healthcare: challenges for patients, clinicians and administrators2018.

29. MOH. Health Data Quality Training Module Participant Manual. In: Policy P, and monitoring and evaluation Directorate editor. Addis Ababa: Federal democratic republic of Ethiopia ministry of health; Noveber 2018.

30. Haftu B, Taye G, Ayele W, Habtamu T, Biruk E. A mixed-methods assessment of routine health information system (RHIS) data quality and factors affecting it, Addis Ababa City administration, Ethiopia, 2020. *Ethiopian Journal of Health Development*. 2021;35(1).

31. Slevin P, Kessie T, Cullen J, Butler MW, Donnelly SC, Caulfield B. Exploring the barriers and facilitators for the use of digital health technologies for the management of COPD: a qualitative study of clinician perceptions. *Qjm*. 2020;113(3):163-72.

32. Muthuri RNDK, Senkubuge F, Hongoro C, editors. Determinants of motivation among healthcare workers in the East African Community between 2009–2019: a systematic review 2020: Multidisciplinary Digital Publishing Institute.

33. Hripcsak G, Bloomrosen M, FlatelyBrennan P, Chute CG, Cimino J, Detmer DE, et al. Health data use, stewardship, and governance: ongoing gaps and challenges: a report from AMIA's 2012 Health Policy Meeting. *Journal of the American Medical Informatics Association*. 2014;21(2):204-11.

34. Lagarde M, Huicho L, Papanicolas I. Motivating provision of high quality care: it is not all about the money. *BMJ*. 2019;366.

35. Weldegebriel Z, Ejigu Y, Weldegebreal F, Woldie M. Motivation of health workers and associated factors in public hospitals of West Amhara, Northwest Ethiopia. *Patient Prefer Adherence*. 2016;10:159-69.

Experience, Barriers, and Facilitators of Data Use among Performance Monitoring Teams (PMT) of Health Facilities in Eastern Ethiopia: *A Qualitative Study*.

Admas Abera[1]*, Abebe Tolera[1], Biruk Shalmeno[1], Adisu Birhanu Weldesenbet[1], Assefa Tola[1], Tilahun Shiferaw[2], Alemayehu Girma[3], Rania Mohammed[4] and Yadeta Dessie[1]

[1]Epidemiology and Biostatistics Unit, School of Public Health, College of Health and Medical Sciences, Haramaya University, Harar, Ethiopia.

[2]Department of Information Sciences, College of Computing and Informatics, Haramaya University, Haramaya, Ethiopia.

[3]Policy and Plan Directorate, Dire Dawa Administration Health Bureau, Dire Dawa, Ethiopia.

[4]Policy and Plan Directorate, Harari Regional Health Bureau, Harar, Ethiopia.

***Corresponding author:**

Email: admasabera10@gmail.com

Abstract

Background: Using routine health data plays a crucial role in decisions towards improved health outcomes. Despite significant investments in improving Ethiopia's Performance Monitoring Team (PMT), there is limited evidence on the level of involvement, implementation strategies as well as the facilitators and barriers to data utilization by these teams in responding to present and emerging health challenges. This study, therefore, is aimed at exploring PMT experiences, facilitators and barriers to information use in healthcare facilities in Eastern Ethiopia.

Method: This study employed a phenomenological study design**.** Consolidated Framework for Implementation Research (CFIR) framework was adapted to identify the most relevant constructs with the aim of describing data use approaches at six facilities in Dire Dawa and Harari regions in July, 2021. Key informant interview was conducted among 18 purposively selected key informants using a semi-structured interview guide. Thematic coding was applied by using a partially deductive approach, informed by findings from previous studies, and an inductive coding technique with the creation of themes as they emerged from the transcripts. Data were analyzed thematically using ATLAS.ti.

Results: Study participants responded that the main function of PMT is improving service delivery. This study also revealed that data quality, performance, service quality and improvement strategies were among the major focus areas of the PMT. Data use by the PMT was affected by poor quality routine data, lack of accountability, and lack of recognition for good performers. The engagement of PMT members on multiple committees had a negative impact on data use that led to inadequate follow-up of PMT activities, weariness, and lack of time to complete responsibilities.

Conclusion: Performance monitoring teams in health facilities were established and functioning according to the national standard. Barriers to effective data use included engagement of PMT with multiple committees, poor quality of collected data, lack of accountability, and poor documentation practices. Addressing the potential barriers by leveraging the PMT and existing structures have the potential to improve data use and health service performance.
Key words: performance monitoring team, data use, barriers, facilitators, health facilities, Ethiopia.

Introduction

Health systems are complex and continually changing to adapt to changing political, economic, social, technological, and epidemiological realities within constrained resources, particularly in Low- and Middle-Income Countries (LMICs). (1-3) Health facilities need to cope with these changing realities through organized management and leadership, which require use of reliable data for the development of a comprehensive policy package for health sector reforms; improving planning and continuous monitoring. (2, 4)

There has been an increased need for strengthening health service performance to manage population needs through effective leadership (5-7) and improved health facility data use culture. (8-10) The increasing demand for and capacity to use data appears more important than the augmenting supply of evidence (3,13) for improved access to and quality of care. (4,14) Collection, processing, transforming, communicating, and using service delivery reports and administrative records play a crucial role in decisions towards improved health outcomes. (15, 16)

Data use is the process through which decisions-makers and stakeholders explicitly evaluate information in one or more steps of the process of policymaking, program planning and management, or service provision. (6) In the Ethiopian context, the PMT is a team of multidisciplinary health workforce that is primarily responsible for data use. (11, 12) Effective use of the data can enable targeted improvements in health service delivery to meet the population needs. (17)

However, the strength and quality of the evidence needed to get valid information to make decisions about health programs is either weak or insufficient in many developing countries (18-20) mainly due to ineffective data use culture. (20) Inadequate infrastructure, leadership turnover, dysfunctional external relations (26), poor production of information and limited resources (15) were identified as major bottlenecks for information use. In addition to shortage of skilled workforce, imbalances in skill mix, geographical misdistribution, difficulty in inter-professional collaboration, inefficient use of resources, and burnout were found to affect health service quality. (21)

So far, substantial efforts to strengthen health information systems mainly focused on digitization, improving data quality and analysis, and identifying problems, but the ultimate goal is using information to solve problems, which requires building an information use culture over time. (22) Therefore, engagement (including involvement, commitment, effort or observable behaviour, positive effect or some combination of these) of healthcare leaders and managers is pivotal if we are to improve the Health Information System (HIS) and hence the health service delivery. (4, 13)

The engagement level and capacities needed by health managers and performance monitoring teams to respond to current and emerging issues are not yet well understood. (21) Without radical structural and systemic changes, existing governance structures and management systems will continue to fail to address the existing gaps in health service delivery. (2) Furthermore, despite large investments in performance monitoring team improvement in Ethiopia, a common performance renewal effort is lacking. There is paucity of studies about facilitators and challenges that influence the use of health data to improve primary health care delivery in LMICs. This study, therefore, is aimed at identifying experiences of the PMT, facilitators and barriers to information use process in healthcare facilities in Harari region and Dire Dawa city administration, Eastern Ethiopia.

Methods and Materials

Study Settings

The study was conducted in selected public health facilities of Dire Dawa and Harari regions. Located 515 kilometers from Addis Ababa, Dire Dawa has an estimated population of 341,834, with 68.23% of them living in urban area. Data on public health facilities indicate that, there are about 15 health centers (8 urban and 7 rural), two hospitals, and 32 health posts under the city administration. There are a total of 622 health professionals and 209 health extension workers, and these health facilities serve a total of 480,000 people. Harar is a capital of Harari, one of the regional states of Ethiopia, and is located about 517Km east of Addis Ababa and 48 km south of Dire Dawa. According to the 2007 census, the region has a total population of 183,415 people, of whom 92,316 are men (23) and 54.18% living in urban area. (24) There are three government

hospitals, one university teaching hospital, two private hospitals, eight health centers, and 24 health posts in the region.

Study Design

This study used a phenomenological study design. Consolidated Framework for Implementation Research (CFIR) framework to identify the most relevant constructs with the aim of describing data use approaches. The CFIR framework is an evidence-based framework drawing from multiple disciplines that provides a comprehensive arrangement of paradigms influencing complex implementations. The framework has five major domains (Inner setting, Outer setting, Intervention characteristics, Individuals involved and Implementation process) with associated components, all of which were used in this study.

Study Sampling and data collection

We conducted a key informant interview using a semi-structured interview guide. The tool was comprised of questions related to socio-demographic characteristics of the respondents, process of the PMT establishment and implementation strategy, data use processes and practices, barriers to data use, motivating factors for data use, and recommended mechanisms to improve the data use practice.

Two hospitals and four health centers were randomly selected from list of facilities in the two regions. A purposive sampling technique was employed to select the key informants. Eighteen interviews were conducted until data saturation is reached. Data were collected between July 05, 2021 and July 24, 2021 by interviewers fluent in local languages (Amharic and Afan Oromo) and English, with graduate level education and experience in qualitative data collection. All members of the data collection team undergone a one day training workshop to have a common understanding on the interview guide and objectives of the study. The data collection activities were supervised on daily basis by the study investigators. The key informants were PMT members of the health facilities including medical directors of hospitals, heads of health centers, heads of departments in the facilities (outpatient, inpatient, emergency, maternal and child health, pharmacy, laboratory, nursing, human resource and finance team leads), and the Health Management Information System (HMIS) officers. All interviews were conducted in private place suitable for the respondent and the interviews lasted about 20-60 minutes.

Data Analysis

The interviews were transcribed verbatim and field notes were expanded while on the field. The interviews were translated to English and again translated back to the original languages to confirm the accuracy of the meanings. A codebook was developed based on initial review of the transcripts and transcripts were systematically coded using ATLAS.ti software. Double coding was initially used with coding disagreements resolved by discussion and updates to the code definitions where needed. Double coding continued until no new disagreements were identified. Subsequently, summaries for each transcript were written under each code using a matrix. Thematic data analysis was used to describe and compare general statements as relationships and themes and sub-themes present on the data. Accordingly, findings were categorized into five themes. Under each theme, sub-themes were defined and verbatim quotes that represented opinions were applied to substantiate the results. Rigor was enhanced through regular discussions between researchers who read all interview transcripts, counter checked the transcripts, coded the data and agreed on the emerging themes and sub-themes.

Ethical Considerations

The ethical clearance was obtained from Haramaya University, College of Health and Medical Sciences, Institutional Health Research Ethics Review Committee (IHRERC) with reference number **IHRERC/196/2020**. Permission was sought from all concerned offices, including Dire Dawa Administration and Harari regional health bureaus, and the health facilities. Before data collection, informed written consent was obtained the study participants. Furthermore, participants has been assured of confidentiality of information and their right to withdraw from the study at any time during the study. In this paper, personal identifiers has been omitted to maintain confidentiality, while neutral identifier and age of the participants were mentioned in direct quotes.

Results

Socio-demographic characteristics

A total of 18 in-depth interviews were conducted in two Hospitals and four health centers to explore the PMT experience, barriers and facilitators of data use. Of these participants, 8 were females, 3 were health facility heads, and they have median age (IQR) of 31 (7) years. (Table 1)

Table 1: Socio-demographic characteristics of key informants at the health facilities, Eastern Ethiopia 2021.

Participant ID	Sex	Age in years	Educational status	Work experience in years	Facility type
KI1	Female	34	BSc	11	Health center
KI2	Female	25	Diploma	2	Health center
KI3	Male	27	BSc	9	Health center
KI4	Female	38	BSc	20	Health center
KI5	Male	26	BSc	7	Health center
KI6	Male	34	BSc	13	Health center
KI7	Male	28	Master's	3	Health center
KI8	Female	25	BSc	1	Health center
KI9	Female	29	BSc	6	Referral Hospital
KI10	Male	28	BSc	32	Referral Hospital
KI11	Male	32	BSc	13	Health center
KI12	Male	30	BSc	7	Health center
KI13	Female	34	BSc	8	Health center
KI14	Female	39	BSc	12	Health center
KI15	Male	42	BSc	19	General Hospital
KI16	Male	35	BSc	3	General Hospital
KI17	Female	35	BSc	12	General Hospital
KI18	Female	30	BSc	10	General Hospital

Key Themes and Sub-themes

In this study five key themes and about 16 sub-themes have merged. The main themes and sub-themes emerged from the study are presented in Table 2.

Table 2: Categories of Key themes and sub-themes emerged.

Formatted: Caption, Keep with next

Themes	Sub-themes
Membership and roles of PMT members at the health facilities	• *Roles and responsibilities of PMT members*<br>• *The current PMT status and experiences*
Relevance of PMT strategy and its implementation	• *Perceived benefits of PMT*<br>• *The modality of PMT meetings*<br>• *Adequacy and convenience of time of PMT meeting*<br>• *The focuses of PMT meetings*
Barriers to data use in the facility	• *Organizational barriers*<br>• *Healthcare workers' work ethic and behavioral barriers*<br>• *COVID19 effect on data use*
Motivational mechanisms and demotivating factors in enhancing data use practices in place for PMT members	• *Capacity building practices for PMT members*<br>• *Recognition and non-monetary incentives*<br>• *Demotivating factors*
Recommended mechanisms to improve the data use practices	• *Performance-based recognition*<br>• *Continuous supply of resources, and improving HIS infrastructures*<br>• *Strengthening the overall HIS*<br>• *Enhancing the quality of routine data*

1. Membership and Roles of PMT at the health facilities

~~In Ethiopia, PMT at health facilities is established based on the standards set by the Ministry of Health. In the present study, p~~Participants reported that health facilities use certain criteria to select the members of the PMT. The main criteria reported to establish the PMT were being the head of the quality department, HMIS unit team leader, department head, management team, and staff with good performance.

A respondent stated "…*since the performance of every department is reviewed in the meeting, the main criteria for PMT membership is being a department head. The heads of management unit, HMIS unit and quality department are also included. I became a member because I have served as a vice matron for the last one and half years*." (KI9, 29 years old)

Another key informant pointed out;

"…*any member of the case team can be a member of PMT. It is not necessarily be the case team leader. That means there will be one representative from each case team in PMT in respective of his/her status."* (KI15, 42 years old)

Roles and responsibilities of PMT members include using health data ~~and~~ in improvinge the health service performance. The specific roles include preparing action plan, putting directions to improve the service delivery, compare performance overtime, monitor monthly performance, provide feedback to various units, building capacity of health workers and preparing reports. The present study also found that the PMT members were aware of their responsibilities.

One of the participants stated that "…*each member of the PMT is expected to prepare and submit their case team or department reports to the head of the health facility on time. All PMT members will discuss on the submitted report during the monthly PMT meeting in which the reports are crosschecked for its consistency and quality. When the achievements are lower than expected, the reasons for underachievement are sorted out and future directions are put to improve the performance*." (~~EPI focal person~~KI4, 38 years old)

The participants' opinions on the alignment of the PMT's roles and responsibilities at the facility with their activities were mixed. While the majority of respondents stated that PMT tasks and responsibilities are totally matched with their actual activities, some stated that they are not completely aligned with their actual activities. There is overlapping of duties and roles, according to these participants.

"As a case team leader, my responsibility is identify the challenges in my activities bur sometimes the roles and activities rendered to us are not related to service performance and members get frustrated due to that" (KI16, 35 years old)

2. Relevance of PMT strategy and its implementation

The perceived benefits of PMT for the facility include improving the service delivery and customer satisfaction. This team is also vital for maintaining the well-functioningsharing experiences and skill among of the departments and case teams. Respondents understood the benefits of PMT for facilities, which include planning, identifying gaps and intervening on the problem, ensuring the facility's progress to success, and monitoring and evaluating program implementations.

Formatted: Font: Not Bold

> *"… the members of PMT are from different department; there is knowledge sharing on different topics including preparation of reports and how to improve service performance."* (KI11, 32 years old)

Formatted: Indent: Left: 0.5", Space After: 8 pt, Add space between paragraphs of the same style

Planning, identifying gaps and intervening on the identified problems, and monitoring and evaluating specific activities.

> The key informants further pointed out "*…we select poorly performed activities and the responsible departments will design an action plan for the identified problems. Then the department will be directed to monitor the implementation of the action plan and they are expected to bring the progress in the subsequent meetings."* (KI17, 35 years old)

Formatted: Indent: Left: 0.5", Space After: 8 pt, Add space between paragraphs of the same style

The modality of PMT meetings.

Adequacy and convenience of time of PMT meeting is essential in order to identify the most important problems in the facility. In this study, tHealth facilities have monthly meeting plan in their health facilities which is prepared in advance on the annual plan. This annual PMT meeting schedule is usually posted in visible area. Moreover, rRespondents pointed out that reported that the monthly meetings were conducted immediately after each unit submitted their monthly report.

Formatted: Font: Not Bold

> *"Our PMT meeting is conducted on monthly basis. Immediately after the submission of the monthly report (usually at 21st day of the month), we hold our PMT meeting at*

2~~3th~~3rd or 24th day of the month. Substitution date will be posted on our telegram page if ~~the~~ a meeting is ~~not conducted based on the~~ rescheduled.” (~~Pharmacy head~~KI12, 30 years old)

Adequacy and convenience of time of PMT meeting.

On the other hand, it was indicated that the time allocated for PMT meetings was inadequate and inconvenient. All participants ~~anonymously~~ unanimously agreed that enough time should be assigned and convenient time should be allocated for PMT meetings.

> *“The meeting time is not enough because every participant usually come to the meeting by interrupting their work. Usually the meeting date and time is decided by the head.”* (KI16, 35 years old)

Formatted: Indent: Left: 0.5"

Participants discussed how holding the meeting on weekends could reduce absenteeism and unnecessary interruptions.

> “…*previously, we used to hold the PMT meeting during working hours and hence the duration of the meetings was not adequate. This was due to high work load of the PMT members. However, currently we are conducting our PMT meeting on Saturdays. Since it is not a working day, ~~we~~ PMT members are paid~~are paying~~ some pocket money for ~~the PMT members~~attending the meeting on over the weekend.”* (~~OPD case team leader~~KI8, 25 years old)

The focuses of PMT meetings reported include data quality, performance activities (monthly, quarterly or annual performance), service quality, and the service improvement strategies. According to participants, the first agenda of the PMT meeting is evaluating the previous report, and then comparing it with the current performance. Afterward, the team develops an action plan based on the identified gaps. However, in some health facility the previous performance is not conducted at all.

> ~~A participant from a hospital stated~~ “*In our monthly PMT meeting, the first thing is presenting the monthly performance report for each unit. Based on the report, gaps and*

challenges are identified, before action plans are drafted on the identified gaps". (KI9Matron Nurse, 29 years old)

The PMTs mostly utilize routine data for evaluation and monitoring of service delivery programs. There was no culture of using health related data from other sources such as surveys, assessments, research findings.

Another participant from a health center reported "*In our health center, the main target of PMT is the routine data. The data accuracy and completeness are checked. Service improvement strategies are also discussed.*" (TB/HIV focal personKI5, 26 years old)

The respondent further stated *"data collected in our health facility is used for making decisions. I have not seen other data sources from researches or surveys being utilized so far."* (TB/HIV focal personKI5, 26 years old)

3. **Effect of engagement in multiple committees in the facility on PMT.** Most participants reported that they involve were in at least two other committees in their health facility. This in turn caused: poor follow up of activities set out by the PMT; being overburdened by committee meetings and assignments; creates fatigue and shortage of time to accomplish PMT assignments. Being involved in multiple committees can negatively affect the effectiveness of the PMT activities.

4. *"…PMT members have their own duties in addition to the PMT which makes it difficult to collect quality data due to competing priorities. If someone is engaged with overlapping duties, the desired outcome may not be achieved".* (Laboratory headKI3, 27 years old)

5. A health center HMIS officer reportedAnother participant reported "*…being the member of several committees affects the PMT activities. The aim of including a person in a given team or committee is to perform the task effectively and efficiently in order to bring the intended changes. However, the focus of the individual is usually divided to different committees. In*

Formatted: Font: Candara

Formatted: Font: Candara

most cases the meeting times is overlapping between different committees" (HMIS officerKI2, 25 years old)

Formatted: Font: Candara

6.3. Major barriers to data use in the facility

Quotes

The barriers PMT members faced when using data for decision making are categorized under the following sub-themes.

Organizational barriers

The organizational barriers to data use at the facility level were poor data quality, being a member of many committees and high patient flow), human resource related issues, (shortage, lack of adequate skills among PMT, etc.), input related issues (ICT, registers, logbooks, HIS guidelines, etc.), infrastructure related issues (inadequate working space), inadequate budget allocation, as well as lack of performance based incentives.

> *"…PMT members have their own duties in addition to the PMT which makes it difficult to collect quality data due to competing priorities. If someone is engaged with overlapping duties, the desired outcome may not be achieved".* (KI3, 27 years old)

, recognition and motivation mechanisms.Participants reported that they were in at least two other committees in their health facility. This in turn caused: poor follow up of activities set out by the PMT; being overburdened by committee meetings and assignments. According to the participants, being involved in multiple committees can negatively affect the effectiveness of the PMT activities.

Formatted: Normal

> A participant reported *"…being the member of several committees affects the PMT activities. The aim of including a person in a given team or committee is to perform the task effectively and efficiently in order to bring the intended changes. However, the focus of the individual is usually divided to different committees. In most cases the meeting times is overlapping between different committees"* (KI2, 25 years old)

Formatted: Indent: Left: 0.5"

It was further pointed out:

"Participation in multiple committees decreases the effectiveness of the PMT. Not only for PMT, but even the other committees are also adversely affected. I suggest that don't put the case team leaders on more than one committee." (KI14, 39 years old)

Formatted: Font: Not Bold, Font color: Auto

Formatted: Indent: Left: 0.5"

Formatted: Font: Not Bold, Font color: Auto

The quality of the routine data collected at health facilities has been reported as a barrier for effective data use.

> *"There is a gap in data quality including data inconsistency and incompleteness. Healthcare providers perform their daily activity but they do not document it on the register regularly. The data from HMIS may contradict from your observation every day. Hence it is difficult to use our data for decision making due to its poor quality."* (~~HMIS officer~~KI2, 25 years old)

> Another participant further pointed out *"A timely, reliable and high-quality data should be generated in order to use the data for decision making. Monitoring and supporting the staffs is also critical to obtain high quality data."* (~~MCH department head~~KI17, 35 years old)

HIS Input related factors including shortage of patient registration was the other reason mentioned as barrier for data use.

Formatted: Font: Not Bold

Formatted: Font: Not Bold

> ~~Another participant supervisor of health extension workers stated~~ *"~~we~~We have an old register called daily register for documentation of our data. This register was supplied by ~~the~~ a ~~NG~~Non-Governmental Organization. However, ~~currently~~ this ~~funding~~ supply of registers has stopped and currently, we ~~faced~~ have shortage of the daily registers which ~~. This shortage~~ affected our documentation."* (~~HEW supervisor~~KI13, 34 years old)

Budget constraint for effective data use at the health facilities was also an influence in the facilities.

> *"We use the budget which given for the health center, but there is no budget allocated specifically for HMIS."* (KI6, 34 years old)

Incentives, Recognitions and motivational mechanism in the health facility can promote motivate employees to strive for better performance. Absence of performance-based incentives, recognition and motivation mechanisms was one of were among the main organizational challenges mentioned by participants.

Quotes

If the health facility designs performance-based recognition and motivational programs related to data use, it has the potential to promote motivate others to improve their data use culture. While participants reported that some sort of recognitions and motivation mechanisms were implemented in their facilities, others believe that incentive by themselves may not necessarily motivate staffs to use the data or improve service performance.

Quotes

Healthcare workers' behavioral barrierswork ethic and behavioural barriers were the other sub-theme emerged.

Poor commitment and lack of accountability from healthcare providers and PMT members were the most commonly reported challenges. One respondent from hospital explained the commitment and competing priorities of PMT members as

> *"…sometimes we reviewed the same problem repeatedly without a solution. We start to get fed up and start to wonder when it will be solved. Sometimes, we skip the meeting intentionally for this reason.* (Matron NurseKI9, 29 years old)

Another HMIS officer at a hospital saidrespondent pointed out that:;

> *"…health workers that reported incomplete or false data are not given any administrative punishments for their wrong doing.*" (HMIS officerKI18, 29 30 years old)

Formatted: Font: Bold

Formatted: Indent: Left: 0.5"

Formatted: Font: Bold, Italic, Font color: Blue

Formatted: Space Before: 0 pt, After: 10 pt

Formatted: Space Before: 0 pt, After: 10 pt, Adjust space between Latin and Asian text, Adjust space between Asian text and numbers

Formatted: Normal, Space Before: 0 pt

Formatted: Font: Not Bold

Formatted: Font: Not Bold

Formatted: Font: Times New Roman, 12 pt

Formatted: Space Before: 0 pt, After: 10 pt

Furthermore, lack of understanding of data value by healthcare workers was indicated as the major challenge faced when using data for action.

> *"Sometimes the PMT members' attitude towards data is a challenge by itself. The notion that a high quality data can change the hospital is not understood equally among the staffs."* (KI9, 29 years old)

According to the participants, most of health professionals have poor understanding and poor attitude on the importance of data in improving quality of health services. Skill gaps in analyzing data using electronic computing software such as District Health Information System version 2 (DHIS2) were also mentioned by the participants.

> *"Disparity in understanding the importance of the data among our staffs is the other problem. While some people have better understanding towards data, others do not see the importance of it."* (KI9, 29 years old)

Formatted: Font: Not Bold, Font color: Auto

Formatted: Indent: Left: 0.5"

Formatted: Font: Not Bold, Font color: Auto

The respondent further noted;

Formatted: Font: Not Bold, Not Italic, Font color: Auto

> *"There is a problem on registering the data and handling of a registers. Even after the data is entered into DHIS, there is skill gap in analyzing it."* (KI10, 32 years old)

Formatted: Font: Not Bold, Font color: Auto

Formatted: Indent: Left: 0.5"

Formatted: Font: Not Bold, Font color: Auto

Poor tracking of problems, and lack of monitoring of action plans is frequently observed according to the key informants.

> *"The main problem is that we don't strictly follow the action plans designed in the previous PMT meetings."* (KI2, 25 Years old)

Absenteeism and interruption due to competing priorities were also reported during PMT meetings.

> *"As I said earlier, even if it is a monthly meeting, the date and time should be notified ahead of time. Sudden meetings will affect my routine activities and as a result some members of the PMT tend to be absent and some interrupt the meeting to go back to their urgent activities."* (KI13, 34 years old)~~Quotes~~

Poor documentation Practice was the other challenge reported by PMT members. ~~Some staffs do not properly record the service provided on the registries~~registers~~. Staff attitude towards data was also mentioned as a challenge for evidence based decision making. In addition, some staffs carry out their routine activities such as registering and reporting carelessly.~~

Formatted: Font: Not Bold

~~Quotes~~

Formatted: Highlight

~~The participants~~ It was ~~suggested~~ indicated that in order to change data use culture, the first priority should be changing the attitude of the health care workers before other interventions are introduced.

> "*Health professionals should believe documenting data primarily benefits patients, themselves and their health facility. Therefore, working on the attitude change towards documentation should be given more emphasis.*" (~~Health center head~~KI11, 32 years old)

COVID19 effect on data use: COVID-19 pandemic directly and indirectly affected data use practices at the health facilities. An informant explained the effect of COVID-19 pandemic on their day-to-day activity as

> "*Due to the occurrence of COVID -19 outbreaks, the nearby hospital was entirely devoted to provide health care service for COVID-19 cases. Hence, all patients in the catchment area of that hospital came to our health center. These conditions increased the work load and compromised our routine activities.*" (~~EPI focal person~~KI14, 39 years old)

Formatted: Font: Candara

> Another key-informant stated "*…due to COVID-19 the head of HMIS has been in quarantine for the last two months which negatively affected the PMT activities* (KI3, 27 years old)

Formatted: List Paragraph, Adjust space between Latin and Asian text, Adjust space between Asian text and numbers

Formatted: Not Highlight

~~7.~~4. Motivation mechanism in place for PMT members at the facilities

This study identified several motivation mechanisms in place though not ~~all~~ across all facilities as well as not conducted in a regular manner. The main motivation strategies reported were capacity building and performance-based recognition.

Capacity building practices for PMT members: According to participants, the main capacity building practices ~~for PMT members were career development opportunities,~~ in-service trainings, recognition for good performance, ~~appropriate selection of training participants, high quality of trainings, post training follow up,~~ and linked to professional development of the health workers.

> *"Capacity building on data quality and project development for the members of PMT has been a very good incentive since we never had such experience before."* (KI7, 28 years old)

Recognition and ~~Recognition and n~~Non-monetary incentive ~~s~~ ~~. The participants listed several non-monetary incentive~~ mechanisms that can motivate the PMT members in the facility were reported. ~~The main non-monetary~~ incentives ~~a~~were ~~provision of mobile cards, and recognition of good performers, as well as awarding the~~ of best performing ~~facilities based by regional health bureau.~~ Participants ~~also~~ indicated that these motivational mechanisms become effective ~~only as far as the information~~ if they are directed towards improving the data use culture of the facilities ~~is improved~~.

> A participant ~~suggested~~ pointed out that "*Performance-based recognition and motivation of staffs can improve the data quality and subsequently the data use in the facility.*" (~~Health Center head~~KI6, 34 years old)

However, in some health facilities there were no any motivation measures specific to PMT members.

> *"There are no capacity buildings, recognition mechanisms, and career enhancing educational chance to motivate PMT members."* (~~TB/HIV focal person~~KI4, 38 years old)

Formatted: Font: Not Bold

Formatted: Font: Bold

Other participants reported that some sort of recognitions and motivation mechanisms were implemented in their facilities.

> *"They awarded me this watch for my good performance for properly ensuring the quality of data collected in my health facility."* (KI7, 30 years old)

Others believe that incentive may not necessarily motivate staffs to use the data or improve service performance.

> *"It is hard to say that presence of incentives only positively affects data use. Providing incentives before attitude change may even adversely affect the data use practice."* (KI11, 32 years old)

Demotivating factors for effective data use practices were cited by the study participants. The main demotivating factors include shortages of resources~~, poor quality of routine data, insufficient human power, and lack of accountability~~, inadequate salary, and inadequate follow up.~~ Some study participants mentioned the follow up from Woreda or regional health bureau was not adequate.~~

> "*~~salary~~Salary is one of the demotivating factors because it does not fit with the job we undertake.*" (~~NCD focal person~~KI10, 28 years old)

> *"There is no follow up mechanism after trainings."* (KI8, 25 years old)

The leadership skill of the managers was one of the factors ~~to promote the~~~~affecting~~affecting data use in health facilities. Health managers' and case-team/departments' poor leadership skills, and lack of value for data were also mentioned as demotivating factors.

> *"The head of the facility lacks decisiveness and leadership qualities on this job. I don't think the problem is a gap in knowledge but rather an attitude problem."* (KI13, 34 years old)

> Another respondent indicated "…*if the head of the department or the facility do not utilize data properly, other employees will not care about data use. Hence, the head at ~~woreda~~ district, ~~or~~ region or at any other level should ~~be~~ lead by example ~~for~~to motivate*

Formatted: Font: Italic

Formatted: Not Highlight

Formatted: Indent: Left: 0.5"

the other staffs in order to improve the data use culture.” (HMIS officerKI2, 25 years old)

5. Recommended motivation mechanisms for an improved data use

Performance-based recognition was recommended by all informants in this study as major motivation mechanism including p to improve data use., arranging meeting sites and time,

“There are those who register and report on time among the health extension workers, the others will be motivated to do the same if you give recognition or incentives for those health workers. The incentive can even be in the form of a certificate.” (KI15, 42 years old)

Another participant stated *“Since the PMT members are the backbone of the health facilities, designing motivating mechanisms is important.”* (KI9, 29 years old)

capacity building, cContinuous supply of resources, improving the infrastructures, and strengthening the HIS.

“*Ensuring continuous supply of necessary materials, and recognizing best performers should be introduced.*” (Matron NurseKI9, 29 years old)

Enhancing the quality of routine data collected was also unanimously believed to enhance data use practice.

“In order to use the data for a decision, first I have to receive the data properly. A timely, reliable and complete data should be collected.” (KI1, 34 years old)

Formatted: Indent: Left: 0"

Formatted: Indent: Left: 0.5"

Formatted: Line spacing: Multiple 1.15 li

Discussion

Adapting the Consolidated Framework for Implementation Research (CFIR), this study explored experiences, facilitators and barriers of PMT in the use information for decision at point of healthcare delivery. The findings revealed that PMT in most facilities were established according to the standard criteria for establishment of PMT set by the Ministry of Health (MOH) of Ethiopia. (12) In line with previous studies our study indicated that the primary responsibility of PMT was to improve data quality and use of information to regularly monitor progress and improve health service performance. (11, 12)

Formatted: Not Superscript/ Subscript

Formatted: Not Superscript/ Subscript

~~In line with the national guideline, a~~Although some irregularities were reported, the monthly meetings were conducted ~~monthly~~ immediately after each unit submitted their monthly report and before submitting their report to the next level to monitor progress and improve performance. The MOH guideline indicates that the meeting dates, venue and its members should be officially communicated in advance and the meeting should be conducted at least a day ahead of submission of the monthly report to the next level. (11, 12, 25)

Formatted: Not Superscript/ Subscript

The present study found that the major focus area of PMT meeting were data quality, performance improvement, and evaluation of previous action plans. Studies on this indicate, follow-up activities for gaps highlighted in the previous PMT meeting should always be the first item on the agenda, followed by an assessment of progress on those gaps. (12, 25) Meetings and collected data have no value in themselves unless action items from meetings are implemented and data are analyzed into meaningful actions. (26)

Formatted: Not Superscript/ Subscript

Formatted: Not Superscript/ Subscript

Health facilities should design strategies to minimize the number of committees and integrate similar committees to improve their service provision. Most PMT members in our study were usually involved in at least two other committees in their health facility. This has its own share of poor follow up of activities set out by the PMT; being overburdened by committee meetings and assignments; creating fatigue and a shortage of time to accomplish PMT assignments.

Evidence indicate data triangulation through the use of other sources such as original research, community feedback, expert opinions, vital registration, censuses and routine HMIS data can yield better results. (12,25,26) Although there were practice of use of routine internal data, information use from external sources was limited.

Formatted: Not Superscript/ Subscript

Formatted: Not Superscript/ Subscript

Previous studies indicated that an organizational context that supports data collection, availability, and use, the technical aspects of data processes and tools, and the behavior of individuals who produce and/or use data are the main elements of health information use. (25, 27) The major challenges of data use reported in the present study emanate from organizational, behavioral and technical sources including poor data quality, competing priorities, shortage of skilled human power, and lack of performance-based motivation for the health workers.

Formatted: Not Superscript/ Subscript

Healthcare organizations are increasingly required to gather and report data about their performance and respond to the results of consequential quality measurements. (28) Excellence in data quality enables healthcare organizations to plan and provide effective and efficient service for users and to meet their targets. (29) Our study revealed that the poor quality of data was one of the major challenges for an informed decision making. A mixed-methods study in Addis Ababa indicated that the PMT meetings that were designed for the sole purpose of improving data quality are not effective. (30) Another qualitative study to explore the facilitators and barriers of digital health technology use also identified data quality as a potential barrier. (31) Lack of available data for several indicators and a lack of validated indicators for important dimensions of quality were identified as major challenges to improve Primary Health Care (PHC) Performance. (6)

Formatted: Not Superscript/ Subscript

Formatted: Not Superscript/ Subscript

Formatted: Not Superscript/ Subscript

Formatted: Not Superscript/ Subscript

Formatted: Not Superscript/ Subscript

Behavioral factors provide crucial insight into the way in which health workers, managers and policymakers use information. (25) The attitude, motivation, and value decision makers attach to information play a big role in determining data use. (2, 25) Concurrent with these, poor commitment, skill gap, lack of accountability for failing to accomplish tasks, work overload, poor documentation, and resource mismanagement were the main behavioural level challenges reported in this study.

Formatted: Not Superscript/ Subscript

Formatted: Not Superscript/ Subscript

Engagement with different committees increased PMT workload and compromised the effectiveness of data use. Participation in quality improvement collaborative activities may improve health professionals' knowledge, problem-solving skills and attitude; teamwork; shared leadership and habits for improvement. A review of literature indicated shifting roles and expectations in the workplace for health care leadership and management as major challenge for

health leadership and workforce. [1] Interaction across quality improvement teams may generate normative pressure and opportunities for capacity building and peer recognition. (11)

Health professionals either do not consider recording and reporting data as part of their routine activities or they just give more priority to the clinical provision and lesser attention to data. (30) Although there is a need to make significant investments in workforce development and training (33), tackling of behavioral factors require interventions that go beyond simple trainings and should focus more on initiatives that enhances positive attitude towards data value. (25)

The individual determinant of motivation among healthcare workers are altruism, attaining professionalism, educational opportunity, and being more experienced. (32) In the present study, it was indicated that health facilities use capacity buildings, and some form of non-monetary incentives to improve performance while most informants indicated dissatisfaction with the absence of such motivating mechanisms. Career development opportunities, in-service trainings, and regular recognition for good performance were also reported to be practiced at the health facilities.

Provision of inputs necessary for HIS such as laptops, internet modems; as well as awarding the best performing health facilities were other motivational methods. Since health professionals can be motivated by a range of extrinsic and intrinsic factors, policy makers need to look beyond traditional financial incentives when designing policies to improve health service performance. (34) Studies indicate training opportunities, transformative leaders, and nonmonetary incentives, staff development and promotion, availability of necessary resources, and ease of communication are found to be effective motivation mechanisms for health care workers. (32, 35)

Conclusion

This study has generated important insights into PMT establishment, its implementation strategy, barriers for data use and recommendations to enhance data use practice at a point of healthcare

Formatted: Not Superscript/ Subscript

Formatted: Not Superscript/ Subscript

Formatted: Not Superscript/ Subscript

Formatted: Not Superscript/ Subscript

Formatted: Font color: Auto

Formatted: Font color: Black, Not Superscript/ Subscript

Formatted: Font color: Black

Formatted: Not Superscript/ Subscript

service delivery. The study found that most performance monitoring teams in health facilities were established and functioning according to the national standard. The study also underscored that barriers to effective data use include engagement of PMT with multiple committees, poor quality of data, lack of accountability, and poor documentation practices. Non-monetary incentives and recognitions for health workers were recommended as means of enhancing data use practice. Improving the quality of routine data, integrating different teams into PMT, establishing accountability framework, and designing documentation methods have the potential to improve informed decision making. While comprehensive countrywide study of the PMT is required, policy makers, stakeholders working in HIS, and health managers should work on improving routine data quality, and design motivational strategies including recognition and non-monetary incentives to improve data use which has the potential to improve health service performance.

Acknowledgements

The authors are thankful to Ministry of Health of Ethiopia, Dire Dawa and Harari regional health bureaus, health facilities, data collectors, and study participants.

Reference

1. Figueroa CA, Harrison R, Chauhan A, Meyer L. Priorities and challenges for health leadership and workforce management globally: a rapid review. *BMC Health Services Research*. 2019;19(1):239.

2. Senkubuge F, Modisenyane M, Bishaw T. Strengthening health systems by health sector reforms. *Global health action*. 2014;7(1):23568.

3. Witter S, Anderson I, Annear P, Awosusi A, Bhandari NN, Brikci N, et al. What, why and how do health systems learn from one another? Insights from eight low- and middle-income country case studies. *Health Research Policy and Systems*. 2019;17(1):9.

4. Nordsteien A, Byström K. Transitions in workplace information practices and culture. *Journal of Documentation*. 2018;74(4):827-43.

5. Veillard J, Cowling K, Bitton A, Ratcliffe H, Kimball M, Barkley S, et al. Better measurement for performance improvement in Low-and Middle-Income countries: the primary health care performance Initiative (PHCPI) experience of conceptual framework development and indicator selection. *The Milbank quarterly*. 2017;95(4):836-83.

6. Tantivess S, Walt G. The role of state and non-state actors in the policy process: the contribution of policy networks to the scale-up of antiretroviral therapy in Thailand. *Health policy and planning*. 2008;23(5):328-38.

7. Fryatt R, Bennett S, Soucat A. Health sector governance: should we be investing more? *BMJ global health*. 2017;2(2):e000343.

8. Lippeveld T. Routine health facility and community information systems: creating an information use culture. Global Health: Science and Practice; 2017. p. 338-40.

9. Nordsteien A, Byström K. Transitions in workplace information practices and culture: The influence of newcomers on information use in healthcare. *Journal of Documentation*. 2018.

10. Jylhä V. Information management in health care: a model for connecting information culture and patient safety. 2017.

11. Adane T, Tadesse T, Endazenaw G. Assessment on Utilization of Health Management Information System at Public Health Centers Addis Ababa City Administrative, Ethiopia. *Internet Things Cloud Comput*. 2017;5(1):7-18.

12. MOH. Information Use Training Module. In: PPMED. Addis Ababa; June 2018.

13. Aqil A, Hozumi D, Lippeveld T. Tools for data demand and use in the health sector: Performance of Routine Information Systems Management (PRISM) Tools. *Measure Evaluation*. 2011.

14. Watson DE, Broemeling A-M, Reid RJ, Black C. A results-based logic model for primary health care : laying an evidence-based foundation to guide performance measurement, monitoring and evaluation. 2004.

15. Endriyas M, Alano A, Mekonnen E, Ayele S, Kelaye T, Shiferaw M, et al. Understanding performance data: health management information system data accuracy in Southern Nations Nationalities and People's Region, Ethiopia. *BMC Health Services Research*. 2019;19(1):175.

16. MOH. Guidance on developing key performance indicators and minimum data sets to monitor healthcare quality. 2013.

17. Rendell N, Lokuge K, Rosewell A, Field E. Factors That Influence Data Use to Improve Health Service Delivery in Low- and Middle-Income Countries. *Global Health: Science and Practice*. 2020;8(3):566.

18. Maphumulo WT, Bhengu BR. Challenges of quality improvement in the healthcare of South Africa post-apartheid: A critical review. *Curationis*. 2019;42(1):1-9.

19. Lee CH, Yoon H-J. Medical big data: promise and challenges. *Kidney research and clinical practice*. 2017;36(1):3.

20. Zamboni K, Baker U, Tyagi M, Schellenberg J, Hill Z, Hanson C. How and under what circumstances do quality improvement collaboratives lead to better outcomes? A systematic review. *Implementation Science*. 2020;15(1):27.

21. West M, Dawson J. Employee engagement and NHS performance: King's Fund London; 2012.

22. Lippeveld T. Routine Health Facility and Community Information Systems: Creating an Information Use Culture. *Global Health: Science and Practice*. 2017;5(3):338.

23. CSA. Population aAnd Housing Census Of Ethiopia. 2007.

24. Commission PC. Summary and statistical report of the 2007 population and housing census. Population size by age and sex. 2008.

25. Mnistry Of Health. Information use participants manual, Training Module. In: Policy Plan, and Evaluation Directorate. Addis Ababa. 2018.

26. Obwocha W, Ayodo G, Nyangura A, Thomas O. Utilization of Healthcare Information Among Healthcare Workers in Gucha Subcounty, Kisii County, Kenya. *J Health Edu Res Dev*. 2016;4(192):2.

27. Vaughn VM, Saint S, Krein SL, Forman JH, Meddings J, Ameling J, et al. Characteristics of healthcare organisations struggling to improve quality: results from a systematic review of qualitative studies. *BMJ Quality & Safety*. 2019;28(1):74.

28. Pine KH, Bossen C, Chen Y, Ellingsen G, Grisot M, Mazmanian M, et al., editors. Data work in healthcare: challenges for patients, clinicians and administrators2018.

29. MOH. Health Data Quality Training Module Participant Manual. In: Policy P, and monitoring and evaluation Directorate editor. Addis Ababa: Federal democratic republic of Ethiopia ministry of health; Noveber 2018.

30. Haftu B, Taye G, Ayele W, Habtamu T, Biruk E. A mixed-methods assessment of routine health information system (RHIS) data quality and factors affecting it, Addis Ababa City administration, Ethiopia, 2020. *Ethiopian Journal of Health Development*. 2021;35(1).

31. Slevin P, Kessie T, Cullen J, Butler MW, Donnelly SC, Caulfield B. Exploring the barriers and facilitators for the use of digital health technologies for the management of COPD: a qualitative study of clinician perceptions. *Qjm*. 2020;113(3):163-72.

32. Muthuri RNDK, Senkubuge F, Hongoro C, editors. Determinants of motivation among healthcare workers in the East African Community between 2009–2019: a systematic review 2020: Multidisciplinary Digital Publishing Institute.

33. Hripcsak G, Bloomrosen M, FlatelyBrennan P, Chute CG, Cimino J, Detmer DE, et al. Health data use, stewardship, and governance: ongoing gaps and challenges: a report from AMIA's 2012 Health Policy Meeting. *Journal of the American Medical Informatics Association*. 2014;21(2):204-11.

34. Lagarde M, Huicho L, Papanicolas I. Motivating provision of high quality care: it is not all about the money. *BMJ*. 2019;366.

35. Weldegebriel Z, Ejigu Y, Weldegebreal F, Woldie M. Motivation of health workers and associated factors in public hospitals of West Amhara, Northwest Ethiopia. *Patient Prefer Adherence*. 2016;10:159-69.

Section	Reviewer's comment	Authors' responses
**General**		The authors are really thankful for the insightful comments and feedback from the reviewer to improve our paper. We have critically looked at all the reviewer's comments and addressed all of them in detail. The addressed parts are explained below on a point-by-point response
**Abstract**	Typo errors suggested	All typos have been corrected which can be seen in the revised manuscript.
**Introduction**	Typo errors suggested	All typos have been corrected which can be seen in the revised manuscript.
**Methods and Materials**	The sentence is repetition of the last sentence in a paragraph above. You may consider choosing which to keep (page 5, last paragraph)	The repeated sentence has been removed to make the writing brief.
	This covers forward translation. What about the backward translation to confirm accuracy of the translation? (page 6, first paragraph)	Additional sentence indicating the backward translation is included in the revised version. We have conducted the translation but we failed to include it in the initial version. Thank you for pointing this out.
	Can you further indicate on how trustworthiness was ensured (page 6, first paragraph)	How the rigor and trustworthiness of the study was ensured has been explained under data analysis section.
**Results**	A fifth theme may also be necessary. This should be on relevance of PMT strategy and its implementation. Some sub-themes listed under key theme 1 do not necessarily blend well to the theme title	We found this comment useful and a fifth theme has been included as per your suggestion. The authors would like to thank you on this.
	A fifth theme may also be necessary. This should be on relevance of PMT strategy and its implementation. Some sub-themes listed under key theme 1 do not necessarily blend well to the theme title (page 8, first paragraph)	A fifth theme has been added by classifying the first theme into *membership and roles of PMT at health facilities* and *relevance of PMT strategy and its implementation. Many thanks for the suggestion on this.*
	Given the sample size for the study and generally qualitative studies, this identifier is likely to compromise anonymity. Please consider using other more neutral identifier like participants ID. With other facilities, it happens that there is only one program planning team leader and subsequently one could easily identify who said what. (page 8, second qoute paragraph)	Dear reviewer, I think this is an important issues to note. We agree on this point on ensuring the ethical issues and confidentiality of the participants. To remedy this, we have removed the working position of the key informants and replaced it with neutral identification (KI1, KI2, etc.) which stand for Key Informant 1, 2,…. And age of the respondent in the direct quotes.

	It is not clear why this is in bold letters. By the look, it seems as a sub-theme. However, there was no indication in the data analysis section that there were also such. Please synchronise	Again, this comment is valid and accepted. To make sure the writing is consistent, we have clearly indicated the themes and sub-themes in Table 2. All the sub-themes are written in bold letters in the revised manuscript to make the writing clearer for readers and the themes are presented as a heading. Thank you for the feedback.
	There is no data from participants presented under this section. (under roles and responsibilities of PMT result) (page 9, first paragraph)	The reviewer have raised a number of important feedback on lack of direct quotes from respondents. This comment has been accepted. Throughout the entire paper, we have tried to make significant revisions by adding more direct quotes from key informants. Please look at the revisions made for the whole results.
	I suggest you also indicate under methods for data collection sub-themes that emerged (page 9, fourth paragraph)	All the themes and sub-themes emerged has been presented clearly in Table 1 under the methods section in the revised version. Kindly check table 1.
	Please confirm if:<br>1. PMT members are paying for attending the meeting on Saturdays, or<br>2. PMT member are paid and get pocket money for attending meeting over weekend.<br><br>As it stands, this sentence of the transcription is confusing. (page 10, fourth paragraph)	This sentences has been revised to make the meaning clearer. The PMT members were paid by the health facility when they attend the meeting on weekends. Please check the revised version under adequacy and convenience of time of PMT meeting sub-theme.
	It becomes more rich when participants quotations are provided to show what they reported above what the author will present as having been reported. In this paragraph, and several others, authors do not show what participants reported yet there are strong findings which one would wish to verify and compare to the presentation. (page 11, 3rd paragraph)	This well accepted. Throughout the entire paper, we have tried to make significant revisions by adding more direct quotes from key informants. Please look at the revisions made for the whole results. Almost all results have been supported by the direct quotes from key informants in the revised version.
	Effect of multiple committees can be better positioned under barriers. Esp under organizational (Page 11, 4th paragraph)	The effect of multiple committee on data use has been moved to organizational barrier.
	It is not clear whether this paragraph discusses or introduces views of participants. If it introduces reported	It introduces the reported barriers and as explained above, we have now revised this and added direct quotes in the writings.

	organisational barriers, as it should, please also put direct quotes from participants indicating these barriers. Moving effects to this position may remedy this. (Page 12, 2nd paragraph)	
	Incentives and recognition results are motivating factors and might not jell with barriers. Please move to motivational mechanisms below (Page 13, 2nd paragraph)	This is accepted and moved motivational mechanisms sub-theme.
	Health workers' work ethics might be more relevant term than health workers behavioural factors (Page 13, 3rd paragraph)	Thank you for the feedback on this. We believe some of the factors initially were under organizational barriers (such as attitude, skill gap and knowledge) move beyond work ethic. However, commitment, poor documentation practice are indeed work ethics issues. To accommodate these concepts, we have revised the sub-theme as "health workers work ethics and behavioural barriers" We hope this can cover all the concepts raised by the key informants.
	This is a key theme Please reconsider titling/heading of your themes and sub-themes for consistency (Page 16, 1st paragraph)	Accepted and revised.
Discussion	A section under findings, which did not reflect on actual data from participants, suggested that participants reported some form of incentives. This is not precise reflection of findings. The same section reflected dissatisfaction about the lack of these mechanisms. Please confirm and create synergy	Dear reviewer, thank you for the insightful comment on this. We have revised this so that it directly reflects the finding of the study. The discussion part is revised to make it consistent with the result.
	This has not transpired under findings. Please discuss your results OR confirm this in your results. (first sentence of the last paragraph on page 20)	This sentence was not the finding of the study rather we were discussing finding from other literature. It was not clear and has been revised in the revised manuscript. In addition, the references that were not in the list were omitted. It was a typo.
**Grammatical errors and typos in the entire paper**		**We are really grateful for reviewing the manuscript in detail looking for typos and grammatical errors. All of these have been accepted and corrected per your comment in the revised paper.**
