# Supplementary material for: Experiences, barriers, and facilitators of health data use among performance monitoring teams (PMT) of health facilities in Eastern Ethiopia: A qualitative study
Source: PLoS One. 2023 May 11;18(5):e0285662. doi: 10.1371/journal.pone.0285662 (PMC10174501; doi:10.1371/journal.pone.0285662)
Supplement: Attachment — Submitted filename: 2 PONE-D-22-10622_R2 (1).pdf [file pone.0285662.s006.pdf]

PLOS ONE

Experiences, Barriers, and Facilitators of Health Data Use among Performance Monitoring Teams (PMT) of Health Facilities in Eastern Ethiopia: A Qualitative Study.

--Manuscript Draft--

Manuscript Number:	PONE-D-22-10622R2
Article Type:	Research Article
Full Title:	Experiences, Barriers, and Facilitators of Health Data Use among Performance Monitoring Teams (PMT) of Health Facilities in Eastern Ethiopia: A Qualitative Study.
Short Title:	Barriers and facilitators of health data use by Performance Monitoring Teams
Corresponding Author:	Admas Abera, MSc in Epidemiology<br>Haramaya University College of Health and Medical Sciences<br>HARAR, ETHIOPIA
Keywords:	Performance monitoring team, data use, barriers, facilitators, health facilities, CFIR framework, Ethiopia.
Abstract:	Abstract<br>Background: Routine health data is crucial in decision-making and improved health outcomes. Despite the significant investments in improving Ethiopia's Performance Monitoring Team (PMT), there is limited evidence on the involvement, implementation strategies, and facilitators and barriers to data utilization by these teams responding to present and emerging health challenges. Therefore, this study aimed to explore the PMT experiences, facilitators, and barriers to information used in healthcare facilities in Eastern Ethiopia.<br>Method: This study employed a phenomenological study design using the Consolidated Framework for Implementation Research (CFIR) to identify the most relevant constructs, aiming to describe the data use approaches at six facilities in Dire Dawa and Harari regions in July 2021. Key informant interviews were conducted among 18 purposively selected experts using a semi-structured interview guide. Thematic coding analysis was applied using a partially deductive approach informed by previous studies and an inductive technique with the creation of new emerging themes. Data were analyzed thematically using ATLAS.ti.<br>Results: Study participants felt the primary function of PMT was improving service delivery. This study also revealed that data quality, performance, service quality, and improvement strategies were among the major focus areas of the PMT. Data use by the PMT was affected by poor data quality, absence of accountability, and lack of recognition for outstanding performance. In addition, the engagement of PMT members on multiple committees negatively impacted data use leading to inadequate follow-up of PMT activities, weariness, and insufficient time to complete responsibilities.<br>Conclusion: Performance monitoring teams in the health facilities were established and functioning according to the national standard. However, barriers to operative data use included PMT engagement with multiple committees, poor data quality, lack of accountability, and poor documentation practices. Addressing the potential barriers by leveraging the PMT and existing structures have the potential to improve data use and health service performance.
Order of Authors:	Admas Abera, MSc in Epidemiology
	Tilahun Shiferaw, MSc in Information Science
	Abebe Tolera, MPH EPidemiology
	Biruk Shalmeno
	Adisu Birhanu Weldesenbet
	Assefa Tola
	Alemayehu Girma
	Rania Mohammed

Powered by Editorial Manager® and ProduXion Manager® from Aries Systems Corporation

	Yadeta Dessie
**Response to Reviewers:**	The comments and questions raised by both the academic editor and reviewer were insightful and helped improve our manuscript. We have gone through each comments raised and attached under the authors responses in the system. We would like to express our deep gratitude. Thank you!
**Additional Information:**	
**Question**	**Response**
**Financial Disclosure**<br><br>Enter a financial disclosure statement that describes the sources of funding for the work included in this submission. Review the submission guidelines for detailed requirements. View published research articles from *PLOS ONE* for specific examples.<br><br>This statement is required for submission and **will appear in the published article** if the submission is accepted. Please make sure it is accurate.<br><br>**Unfunded studies**<br>Enter: *The author(s) received no specific funding for this work.*<br><br>**Funded studies**<br>Enter a statement with the following details:<br>• Initials of the authors who received each award<br>• Grant numbers awarded to each author<br>• The full name of each funder<br>• URL of each funder website<br>• Did the sponsors or funders play any role in the study design, data collection and analysis, decision to publish, or preparation of the manuscript?<br>• **NO** - Include this sentence at the end of your statement: *The funders had no role in study design, data collection and analysis, decision to publish, or preparation of the manuscript.*<br>• **YES** - Specify the role(s) played.<br><br>* typeset	This work would not be possible without the financial support of the Doris Duke Charitable Foundation (DDCF) under grant number 2017187. The mission of the Doris Duke Charitable Foundation is to improve the quality of people’s lives through grants supporting the performing arts, environmental conservation, medical research, and child well-being, and through the preservation of the cultural and environmental legacy of Doris Duke’s properties. The funders had no role in study design, data collection and analysis, decision to publish, or preparation of the manuscript. The funder website can be found here: https://www.ddcf.org/
**Competing Interests**<br><br>Use the instructions below to enter a competing interest statement for this submission. On behalf of all authors, disclose any competing interests that	The authors have declared that no competing interests exist.

Powered by Editorial Manager® and ProduXion Manager® from Aries Systems Corporation

could be perceived to bias this work—acknowledging all financial support and any other relevant financial or non-financial competing interests.<br><br>This statement is required for submission and **will appear in the published article** if the submission is accepted. Please make sure it is accurate and that any funding sources listed in your Funding Information later in the submission form are also declared in your Financial Disclosure statement.<br><br>View published research articles from *PLOS ONE* for specific examples.<br><br>**NO authors have competing interests**<br><br>Enter: *The authors have declared that no competing interests exist.*<br><br>**Authors with competing interests**<br><br>Enter competing interest details beginning with this statement:<br><br>*I have read the journal's policy and the authors of this manuscript have the following competing interests: [insert competing interests here]*<br><br>* typeset	
**Ethics Statement**<br><br>Enter an ethics statement for this submission. This statement is required if the study involved:<br><br>• Human participants<br>• Human specimens or tissue<br>• Vertebrate animals or cephalopods<br>• Vertebrate embryos or tissues<br>• Field research<br><br>Write "N/A" if the submission does not require an ethics statement.	The ethical approval and clearance for this research was obtained from Institutional Health Research Ethics Review Committee (IHRERC) of Haramaya University with reference number IHRERC/196/2020. Permission was sought from regional health bureaus and the health facilities. Before data collection, informed written consent was obtained from the study participants after explaining the study aims and their right to withdraw from the study at any time.

Powered by Editorial Manager® and ProduXion Manager® from Aries Systems Corporation

General guidance is provided below. Consult the submission guidelines for detailed instructions. **Make sure that all information entered here is included in the Methods section of the manuscript.**<br><br>**Format for specific study types**<br><br>**Human Subject Research (involving human participants and/or tissue)**<br>• Give the name of the institutional review board or ethics committee that approved the study<br>• Include the approval number and/or a statement indicating approval of this research<br>• Indicate the form of consent obtained (written/oral) or the reason that consent was not obtained (e.g. the data were analyzed anonymously)<br><br>**Animal Research (involving vertebrate animals, embryos or tissues)**<br>• Provide the name of the Institutional Animal Care and Use Committee (IACUC) or other relevant ethics board that reviewed the study protocol, and indicate whether they approved this research or granted a formal waiver of ethical approval<br>• Include an approval number if one was obtained<br>• If the study involved *non-human primates*, add *additional details* about animal welfare and steps taken to ameliorate suffering<br>• If anesthesia, euthanasia, or any kind of animal sacrifice is part of the study, include briefly which substances and/or methods were applied<br><br>**Field Research**<br><br>Include the following details if this study involves the collection of plant, animal, or other materials from a natural setting:<br>• Field permit number<br>• Name of the institution or relevant body that granted permission	
**Data Availability**<br><br>Authors are required to make all data	Yes - all data are fully available without restriction

underlying the findings described fully available, without restriction, and from the time of publication. PLOS allows rare exceptions to address legal and ethical concerns. See the PLOS Data Policy and FAQ for detailed information.<br><br>A Data Availability Statement describing where the data can be found is required at submission. Your answers to this question constitute the Data Availability Statement and **will be published in the article**, if accepted.<br><br>**Important:** Stating 'data available on request from the author' is not sufficient. If your data are only available upon request, select 'No' for the first question and explain your exceptional situation in the text box.<br><br>Do the authors confirm that all data underlying the findings described in their manuscript are fully available without restriction?	
**Describe where the data may be found in full sentences. If you are copying our sample text, replace any instances of XXX with the appropriate details.**<br><br>• If the data are **held or will be held in a public repository**, include URLs, accession numbers or DOIs. If this information will only be available after acceptance, indicate this by ticking the box below. For example: *All XXX files are available from the XXX database (accession number(s) XXX, XXX.).*<br>• If the data are all contained **within the manuscript and/or Supporting Information files**, enter the following: *All relevant data are within the manuscript and its Supporting Information files.*<br>• If neither of these applies but you are able to provide **details of access elsewhere**, with or without limitations, please do so. For example:<br><br>*Data cannot be shared publicly because of [XXX]. Data are available from the*	All relevant data are within the manuscript.

XXX Institutional Data Access / Ethics Committee (contact via XXX) for researchers who meet the criteria for access to confidential data.<br><br>*The data underlying the results presented in the study are available from (include the name of the third party and contact information or URL).*<br>• This text is appropriate if the data are owned by a third party and authors do not have permission to share the data.<br><br>* typeset	
Additional data availability information:	

Powered by Editorial Manager® and ProduXion Manager® from Aries Systems Corporation

Manuscript

Click here to access/download;Manuscript;Revised_Manuscript_Clean_V3.do

Experiences, Challenges, and Facilitators of Health Data Use among Performance Monitoring Teams (PMT) of Health Facilities in Eastern Ethiopia: A Qualitative Study.

Admas Abera[1]*, Abebe Tolera[1], Biruk Shalmeno[1], Adisu Birhanu Weldesenbet[1], Assefa Tola[1], Tilahun Shiferaw[2], Alemayehu Girma[3], Rania Mohammed[4] and Yadeta Dessie[1]

[1]School of Public Health, College of Health and Medical Sciences, Haramaya University, Harar, Ethiopia.

[2]Department of Information Sciences, College of Computing and Informatics, Haramaya University, Haramaya, Ethiopia.

[3]Policy and Plan Directorate, Dire Dawa Administration Health Bureau, Dire Dawa, Ethiopia.

[4]Policy and Plan Directorate, Harari Regional Health Bureau, Harar, Ethiopia.

***Corresponding author:**

Email: admasabera10@gmail.com

Abstract

Background: Routine health data is crucial in decision-making and improved health outcomes. Despite the significant investments in improving Ethiopia's Performance Monitoring Team (PMT), there is limited evidence on the involvement, implementation strategies, and facilitators and barriers to data utilization by these teams responding to present and emerging health challenges. Therefore, this study aimed to explore the PMT experiences, facilitators, and barriers to information used in healthcare facilities in Eastern Ethiopia.

Method: This study employed a phenomenological study design using the Consolidated Framework for Implementation Research (CFIR) to identify the most relevant constructs, aiming to describe the data use approaches at six facilities in Dire Dawa and Harari regions in July 2021. Key informant interviews were conducted among 18 purposively selected experts using a semi-structured interview guide. Thematic coding analysis was applied using a partially deductive approach informed by previous studies and an inductive technique with the creation of new emerging themes. Data were analyzed thematically using ATLAS.ti.

Results: Study participants felt the primary function of PMT was improving service delivery. This study also revealed that data quality, performance, service quality, and improvement strategies were among the major focus areas of the PMT. Data use by the PMT was affected by poor data quality, absence of accountability, and lack of recognition for outstanding performance. In addition, the engagement of PMT members on multiple committees negatively impacted data use leading to inadequate follow-up of PMT activities, weariness, and insufficient time to complete responsibilities.

Conclusion: Performance monitoring teams in the health facilities were established and functioning according to the national standard. However, barriers to operative data use included PMT engagement with multiple committees, poor data quality, lack of accountability, and poor documentation practices. Addressing the potential barriers by leveraging the PMT and existing structures have the potential to improve data use and health service performance.

Key words: performance monitoring team, data use, barriers, facilitators, health facilities, Ethiopia.

Introduction

Health systems are complex and continually changing to adapt to political, economic, social, technological, and epidemiological realities within constrained resources, particularly in low- and middle-income countries (LMICs) [1-3]. Health facilities must cope with these changing realities through organized management and leadership, which require reliable data for developing a comprehensive policy package for health sector reforms [2, 4].

There has been an increased need for health service performance strengthening to manage population needs through effective leadership [5-7], and an improved health facility data use culture [8-10]. The increasing demand and capacity to use data appear more critical than the expanding supply of evidence [3,13] for improved access and quality of care [4,14]. Collection, processing, transforming, communicating, and using service delivery reports and administrative records are crucial in decision-making for improved health outcomes [15, 16].

Data use is the process through which decision-makers and stakeholders explicitly evaluate information in one or more steps of policymaking, program planning, management, or service provision [6]. In the Ethiopian context, the PMT is a multidisciplinary health workforce team primarily responsible for data use [11, 12]. By effectively using the data, targeted health service delivery improvements better meet the population's needs [17].

However, in many developing countries, the quality of the evidence needed to generate valid information to make decisions about health programs could be better or more [18-20], mainly due to an ineffective data use culture [20]. The major bottlenecks for information use include inadequate infrastructure, leadership turnover, dysfunctional external relations [26], deficient data collection, and limited resources [15]. In addition, a shortage of skilled workforce, an imbalance in skills, geographic misdistribution, difficulty in inter-professional collaboration, inefficient use of resources, and burnout were found to affect the health service quality [21].

To date, most efforts to strengthen the health information systems have primarily focused on digitization, improving data quality and analysis, and identifying problems; however, the ultimate goal is utilizing information to problem solve, which requires time to build an information use culture [22]. Therefore, engagement (including involvement, commitment, effort or observable behaviour, a positive effect, or some combination of these) of healthcare leaders and managers is

pivotal if we are to improve the health information system (HIS) and hence, the health service delivery [4, 13].

The engagement level and capacities of health managers and performance monitoring teams to respond to current and emerging issues still need to be better understood [21]. Without radical structural and systemic changes, the existing governance structures and management systems will continue to fail to address the gaps in health service delivery [2]. Furthermore, despite significant investments in performance monitoring teams in Ethiopia, a joint performance renewal effort must be improved. Finally, there is a paucity of studies addressing the facilitators, and challenges influencing health data usage to improve primary health care delivery in LMICs. Therefore, this study aimed to identify the PMT experiences, facilitators and barriers to information used in the healthcare facilities in the Harari region and Dire Dawa city administration in Eastern Ethiopia.

Methods and Materials

Study Setting

The study was conducted in selected Dire Dawa and Harari public health facilities. Dire Dawa is located 515 kilometers from Addis Ababa and has an estimated population of 341,834, with 68.23% living in urban areas [23]. Data from the public health facilities indicate 15 health centers (8 urban and seven rural), two hospitals, and 32 health posts under the city administration. Harar is the capital of Harari, one of the regional states of Ethiopia, located 517 Km east of Addis Ababa and 48 km south of Dire Dawa. According to the 2007 census, the region has a total population of 183,415 people [23], and more than half (54.18%) reside in urban areas [24]. The region has three government hospitals, one university teaching hospital, two private hospitals, nine health centers, and 24 health posts.

Study Design and Participants

This study used a phenomenological study design. The Consolidated Framework for Implementation Research (CFIR) was used to identify the most relevant constructs to describe data use approaches. The CFIR framework is an evidence-based framework from multiple disciplines providing a comprehensive arrangement of paradigms that influence complex implementations. The framework has five significant domains (inner setting, outer setting, intervention

characteristics, individuals involved, and implementation process) with associated components used in this study.

Two hospitals and four health centers were randomly selected from a list of facilities in the two regions. A purposive sampling technique was employed to select the key informants. Eighteen interviews were conducted until data saturation was achieved. The key informants were PMT members from the health facilities, including hospital medical directors, heads of health centers, heads of departments (outpatient, inpatient, emergency, maternal and child health, pharmacy, laboratory, nursing, human resource, and finance team leads), and the Health Management Information System (HMIS) officers.

Data Collection

We conducted key informant interviews using a semi-structured interview guide. The tool was comprised of questions related to the respondents' socio-demographic characteristics, the process of the PMT establishment and implementation strategy process, data use processes and practices, barriers to data use, motivating factors for data use, and recommended mechanisms to improve the data use practice.

Data Quality Control

Data were collected between July 05, 2021, and July 24, 2021, by interviewers fluent in local languages (Amharic and Afan Oromo) and English, with graduate-level education and experience in qualitative data collection. All data collection team members underwent a one-day training workshop to understand the interview guide and study objectives. The data collection activities were supervised daily by the study investigators. All interviews were conducted privately and lasted 20-60 minutes.

Data Processing and Analysis

The interviews were transcribed verbatim, and notes were taken in the field. The interviews were translated into English and translated back to the original languages to confirm the accuracy. A codebook was developed based on the initial review of the transcripts, and transcripts were systematically coded using ATLAS.ti software. Double coding was initially used, with disagreements resolved by discussion; updates to the code definitions were made when needed. Double coding continued until no new disagreements were identified. Subsequently, summaries

for each transcript were written under each code using a matrix. Thematic data analysis described and compared general statements as relationships, themes, and sub-themes emerged in the data. Accordingly, findings were categorized into five themes. Under each theme, sub-themes were defined with verbatim quotes representing opinions to substantiate the results. Researchers read all interview transcripts, counterchecked the transcripts, coded the data, and agreed on the emerging themes and sub-themes. Additionally, research rigor was enhanced through regular discussions between researchers.

Key Themes and Sub-themes

In this study, five key themes and 16 sub-themes emerged. The main themes and sub-themes are presented in Table 1.

Table 1: Categories of Key themes and sub-themes emerged.

**Themes**	**Sub-themes**
Membership and roles of PMT members at the health facilities	• *Roles and responsibilities of PMT members*<br>• *The current PMT status and experiences*
Relevance of PMT strategies and its implementation	• *Perceived benefits of PMT*<br>• *The modality of PMT meetings*<br>• *Adequacy and convenience of time of PMT meeting*<br>• *The focuses of PMT meetings*
Barriers to data use in the facility	• *Organizational barriers*<br>• *Healthcare workers' work ethic and behavioral barriers*<br>• *COVID-19 effect on data use*
Motivational mechanisms and demotivating factors in enhancing data use practices in place for PMT members	• *Capacity building practices for PMT members*<br>• *Recognition and non-monetary incentives*

	• *Demotivating factors*
Recommended mechanisms to improve the data use practices	• *Performance-based recognition*<br>• *Continuous supply of resources, and improving HIS infrastructures*<br>• *Strengthening the overall HIS*<br>• *Enhancing the quality of routine data*

Ethical Considerations

Ethical clearance was obtained from Haramaya University, College of Health and Medical Sciences, Institutional Health Research Ethics Review Committee (IHRERC) (reference number IHRERC/196/2020**)**. In addition, permission was sought from Dire Dawa Administration and the Harari Regional Health Bureaus, and the studied health facilities. Before data collection, informed written consent was obtained from the study participants. Furthermore, participants were assured of the confidentiality of the information and their right to withdraw from the study at any time during the study. This paper omitted personal identifiers to maintain confidentiality, while neutral identifiers and participants' age were mentioned in direct quotes.

Results

Socio-demographic characteristics

A total of 18 in-depth interviews were conducted in two hospitals and four health centers to explore the PMT experience, barriers, and facilitators of data use. Of these participants, eight were females, and three were health facility heads with a median age (IQR) of 31 (7) years. (Table 2)

Table 2: Socio-demographic characteristics of key informants at the health facilities, Eastern Ethiopia 2021.

Participant ID	Sex	Age in years	Educational status	Work experience in years	Facility type
KI1	Female	34	BSc	11	Health center
KI2	Female	25	Diploma	2	Health center
KI3	Male	27	BSc	9	Health center
KI4	Female	38	BSc	20	Health center
KI5	Male	26	BSc	7	Health center
KI6	Male	34	BSc	13	Health center
KI7	Male	28	Master's	3	Health center
KI8	Female	25	BSc	1	Health center
KI9	Female	29	BSc	6	Referral Hospital
KI10	Male	28	BSc	32	Referral Hospital
KI11	Male	32	BSc	13	Health center
KI12	Male	30	BSc	7	Health center
KI13	Female	34	BSc	8	Health center
KI14	Female	39	BSc	12	Health center
KI15	Male	42	BSc	19	General Hospital
KI16	Male	35	BSc	3	General Hospital
KI17	Female	35	BSc	12	General Hospital
KI18	Female	30	BSc	10	General Hospital

1. Membership and Roles of PMT at the health facilities

Participants reported that health facilities use specific criteria to select the members of the PMT, including the head of the quality department, HMIS unit team leader, department head, management team, and staff with good performance.

> A respondent stated, "…*since the performance of every department is reviewed in the meeting, the main criteria for PMT membership is being a department head. The heads of management unit, HMIS unit, and quality department are also included. I became a member because I have served as a vice matron for the last one and half years*." (KI9, 29 years old)

> Another key informant pointed out;

> "…*any health care provider in a case team can be a member of the PMT. That means there will be one representative from each case team in PMT irrespective of his/her status."* (KI15, 42 years old)

Roles and responsibilities of PMT members include use of health data to improve the health service performance. The specific roles include preparing action plans, preparing directions to improve service delivery, comparing performance overtime, monitoring monthly performance, providing feedback to various units, building the capacity of health workers, and preparing reports. The present study also found that the PMT members were aware of their responsibilities.

> One of the participants stated that "…*each member of the PMT is expected to prepare and submit their case team or department reports to the head of the health facility on time. All PMT members will discuss on the submitted report during the monthly PMT meeting in which the reports are crosschecked for its consistency and quality. When the achievements are lower than expected, the reasons for underachievement are sorted out and future directions are put to improve the performance*." (KI4, 38 years old)

The participants' opinions on aligning the PMT's roles and responsibilities at the facility with their activities were mixed. While the majority of respondents stated that PMT tasks and responsibilities are aligned with their actual activities, some stated they do not match with

their actual activities. There is some overlapping of duties and roles, according to these participants.

"As a case team leader, my responsibility is identifying the challenges in my activities but sometimes the roles and activities rendered to us are not related to service performance and members get frustrated due to that" (KI16, 35 years old)

2. Relevance of PMT strategy and its implementation

The perceived benefits of PMT for the facility include improving the service delivery and customer satisfaction. Additionally, this team is essential for sharing experiences and skills among the departments and case teams.

"... the members of PMT are from different departments; there is knowledge sharing on different topics including preparation of reports and how to improve performance." (KI11, 32 years old)

Planning, identifying gaps through root cause analysis, and intervening on the identified problems, and monitoring and evaluating specific activities.

The key informants further pointed out, *"...we select poorly performed activities and the responsible departments will design an action plan for the identified problems by conducting a root cause analysis. Then, the department will be directed to monitor the implementation of the action plan and they are expected to bring the progress in the subsequent meetings."* (KI17, 35 years old)

The modality of PMT meetings. Health facilities have a monthly meeting plan in their health facilities, which is prepared in advance in the annual plan. Respondents pointed out that the monthly meetings were conducted immediately after each unit submitted their monthly report.

"Our PMT meeting is conducted on monthly basis. Immediately after the submission of the monthly report (usually at 21st day of the month), we hold our PMT meeting at 23rd or 24th day of the month. Substitution date will be posted on our telegram page if a meeting is rescheduled." (KI12, 30 years old)

Adequacy and convenience of timing of PMT meeting. On the other hand, it was found that the time allocated for PMT meetings was inadequate and inconvenient. All participants unanimously agreed that there should be enough time assigned and a convenient time allocated for the PMT meetings.

> *"The meeting time is not enough because every participant usually come to the meeting by interrupting their work. Usually, the meeting date and time is decided by the head."* (KI16, 35 years old)

Participants discussed how holding the meeting on weekends could reduce absenteeism and unnecessary interruptions.

> "…p*reviously, we used to hold the PMT meeting during working hours and hence the duration of the meetings was not adequate. This was due to high workload of the PMT members. However, currently we are conducting our PMT meeting on Saturdays. Since it is not a working day, PMT members are paid some pocket money for attending the meeting on over the weekend."* (KI8, 25 years old)

The focus of PMT meetings reportedly included data quality, performance activities (monthly, quarterly, or annual performance), service quality, and service improvement strategies. According to participants, the first agenda of the PMT meeting was evaluating the previous report, and then comparing it with the current performance. Next, the team develops an action plan based on the identified gaps. However, in some health facilities, the previous performance is not conducted at all.

> "*In our monthly PMT meeting, the first thing is presenting the monthly performance report for each unit. Based on the report, gaps and challenges are identified, before action plans are drafted on the identified gaps*". (KI9, 29 years old)

The PMTs mainly utilize routine data for evaluation and monitoring of service delivery programs. Therefore, there needed to be a culture of using health related data from other sources, such as surveys, assessments, or research findings.

"*In our health center, the main target of PMT is the routine data. The data accuracy and completeness are checked. Service improvement strategies are also discussed.*" (KI5, 26 years old)

The respondent further stated *"data collected in our health facility is used for making decisions. I have not seen other data sources from researches or surveys being utilized so far."* (KI5, 26 years old)

3. Major barriers to data use in the facility

The organizational barriers to data use at the facility level were poor data quality, being a member of multiple committees and high patient flow, human resource related issues, input related issues, inadequate budget allocation, and lack of performance-based incentives.

"…PMT members have their own duties in addition to the PMT activities which makes it difficult to collect quality data due to competing priorities. If someone is engaged with overlapping duties, the desired outcome may not be achieved". (KI3, 27 years old)

Participants reported that they were in at least two other committees in their health facility. This resulted in poor follow-up of the designated PMT activities and time constraint with committee meetings and assignments. According to the participants, multiple committees negatively affected the effectiveness of the PMT activities.

"*…being the member of several committees affects the PMT activities. The aim of including a person in a given team or committee is to perform the task effectively and efficiently in order to bring the intended changes. However, the focus of the individual is usually divided to different committees. In most cases the meeting times is overlapping between different committees"* (KI2, 25 years old)

It was further pointed out:

"Participation in multiple committees decreases the effectiveness of the PMT. Not only for the PMT, but even the other committees are also adversely affected. I suggest to not assign case team leaders on more than one committee." (KI14, 39 years old)

The routine data quality collected at the health facilities was reported as a barrier for effective data use.

> *"There is a gap in data quality including gaps with data consistency and completeness. Healthcare providers perform their daily activities but they do not document it on the register regularly. The data from HMIS may contradict from your observation every day. Hence it is difficult to use our data for decision making due to its poor quality."* (KI2, 25 years old)

> Another participant further pointed out *"A timely, reliable and high-quality data should be generated in order to use the data for decision making. Monitoring and supporting the staffs is also critical to obtain high quality data."* (KI17, 35 years old)

HIS input related factors, including a shortage of patient registrations was cited as another barrier for data use.

> *"We have an old register called daily register for documentation of our data. This register was supplied by a Non-Governmental Organization. However, this supply of registers has stopped and currently, we have shortage of the daily registers which affected our documentation."* (KI13, 34 years old)

An additional barrier for effective data use at the health facilities was budget constraints, which was felt to influence documentation in the facilities.

> *"We use the budget given for the health center, but there is no budget allocated specifically for HMIS."* (KI6, 34 years old)

Healthcare providers' work ethic and behavioural barriers were another sub-theme which emerged. Poor commitment and lack of accountability from healthcare providers and PMT members were the most reported challenges. One respondent explained the commitment and competing priorities of PMT members as

> *"…sometimes we reviewed the same problem repeatedly without a solution. We start to get fed up and start to wonder when it will be solved. Sometimes, we skip the meeting intentionally for this reason.* (KI9, 29 years old)

Another respondent pointed out that:

> *"…health workers that reported incomplete or false data are not given any administrative reprimands for their wrong doing.*" (KI18, 30 years old)

Furthermore, a major challenge is a lack of understanding of the data value by the healthcare workers when using data for action.

> *"Sometimes the PMT members' attitude towards data is a challenge by itself. The notion that a high-quality data can change the hospital is not understood equally among the staffs."* (KI9, 29 years old)

According to the participants, most of the health professionals have a poor understanding and attitude regarding the importance of data in improving the quality of health services. Additionally, according to the participants there are gaps in skills in analyzing data using electronic computing software, such as District Health Information System version 2 (DHIS2).

> *"Disparity in understanding the importance of data among our staffs is the other problem. While some people have better understanding towards data, others do not see the value of it."* (KI9, 29 years old)

One respondent further noted:

> *"There is a problem on registering the data and handling of a registers. Even after the data is entered into the DHIS2, there is skill gap in analyzing it."* (KI10, 32 years old)

Poor tracking of problems, and a lack of monitoring of action plans is frequently observed according to the key informants.

> *"The main problem is that we don't strictly follow the action plans designed in the previous PMT meetings."* (KI2, 25 Years old)

Absenteeism and interruption due to competing priorities were also reported during PMT meetings.

"As I said earlier, even if it is a monthly meeting, the date and time should be notified ahead of time. Sudden meetings will affect my routine activities and as a result some members of the PMT tend to be absent and some interrupt the meeting to go back to their urgent activities." (KI13, 34 years old)

Poor documentation practices were another challenge reported by PMT members. First, in order to create a change in the data use culture, the first priority should be changing the attitude of the health care workers before other interventions are introduced.

"*Health professionals should believe documenting data primarily benefits patients, themselves and their health facility. Therefore, working on the attitude change towards documentation should be given more emphasis."* (KI11, 32 years old)

COVID-19 effect on data use: The COVID-19 pandemic had direct and indirect effects on data use practices at the health facilities. An informant explained the effect of COVID-19 pandemic on their day-to-day activity as

"*Due to the occurrence of COVID -19 outbreak, the nearby hospital was entirely devoted to provide health care service for COVID-19 cases. Hence, all patients in the catchment area of that hospital came to our health center. These conditions increased the workload and compromised our routine activities*." (KI14, 39 years old)

Another key informant stated *"…due to COVID-19, the head of HMIS has been in quarantine for the last two months which negatively affected the PMT activities."* (KI3, 27 years old)

4. Motivation mechanism in place for PMT members at the facilities

This study identified several motivation mechanisms in place though not across all facilities and not conducted regularly. The main motivation strategies reported were capacity building and performance-based recognition.

Capacity building practices for PMT members: According to participants, the primary capacity building practices included in-service trainings, recognition for good performance, and professional development.

"Capacity building on data quality and project development for the members of PMT has been a very good incentive since we never had such experience before." (KI7, 28 years old)

Recognition and Non-monetary incentive mechanisms to motivate the PMT members in the facility were reported. Participants indicated that these motivational mechanisms were effective when directed towards improving the data use culture of the facilities.

A participant pointed out that "*performance-based recognition and motivation of staffs can improve the data quality and subsequently the data use in the facility."* (KI6, 34 years old)

However, in some health facilities there were not any motivation measures specific to PMT members.

"There are no capacity buildings, recognition mechanisms, and career enhancing educational opportunities to motivate PMT members." (KI4, 38 years old)

Other participants reported that some sort of recognition and motivation mechanisms were implemented in their facilities.

"They awarded me this watch for my good performance for properly ensuring the quality of data collected in my health facility." (KI7, 30 years old)

Others believed that incentives may not necessarily motivate staff to use the data or improve service performance.

"It is hard to say that the presence of incentives only positively affects data use. Providing incentives before attitude change may even adversely affect the data use practice." (KI11, 32 years old)

Demotivating factors for effective data use practices were cited by the study participants including a shortage of resources, inadequate salary, and inadequate follow-up.

"*Salary is one of the demotivating factors because it does not fit with the job that we undertake.*" (KI10, 28 years old)

"There is no follow up mechanism after trainings are provided." (KI8, 25 years old)

The managers' leadership skills was one of the factors affecting data use in health facilities. Health managers' and case-team/departments' poor leadership skills, and lack of value for data were also mentioned as demotivating factors.

"The head of the facility lacks decisiveness and leadership qualities on this job. I don't think the problem is a gap in knowledge but rather an attitude problem." (KI13, 34 years old)

Another respondent pointed out "*…if the head of the department or the facility do not utilize data properly, other employees will not care about data use. Hence, the head at district, region or at any other level should lead by example to motivate other staffs."* (KI2, 25 years old)

5. Recommended motivation mechanisms to improve data use

Performance-based recognition was recommended by all informants as a major motivation mechanism to improve data use.

"There are those who register and report on time among the health extension workers. The others will be motivated to do the same if you provide recognition or incentives. The incentive can even be in the form of a certificate." (KI15, 42 years old)

Another participant stated, *"since the PMT members are the backbone of health facilities, designing motivating mechanisms is important."* (KI9, 29 years old)

Continuous supply of resources, improving the infrastructures, and strengthening the HIS.

"*Ensuring continuous supply of necessary materials and recognizing best performers should be introduced.*" (KI9, 29 years old)

Enhancing the quality of routine data collected was also unanimously believed to enhance data use practice.

"In order to use data for a decision, first I have to receive the data properly. A timely, reliable and complete data should be collected." (KI1, 34 years old)

Discussion

This study explored experiences, facilitators and barriers of the PMT, using the adapted Consolidated Framework for Implementation Research (CFIR), to generate information for decision-making at points of healthcare delivery The findings revealed that the PMT in most facilities were established according to the standard criteria set by the Ministry of Health (MOH) of Ethiopia [12]. In line with previous studies, our study indicated that the primary responsibility of the PMT was to improve data quality and regularly monitor progress and improve health service performance [11, 12].

Although some irregularities were reported, the monthly meetings were conducted right after each unit submitted their monthly report and before submitting their report to the next level to monitor progress and improve performance. The MOH guideline indicates that the meeting dates, venue and its members should be officially communicated in advance and the meeting should be conducted at least a day ahead of submission of the monthly report to the next level [11, 12, 25].

The present study found the PMT meetings focused on data quality, performance improvement, and evaluation of previous action plans. Previous studies show PMT meetings should start with follow-up activities for gaps highlighted from the previous PMT meeting as the first item on the agenda, followed by an assessment of the progress on those gaps [12, 25]. Meetings and collected data have no value unless action items from meetings are implemented and data are analyzed into meaningful actions [26].

Health facilities should design strategies to minimize the number of committees and integrate similar committees to improve their service provision. Most PMT members in our study were usually involved in at least two other committees in their health facility. This creates poor follow up of the activities set out by the PMT with the committee members being overburdened by meetings and assignments, creating fatigue and a shortage of time to accomplish PMT assignments.

Evidence indicates data triangulation through other sources, such as original research, community feedback, expert opinions, vital registration, censuses, and routine HMIS data can yield better results [12,25,26]. Although there were practice of use of routine internal data, information use from external sources was limited.

Previous studies indicated that an organizational context that supports data collection, availability, and use, the technical aspects of data processes and tools, and the behavior of individuals who produce and/or use data are the main elements of health information use [25, 27]. The major challenges of data use reported in the present study emanate from organizational, behavioral and technical sources including poor data quality, competing priorities, shortage of skilled human power, and lack of performance-based motivation for the health workers.

Healthcare organizations are increasingly required to gather and report data about their performance and respond to the results of consequential quality measurements [28]. Quality data enables healthcare organizations to monitor progress, making decisions for continuous improvement, and provide effective and efficient health services [29]. Our study revealed that although poor quality of data was one of the major challenges for an informed decision making, it has been used for priority setting as well as designing and implementing action plans. This requires attention as decisions taken by healthcare managers might be misleading and fails to address the actual problem. Previous study Addis Ababa indicated that the PMT meetings that were designed for the sole purpose of improving data quality were not effective [30]. Another qualitative study to explore the facilitators and barriers of digital health technology use also identified data quality as a potential barrier [31]. Lack of available data for several indicators and a lack of validated indicators for important dimensions of quality were identified as major challenges to improve Primary Health Care (PHC) Performance [6].

Behavioral factors provide crucial insight into the way in which health workers, managers and policymakers use information [25]. The attitude, motivation, and value decision makers attach to information play a big role in determining data use [2, 25]. Concurrent with these, poor commitment, skill gap, lack of accountability for failing to accomplish tasks, work overload, poor documentation, and resource mismanagement were the main behavioural level challenges reported in this study.

Engagement with different committees increased PMT workload and compromised the effectiveness of data use. Participation in quality improvement collaborative activities may improve health professionals' knowledge, problem-solving skills and attitude; teamwork; shared leadership and habits for improvement. A review of literature indicated shifting roles and

expectations in the workplace for health care leadership and management as a major challenge for health leadership and workforce [1]. Interaction across quality improvement teams may generate normative pressure and opportunities for capacity building and peer recognition [11].

Health professionals either do not consider recording and reporting data as part of their routine activities or they just give more priority to the clinical provision and lesser attention to data [30]. Although there is a need to make significant investments in workforce development and training [33], tackling of behavioral factors require interventions that go beyond simple trainings and should focus more on initiatives that enhances positive attitude towards data value [25].

The individual determinant of motivation among healthcare workers are altruism, attaining professionalism, educational opportunity, and being more experienced [32]. In the present study, it was indicated that health facilities use capacity buildings, and some form of non-monetary incentives to improve performance, while most informants indicated dissatisfaction with the absence of such motivating mechanisms. Career development opportunities, in-service trainings, and regular recognition for good performance were also reported to be practiced at the health facilities.

Provision of inputs necessary for HIS such as laptops, internet modems; as well as awarding the best performing health facilities were other motivational methods. Since health professionals can be motivated by a range of extrinsic and intrinsic factors, policy makers need to look beyond traditional financial incentives when designing policies to improve health service performance. [34] Studies indicate training opportunities, transformative leaders, and nonmonetary incentives, staff development and promotion, availability of necessary resources, and ease of communication are found to be effective motivation mechanisms for health care workers [32, 35].

Conclusions

This study has generated important insights into PMT establishment, implementation strategies, barriers to data use, and recommendations to enhance data use practice at the point of healthcare service delivery. The study found that most performance monitoring teams in health facilities were established and functioning according to the national standard. Additionally, the study revealed that the barriers to effective data use include the PMT attending multiple committee meetings

(increasing workload), poor data quality, lack of accountability, and poor documentation practices. Recommendations to enhance data use practices include non-monetary incentives and recognition for exemplary health workers. Improving routine data quality, integrating various teams into PMT, establishing an accountability framework, and designing documentation methods can all improve informed decision-making. While a comprehensive countrywide study of PMTs is required, policymakers, stakeholders working in HIS, and health managers should work on improving routine data quality and design motivational strategies, including recognition and non-monetary incentives to improve data use which has the potential to improve health service performance.

Acknowledgements

The authors are thankful to the Ethiopian Ministry of Health, Dire Dawa and Harari Regional Health Bureaus. We are also grateful for participating health facilities and their healthcare staffs for their time and voluntarily for contributing to the research. Moreover, we would like to acknowledge our data collectors and logistic facilitators for their support throughout the study.

References

1. Figueroa CA, Harrison R, Chauhan A, Meyer L. Priorities and challenges for health leadership and workforce management globally: a rapid review. *BMC Health Services Research*. 2019;19(1):239.

2. Senkubuge F, Modisenyane M, Bishaw T. Strengthening health systems by health sector reforms. *Global health action*. 2014;7(1):23568.

3. Witter S, Anderson I, Annear P, Awosusi A, Bhandari NN, Brikci N, et al. What, why and how do health systems learn from one another? Insights from eight low- and middle-income country case studies. *Health Research Policy and Systems*. 2019;17(1):9.

4. Nordsteien A, Byström K. Transitions in workplace information practices and culture. *Journal of Documentation*. 2018;74(4):827-43.

5. Veillard J, Cowling K, Bitton A, Ratcliffe H, Kimball M, Barkley S, et al. Better measurement for performance improvement in Low-and Middle-Income countries: the

primary health care performance Initiative (PHCPI) experience of conceptual framework development and indicator selection. *The Milbank quarterly*. 2017;95(4):836-83.

6. Tantivess S, Walt G. The role of state and non-state actors in the policy process: the contribution of policy networks to the scale-up of antiretroviral therapy in Thailand. *Health policy and planning*. 2008;23(5):328-38.

7. Fryatt R, Bennett S, Soucat A. Health sector governance: should we be investing more? *BMJ global health*. 2017;2(2):e000343.

8. Lippeveld T. Routine health facility and community information systems: creating an information use culture. Global Health: Science and Practice; 2017. p. 338-40.

9. Nordsteien A, Byström K. Transitions in workplace information practices and culture: The influence of newcomers on information use in healthcare. *Journal of Documentation*. 2018.

10. Jylhä V. Information management in health care: a model for connecting information culture and patient safety. 2017.

11. Adane T, Tadesse T, Endazenaw G. Assessment on Utilization of Health Management Information System at Public Health Centers Addis Ababa City Administrative, Ethiopia. *Internet Things Cloud Comput*. 2017;5(1):7-18.

12. MOH. Information Use Training Module. In: PPMED. Addis Ababa; June 2018.

13. Aqil A, Hozumi D, Lippeveld T. Tools for data demand and use in the health sector: Performance of Routine Information Systems Management (PRISM) Tools. *Measure Evaluation*. 2011.

14. Watson DE, Broemeling A-M, Reid RJ, Black C. A results-based logic model for primary health care : laying an evidence-based foundation to guide performance measurement, monitoring and evaluation. 2004.

15. Endriyas M, Alano A, Mekonnen E, Ayele S, Kelaye T, Shiferaw M, et al. Understanding performance data: health management information system data accuracy in Southern

Nations Nationalities and People's Region, Ethiopia. *BMC Health Services Research*. 2019;19(1):175.

16. MOH. Guidance on developing key performance indicators and minimum data sets to monitor healthcare quality. 2013.

17. Rendell N, Lokuge K, Rosewell A, Field E. Factors That Influence Data Use to Improve Health Service Delivery in Low- and Middle-Income Countries. *Global Health: Science and Practice*. 2020;8(3):566.

18. Maphumulo WT, Bhengu BR. Challenges of quality improvement in the healthcare of South Africa post-apartheid: A critical review. *Curationis*. 2019;42(1):1-9.

19. Lee CH, Yoon H-J. Medical big data: promise and challenges. *Kidney research and clinical practice*. 2017;36(1):3.

20. Zamboni K, Baker U, Tyagi M, Schellenberg J, Hill Z, Hanson C. How and under what circumstances do quality improvement collaboratives lead to better outcomes? A systematic review. *Implementation Science*. 2020;15(1):27.

21. West M, Dawson J. Employee engagement and NHS performance: King's Fund London; 2012.

22. Lippeveld T. Routine Health Facility and Community Information Systems: Creating an Information Use Culture. *Global Health: Science and Practice*. 2017;5(3):338.

23. CSA. Population and Housing Census Of Ethiopia. 2007.

24. Commission PC. Summary and statistical report of the 2007 population and housing census. Population size by age and sex. 2008.

25. Mnistry Of Health. Information use participants manual, Training Module. In: Policy Plan, and Evaluation Directorate. Addis Ababa. 2018.

26. Obwocha W, Ayodo G, Nyangura A, Thomas O. Utilization of Healthcare Information Among Healthcare Workers in Gucha Subcounty, Kisii County, Kenya. *J Health Edu Res Dev*. 2016;4(192):2.

27. Vaughn VM, Saint S, Krein SL, Forman JH, Meddings J, Ameling J, et al. Characteristics of healthcare organisations struggling to improve quality: results from a systematic review of qualitative studies. *BMJ Quality & Safety*. 2019;28(1):74.

28. Pine KH, Bossen C, Chen Y, Ellingsen G, Grisot M, Mazmanian M, et al., editors. Data work in healthcare: challenges for patients, clinicians and administrators2018.

29. MOH. Health Data Quality Training Module Participant Manual. In: Policy P, and monitoring and evaluation Directorate editor. Addis Ababa: Federal democratic republic of Ethiopia ministry of health; Noveber 2018.

30. Haftu B, Taye G, Ayele W, Habtamu T, Biruk E. A mixed-methods assessment of routine health information system (RHIS) data quality and factors affecting it, Addis Ababa City administration, Ethiopia, 2020. *Ethiopian Journal of Health Development*. 2021;35(1).

31. Slevin P, Kessie T, Cullen J, Butler MW, Donnelly SC, Caulfield B. Exploring the barriers and facilitators for the use of digital health technologies for the management of COPD: a qualitative study of clinician perceptions. *Qjm*. 2020;113(3):163-72.

32. Muthuri RNDK, Senkubuge F, Hongoro C, editors. Determinants of motivation among healthcare workers in the East African Community between 2009–2019: a systematic review 2020: Multidisciplinary Digital Publishing Institute.

33. Hripcsak G, Bloomrosen M, FlatelyBrennan P, Chute CG, Cimino J, Detmer DE, et al. Health data use, stewardship, and governance: ongoing gaps and challenges: a report from AMIA's 2012 Health Policy Meeting. *Journal of the American Medical Informatics Association*. 2014;21(2):204-11.

34. Lagarde M, Huicho L, Papanicolas I. Motivating provision of high quality care: it is not all about the money. *BMJ*. 2019;366.

35. Weldegebriel Z, Ejigu Y, Weldegebreal F, Woldie M. Motivation of health workers and associated factors in public hospitals of West Amhara, Northwest Ethiopia. *Patient Prefer Adherence*. 2016;10:159-69.

Experience, Challenges, and Facilitators of Health Data Use among Performance Monitoring Teams (PMT) of Health Facilities in Eastern Ethiopia: A Qualitative Study.

Admas Abera[1]*, Abebe Tolera[1], Biruk Shalmeno[1], Adisu Birhanu Weldesenbet[1], Assefa Tola[1], Tilahun Shiferaw[2], Alemayehu Girma[3], Rania Mohammed[4] and Yadeta Dessie[1]

[1]School of Public Health, College of Health and Medical Sciences, Haramaya University, Harar, Ethiopia.

[2]Department of Information Sciences, College of Computing and Informatics, Haramaya University, Haramaya, Ethiopia.

[3]Policy and Plan Directorate, Dire Dawa Administration Health Bureau, Dire Dawa, Ethiopia.

[4]Policy and Plan Directorate, Harari Regional Health Bureau, Harar, Ethiopia.

***Corresponding author:**

Email: admasabera10@gmail.com

Abstract

Background: Using Rroutine health data isplays a crucial role in decision-makings andtowards improved health outcomes. Despite the significant investments in improving Ethiopia's Performance Monitoring Team (PMT), there is limited evidence on the level of involvement, implementation strategies, andas well as the facilitators and barriers to data utilization by these teams in responding to present and emerging health challenges. Therefore, tThis study, therefore, is aimed toat exploreing the PMT experiences, facilitators, and barriers to information used in healthcare facilities in Eastern Ethiopia.

Method: This study employed a phenomenological study design. Using the Consolidated Framework for Implementation Research (CFIR) framework was adapted to identify the most relevant constructs, with the aiming to of describeing the data use approaches at six facilities in Dire Dawa and Harari regions in July, 2021. Key informant interviews werewas conducted among 18 purposively selected expertskey informants using a semi-structured interview guide. Thematic coding analysis was applied by using a partially deductive approach, informed by findings from previous studies, and an inductive coding technique with the creation of new emerging themes as they emerged from the transcripts. Data were analyzed thematically using ATLAS.ti analyzed data thematically.

Results: Study participants feltresponded that the primarymain function of PMT wasis improving service delivery. This study also revealed that data quality, performance, service quality, and improvement strategies were among the major focus areas of the PMT. The were issues with the dData utilizeduse by the PMT includingwas affected by poor quality routine data quality, absencelack of accountability, and lack of recognition for outstandinggood personnelperformers. In addition, tThe engagement of PMT members on multiple committees had a negatively impacted on data use leadingthat led to inadequate follow-up of PMT activities, weariness, and insufficient timelack of time to complete responsibilities.

Conclusion: Performance monitoring teams in the health facilities were established and functioning according to the national standard. However, bBarriers to operativeeffective data use included PMT engagement of PMT with multiple committees, poor data quality of collected data, lack of accountability, and poor documentation practices. Addressing the potential barriers by

leveraging the PMT and existing structures have the potential to improve data use and health service performance.

Key words: performance monitoring team, data use, barriers, facilitators, health facilities, Ethiopia.

Introduction

Health systems are complex and continually changing to adapt to~~ changing~~ political, economic, social, technological, and epidemiological realities within constrained resources, particularly in ~~L~~low- and m~~M~~iddle-i~~I~~ncome c~~C~~ountries (LMICs) [1-3]. Health facilities must~~need to~~ cope with these changing realities through organized management and leadership, which require~~ use of~~ reliable data for ~~the ~~developing~~ment of~~ a comprehensive policy package for health sector reforms,~~;~~ improving planning, and continuous monitoring [2, 4].

There has been an increased need for~~ strengthening~~ health service performance strengthening to manage population needs through effective leadership [5-7], and an improved health facility data use culture [8-10]. The increasing demand ~~for ~~and capacity to use data appear~~s~~ more critical~~important~~ than the expanding~~augmenting~~ supply of evidence [3,13] for improved access~~ to~~ and quality of care [4,14]. Collection, processing, transforming, communicating, and using~~using~~ service delivery reports and administrative records are crucial~~play a crucial role~~ in decision-making~~s~~ for~~towards~~ improved health outcomes [15, 16].

Data use is the process through which decision~~s~~-makers and stakeholders explicitly evaluate information in one or more steps~~ of the process~~ of policymaking, program planning,~~ and~~ management, or service provision [6]. In the Ethiopian context, the PMT is a ~~team of~~ multidisciplinary health workforce team ~~that is ~~primarily responsible for data use [11, 12]. By e~~E~~ffectively using~~e of~~ the data, ~~can enable ~~targeted ~~improvements in ~~health service delivery improvements better~~to~~ meet the population's needs [17].

However, in many developing countries, the~~ strength and~~ quality of the evidence needed to generate~~get~~ valid information to make decisions about health programs could be better or more~~is either weak or insufficient in many developing countries~~ [18-20], mainly due to an ineffective data use culture [20]. The major bottlenecks for information use include i~~I~~nadequate infrastructure, leadership turnover, dysfunctional external relations [26], deficient~~poor production of~~

data~~information~~ collection, and limited resources [15]~~ were identified as major bottlenecks for information use~~. In addition, a~~to~~ shortage of skilled workforce, an imbalance~~s~~ in skills~~ mix~~, geographic~~al~~ misdistribution, difficulty in inter-professional collaboration, inefficient use of resources, and burnout were found to affect the health service quality [21].

To date~~So far~~, most~~substantial~~ efforts to strengthen the health information systems~~ mainly~~ have primarily focused on digitization, improving data quality and analysis, and identifying problems;~~,~~ however,~~but~~ the ultimate goal is utilizing~~using~~ information to problem solve~~ problems~~, which requires time to build~~ing~~ an information use culture~~ over time~~ [22]. Therefore, engagement (including involvement, commitment, effort or observable behaviour, a positive effect, or some combination of these) of healthcare leaders and managers is pivotal if we are to improve the h~~H~~ealth i~~I~~nformation s~~S~~ystem (HIS) and hence, the health service delivery [4, 13].

The engagement level and capacities~~ needed by~~ of health managers and performance monitoring teams to respond to current and emerging issues still need to be better~~are not yet well~~ understood [21]. Without radical structural and systemic changes, the existing governance structures and management systems will continue~~ to~~ to fail to address the~~ existing~~ gaps in health service delivery [2]. Furthermore, despite significant~~large~~ investments in performance monitoring teams~~ improvement~~ in Ethiopia, a joint~~common~~ performance renewal effort must be improved~~is lacking~~. Finally, t~~T~~here is a paucity of studies addressing the~~about~~ facilitators, and challenges~~ that~~ influencing~~e the use of~~ health data usage to improve primary health care delivery in LMICs. Therefore, t~~T~~his study~~, therefore, is~~ aimed to~~at~~ identify~~identifying~~ the PMT experiences~~ of the PMT~~, facilitators and barriers to information use~~d process~~ in the healthcare facilities in the Harari region and Dire Dawa city administration~~,~~ in Eastern Ethiopia.

Methods and Materials

Study Setting

The study was conducted in selected~~ public health facilities of~~ Dire Dawa and Harari public health facilities~~regions~~. Dire Dawa is l~~L~~ocated 515 kilometers from Addis Ababa and~~, Dire Dawa~~ has an estimated population of 341,834, with 68.23% ~~of them~~ living in urban areas [23]. Data from the~~on~~ public health facilities indicate~~ that, there are about~~ 15 health centers (8 urban and seven~~7~~ rural), two hospitals, and 32 health posts under the city administration. Harar is the~~a~~ capital of Harari,

one of the regional states of Ethiopia, ~~and is~~ located ~~about~~ 517 Km east of Addis Ababa and 48 km south of Dire Dawa. According to the 2007 census, the region has a total population of 183,415 people [23], and more than half ~~of its people~~ (54.18%) reside in urban areas [24]. The region has~~re are~~ three~~3~~ government hospitals, one~~1~~ university teaching hospital, two~~2~~ private hospitals, nine~~9~~ health centers, and 24 health posts ~~in the region~~.

Study Design and Participants

This study used a phenomenological study design. The Consolidated Framework for Implementation Research (CFIR) ~~framework~~ was used to identify the most relevant constructs to~~with the aim of~~ describe~~ing~~ data use approaches. The CFIR framework is an evidence-based framework ~~drawing~~ from multiple disciplines ~~that~~ providing~~es~~ a comprehensive arrangement of paradigms that influence~~ing~~ complex implementations. The framework has five significant~~major~~ domains (i~~I~~nner setting, o~~O~~uter setting, i~~I~~ntervention characteristics, i~~I~~ndividuals involved, and i~~I~~mplementation process) with associated components~~, all of which were~~ used in this study.

Two hospitals and four health centers were randomly selected from a list of facilities in the two regions. A purposive sampling technique was employed to select the key informants. Eighteen interviews were conducted until data saturation was achieved~~is reached~~. The key informants were PMT members from~~of~~ the health facilities, including hospital medical directors ~~of hospitals~~, heads of health centers, heads of departments ~~in the facilities~~ (outpatient, inpatient, emergency, maternal and child health, pharmacy, laboratory, nursing, human resource, and finance team leads), and the Health Management Information System (HMIS) officers.

Data Collection

We conducted ~~a~~ key informant interviews using a semi-structured interview guide. The tool was comprised of questions related to the respondents' socio-demographic characteristics ~~of the respondents~~, the process of the PMT establishment and implementation strategy process, data use processes and practices, barriers to data use, motivating factors for data use, and recommended mechanisms to improve the data use practice.

Data Quality Control

Data were collected between July 05, 2021, and July 24, 2021, by interviewers fluent in local languages (Amharic and Afan Oromo) and English, with graduate-~~-~~level education and experience in qualitative data collection. All~~ members of the~~ data collection team members underwent~~undergone~~ a ~~one day~~one-day training workshop to~~ have a common~~ understand~~ing on~~ the interview guide and study objectives~~ of the study~~. The data collection activities were supervised ~~on ~~daily~~ basis~~ by the study investigators. All interviews were conducted~~ in~~ privately~~ place suitable for the respondent~~ and~~ the interviews~~ lasted~~ about~~ 20-60 minutes.

Data Processing and Analysis

The interviews were transcribed verbatim, and~~ field~~ notes were taken~~expanded while~~ in~~on~~ the field. The interviews were translated into English and~~ again~~ translated back to the original languages to confirm the accuracy~~ of the meanings~~. A codebook was developed based on the initial review of the transcripts, and transcripts were systematically coded using ATLAS.ti software. Double coding was initially used, with~~ coding~~ disagreements resolved by discussion; ~~and ~~updates to the code definitions were made when~~re~~ needed. Double coding continued until no new disagreements were identified. Subsequently, summaries for each transcript were written under each code using a matrix. Thematic data analysis~~ was used to~~ described and compared general statements as relationships,~~ and~~ themes, and sub-themes emerged~~present on~~ in the data. Accordingly, findings were categorized into five themes. Under each theme, sub-themes were defined with~~and~~ verbatim quotes~~ that~~ representing~~ed~~ opinions~~ were applied to~~ to substantiate the results. ~~Rigor was enhanced through regular discussions between ~~R~~r~~esearchers~~ who~~ read all interview transcripts, counter checked the transcripts, coded the data, and agreed on the emerging themes and sub-themes. Additionally, research rigor was enhanced through regular discussions between researchers.

Key Themes and Sub-themes

In this study, five key themes and~~ about~~ 16 sub-themes~~ have~~ emerged. The main themes and sub-themes~~ emerged from the study~~ are presented in Table 1.

Table 1: Categories of Key themes and sub-themes emerged.

**Themes**	**Sub-themes**
Membership and roles of PMT members at the health facilities	• *Roles and responsibilities of PMT members*<br>• *The current PMT status and experiences*
Relevance of PMT strategies~~y~~ and its implementation	• *Perceived benefits of PMT*<br>• *The modality of PMT meetings*<br>• *Adequacy and convenience of time of PMT meeting*<br>• *The focuses of PMT meetings*
Barriers to data use in the facility	• *Organizational barriers*<br>• *Healthcare workers' work ethic and behavioral barriers*<br>• *COVID-19 effect on data use*

Motivational mechanisms and demotivating factors in enhancing data use practices in place for PMT members	• *Capacity building practices for PMT members*<br>• *Recognition and non-monetary incentives*<br>• *Demotivating factors*
Recommended mechanisms to improve the data use practices	• *Performance-based recognition*<br>• *Continuous supply of resources, and improving HIS infrastructures*<br>• *Strengthening the overall HIS*<br>• *Enhancing the quality of routine data*

Ethical Considerations

~~The ~~E~~e~~thical clearance was obtained from Haramaya University, College of Health and Medical Sciences, Institutional Health Research Ethics Review Committee (IHRERC) ~~with ~~(reference number IHRERC/196/2020). In addition, p~~P~~ermission was sought from ~~all concerned offices, including ~~Dire Dawa Administration and the Harari R~~r~~egional H~~h~~ealth B~~b~~ureaus, and the studied health facilities. Before data collection, informed written consent was obtained from the study participants. Furthermore, participants were~~has been~~ assured of the confidentiality of the information and their right to withdraw from the study at any time during the study. ~~In ~~T~~t~~his paper~~,~~ omitted personal identifiers~~ has been omitted~~ to maintain confidentiality, while neutral identifiers and~~ age of the~~ participants' age were mentioned in direct quotes.

Results

Socio-demographic characteristics

A total of 18 in-depth interviews were conducted in two hHospitals and four health centers to explore the PMT experience, barriers, and facilitators of data use. Of these participants, eight8 were females, and three3 were health facility heads, with aand they have median age (IQR) of 31 (7) years. (Table 2)

Table 2: Socio-demographic characteristics of key informants at the health facilities, Eastern Ethiopia 2021.

Participant ID	Sex	Age in years	Educational status	Work experience in years	Facility type
KI1	Female	34	BSc	11	Health center
KI2	Female	25	Diploma	2	Health center
KI3	Male	27	BSc	9	Health center
KI4	Female	38	BSc	20	Health center
KI5	Male	26	BSc	7	Health center
KI6	Male	34	BSc	13	Health center
KI7	Male	28	Master’s	3	Health center
KI8	Female	25	BSc	1	Health center
KI9	Female	29	BSc	6	Referral Hospital
KI10	Male	28	BSc	32	Referral Hospital
KI11	Male	32	BSc	13	Health center
KI12	Male	30	BSc	7	Health center
KI13	Female	34	BSc	8	Health center
KI14	Female	39	BSc	12	Health center
KI15	Male	42	BSc	19	General Hospital
KI16	Male	35	BSc	3	General Hospital
KI17	Female	35	BSc	12	General Hospital
KI18	Female	30	BSc	10	General Hospital

1. Membership and Roles of PMT at the health facilities

Participants reported that health facilities use specific~~certain~~ criteria to select the members of the PMT,~~.~~ including ~~The main criteria reported to establish the PMT were being the~~ the head of the quality department, HMIS unit team leader, department head, management team, and staff with good performance.

> A respondent stated, "…*since the performance of every department is reviewed in the meeting, the main criteria for PMT membership is being a department head. The heads of management unit, HMIS unit, and quality department are also included. I became a member because I have served as a vice matron for the last one and half years*." (KI9, 29 years old)

> Another key informant pointed out;

> "…*any ~~member~~ health care provider ~~of~~ in ~~the~~ a case team can be a member of the PMT. ~~It is not necessarily be the case team leader.~~ That means there will be one representative from each case team in PMT ~~in~~ irrespective of his/her status.*" (KI15, 42 years old)

Roles and responsibilities of PMT members include us~~eing~~e of health data to~~in~~ improv~~eing~~e the health service performance. The specific roles include preparing action plans, preparing~~putting~~ directions to improve ~~the~~ service delivery, comparing~~e~~ performance overtime, monitoring monthly performance, providing~~e~~ feedback to various units, building the capacity of health workers, and preparing reports. The present study also found that the PMT members were aware of their responsibilities.

> One of the participants stated that "…*each member of the PMT is expected to prepare and submit their case team or department reports to the head of the health facility on time. All PMT members will discuss on the submitted report during the monthly PMT meeting in which the reports are crosschecked for its consistency and quality. When the achievements are lower than expected, the reasons for underachievement are sorted out and future directions are put to improve the performance*." (KI4, 38 years old)

The participants' opinions on ~~the~~ aligning~~ment of~~ the PMT's roles and responsibilities at the facility with their activities were mixed. While the majority of respondents stated that PMT tasks and responsibilities are aligned~~totally matched~~ with their actual activities, some stated they

do not ~~that they are not completely aligned~~ match with their actual activities. There is some overlapping of duties and roles, according to these participants.

> *"As a case team leader, my responsibility is ~~identify~~identifying the challenges in my activities but sometimes the roles and activities rendered to us are not related to service performance and members get frustrated due to that"* (KI16, 35 years old)

2. Relevance of PMT strategy and its implementation

The perceived benefits of PMT for the facility include improving the service delivery and customer satisfaction. Additionally, t~~T~~his team is ~~also~~ essential~~vital~~ for sharing experiences and skills among the departments and case teams.

> *"… the members of PMT are from different departments; there is knowledge sharing on different topics including preparation of reports and how to improve ~~service~~ performance."* (KI11, 32 years old)

Planning, identifying gaps through root cause analysis, and intervening on the identified problems, and monitoring and evaluating specific activities.

> The key informants further pointed out, *"…we select poorly performed activities and the responsible departments will design an action plan for the identified problems by conducting a root cause analysis. Then, the department will be directed to monitor the implementation of the action plan and they are expected to bring the progress in the subsequent meetings."* (KI17, 35 years old)

The modality of PMT meetings. Health facilities have a monthly meeting plan in their health facilities, which is prepared in advance i~~o~~n the annual plan. Respondents pointed out that the monthly meetings were conducted immediately after each unit submitted their monthly report.

> *"Our PMT meeting is conducted on monthly basis. Immediately after the submission of the monthly report (usually at 21st day of the month), we hold our PMT meeting at 23rd or 24th day of the month. Substitution date will be posted on our telegram page if a meeting is rescheduled."* (KI12, 30 years old)

Adequacy and convenience of timinge of PMT meeting. On the other hand, it was found~~indicated~~ that the time allocated for PMT meetings was inadequate and inconvenient. All participants unanimously agreed that there should be enough time~~ should be~~ assigned and a convenient time~~ should be~~ allocated for the PMT meetings.

> *"The meeting time is not enough because every participant usually come to the meeting by interrupting their work. Usually, the meeting date and time is decided by the head."* (KI16, 35 years old)

Participants discussed how holding the meeting on weekends could reduce absenteeism and unnecessary interruptions.

> "…*previously, we used to hold the PMT meeting during working hours and hence the duration of the meetings was not adequate. This was due to high work-load of the PMT members. However, currently we are conducting our PMT meeting on Saturdays. Since it is not a working day, PMT members are paid some pocket money for attending the meeting on over the weekend."* (KI8, 25 years old)

The focus~~es~~ of PMT meetings reportedly included data quality, performance activities (monthly, quarterly, or annual performance), service quality, and~~ the~~ service improvement strategies. According to participants, the first agenda of the PMT meeting was~~is~~ evaluating the previous report, and then comparing it with the current performance. Next~~Afterward~~, the team develops an action plan based on the identified gaps. However, in some health facilities,~~y~~ the previous performance is not conducted at all.

> "*In our monthly PMT meeting, the first thing is presenting the monthly performance report for each unit. Based on the report, gaps and challenges are identified, before action plans are drafted on the identified gaps*". (KI9, 29 years old)

The PMTs mainly~~mostly~~ utilize routine data for evaluation and monitoring of service delivery programs. Therefore, t~~T~~here needed to be a~~was no~~ culture of using health related data from other sources, such as surveys, assessments, or research findings.

“*In our health center, the main target of PMT is the routine data. The data accuracy and completeness are checked. Service improvement strategies are also discussed.*” (KI5, 26 years old)

The respondent further stated *“data collected in our health facility is used for making decisions. I have not seen other data sources from researches or surveys being utilized so far.”* (KI5, 26 years old)

3. Major barriers to data use in the facility

The organizational barriers to data use at the facility level were poor data quality, being a member of multiple~~many~~ committees and high patient flow~~)~~, human resource related issues, input related issues, inadequate budget allocation, and~~as well as~~ lack of performance-~~-~~based incentives.

“…PMT members have their own duties in addition to the PMT activities which makes it difficult to collect quality data due to competing priorities. If someone is engaged with overlapping duties, the desired outcome may not be achieved”. (KI3, 27 years old)

Participants reported that they were in at least two other committees in their health facility. This resulted in~~in turn caused:~~ poor follow-~~-~~up of the designated PMT activities and time constraint with~~ set out by the PMT; being overburdened by~~ committee meetings and assignments. According to the participants, ~~being involved in~~ multiple committees~~ can~~ negatively affected the effectiveness of the PMT activities.

“*…being the member of several committees affects the PMT activities. The aim of including a person in a given team or committee is to perform the task effectively and efficiently in order to bring the intended changes. However, the focus of the individual is usually divided to different committees. In most cases the meeting times is overlapping between different committees”* (KI2, 25 years old)

It was further pointed out:

“Participation in multiple committees decreases the effectiveness of the PMT. Not only for the PMT, but even the other committees are also adversely affected. I suggest ~~that don't~~ to not ~~put~~ assign ~~the~~ case team leaders on more than one committee.” (KI14, 39 years old)

The~~ quality of the~~ routine data quality collected at the health facilities was~~has been~~ reported as a barrier for effective data use.

> *"There is a gap in data quality including gaps with data ~~in~~consistency and ~~in~~completeness. Healthcare providers perform their daily activities~~y~~ but they do not document it on the register regularly. The data from HMIS may contradict from your observation every day. Hence it is difficult to use our data for decision making due to its poor quality."* (KI2, 25 years old)

> Another participant further pointed out *"A timely, reliable and high-quality data should be generated in order to use the data for decision making. Monitoring and supporting the staffs is also critical to obtain high quality data."* (KI17, 35 years old)

HIS i~~I~~nput related factors, including a shortage of patient registrations was cited as another~~the other reason mentioned as~~ barrier for data use.

> *"We have an old register called daily register for documentation of our data. This register was supplied by a Non-Governmental Organization. However, this supply of registers has stopped and currently, we have shortage of the daily registers which affected our documentation."* (KI13, 34 years old)

An additional barrier~~Budget constraint~~ for effective data use at the health facilities was budget constraints, which was felt to~~also an~~ influence documentation in the facilities.

> *"We use the budget~~ which~~ given for the health center, but there is no budget allocated specifically for HMIS."* (KI6, 34 years old)

Healthcare providers~~workers~~' work ethic and behavioural barriers were another~~the other~~ sub-theme which emerged. Poor commitment and lack of accountability from healthcare providers and PMT members were the ~~most commonly reported~~most reported challenges. One respondent explained the commitment and competing priorities of PMT members as

> *"…sometimes we reviewed the same problem repeatedly without a solution. We start to get fed up and start to wonder when it will be solved. Sometimes, we skip the meeting intentionally for this reason.* (KI9, 29 years old)

Another respondent pointed out that:

> *"…health workers that reported incomplete or false data are not given any administrative ~~punishments~~ reprimands for their wrong doing.*" (KI18, 30 years old)

Furthermore, a major challenge is a lack of understanding of the data value by the healthcare workers ~~was indicated as the major challenge faced~~ when using data for action.

> *"Sometimes the PMT members' attitude towards data is a challenge by itself. The notion that a ~~high quality~~high-quality data can change the hospital is not understood equally among the staffs."* (KI9, 29 years old)

According to the participants, most of the health professionals have a poor understanding and ~~poor~~ attitude regarding~~on~~ the importance of data in improving the quality of health services. Additionally, according to the participants there are gaps in s~~S~~kills ~~gaps~~ in analyzing data using electronic computing software, such as District Health Information System version 2 (DHIS2) ~~were also mentioned by the participants~~.

> *"Disparity in understanding the importance of ~~the~~ data among our staffs is the other problem. While some people have better understanding towards data, others do not see the ~~importance~~ value of it."* (KI9, 29 years old)

One~~The~~ respondent further noted:~~;~~

> *"There is a problem on registering the data and handling of a registers. Even after the data is entered into the DHIS2, there is skill gap in analyzing it."* (KI10, 32 years old)

Poor tracking of problems, and a lack of monitoring of action plans is frequently observed according to the key informants.

> *"The main problem is that we don't strictly follow the action plans designed in the previous PMT meetings."* (KI2, 25 Years old)

Absenteeism and interruption due to competing priorities were also reported during PMT meetings.

"As I said earlier, even if it is a monthly meeting, the date and time should be notified ahead of time. Sudden meetings will affect my routine activities and as a result some members of the PMT tend to be absent and some interrupt the meeting to go back to their urgent activities." (KI13, 34 years old)

Poor documentation p~~P~~ractices w~~ere~~as another~~the other~~ challenge reported by PMT members. First, in order to create a~~It was indicated that in order to~~ change in the data use culture, the first priority should be changing the attitude of the health care workers before other interventions are introduced.

"*Health professionals should believe documenting data primarily benefits patients, themselves and their health facility. Therefore, working on the attitude change towards documentation should be given more emphasis."* (KI11, 32 years old)

COVID-19 effect on data use: The COVID-19 pandemic had direct~~ly~~ and indirect~~ly~~ effects~~affected~~ on data use practices at the health facilities. An informant explained the effect of COVID-19 pandemic on their day-to-day activity as

"*Due to the occurrence of COVID -19 outbreak~~s~~, the nearby hospital was entirely devoted to provide health care service for COVID-19 cases. Hence, all patients in the catchment area of that hospital came to our health center. These conditions increased the work-load and compromised our routine activities*." (KI14, 39 years old)

Another key -informant stated "*…due to COVID-19, the head of HMIS has been in quarantine for the last two months which negatively affected the PMT activities."* (KI3, 27 years old)

4. Motivation mechanism in place for PMT members at the facilities

This study identified several motivation mechanisms in place though not across all facilities and~~as well as~~ not conducted regularly~~in a regular manner~~. The main motivation strategies reported were capacity building and performance-based recognition.

Capacity building practices for PMT members: According to participants, the primary~~main~~ capacity building practices included in-service trainings, recognition for good performance, and ~~linked to~~ professional development~~ of the health workers~~.

> *"Capacity building on data quality and project development for the members of PMT has been a very good incentive since we never had such experience before."* (KI7, 28 years old)

Recognition and Non-monetary incentive mechanisms to~~that can~~ motivate the PMT members in the facility were reported. Participants indicated that these motivational mechanisms were~~become~~ effective when~~if they are~~ directed towards improving the data use culture of the facilitiesies~~ies~~.

> A participant pointed out that "*p~~P~~erformance-based recognition and motivation of staffs can improve the data quality and subsequently the data use in the facility."* (KI6, 34 years old)

However, in some health facilities there were not any motivation measures specific to PMT members.

> *"There are no capacity buildings, recognition mechanisms, and career enhancing educational ~~chance~~ opportunities to motivate PMT members."* (KI4, 38 years old)

Other participants reported that some sort of recognition~~s~~ and motivation mechanisms were implemented in their facilities.

> *"They awarded me this watch for my good performance for properly ensuring the quality of data collected in my health facility."* (KI7, 30 years old)

Others believed that incentives may not necessarily motivate staff~~s~~ to use the data or improve service performance.

> *"It is hard to say that the presence of incentives only positively affects data use. Providing incentives before attitude change may even adversely affect the data use practice."* (KI11, 32 years old)

Demotivating factors for effective data use practices were cited by the study participants~~. The main demotivating factors~~ including~~e~~ a shortage~~s~~ of resources, inadequate salary, and inadequate follow~~-~~up.

> "*Salary is one of the demotivating factors because it does not fit with the job that we undertake.*" (KI10, 28 years old)

> ***"****There is no follow up mechanism after trainings are provided.*" (KI8, 25 years old)

The managers' leadership skill~~s of the managers~~ was one of the factors affecting data use in health facilities. Health managers' and case-team/departments' poor leadership skills, and lack of value for data were also mentioned as demotivating factors.

> *"The head of the facility lacks decisiveness and leadership qualities on this job. I don't think the problem is a gap in knowledge but rather an attitude problem."* (KI13, 34 years old)

> Another respondent pointed out "*…if the head of the department or the facility do not utilize data properly, other employees will not care about data use. Hence, the head at district, region or at any other level should lead by example to motivate ~~the~~ other staffs."* (KI2, 25 years old)

5. Recommended motivation mechanisms for an improved data use

Performance-based recognition was recommended by all informants~~ in this study~~ as a major motivation mechanism to improve data use.

> *"There are those who register and report on time among the health extension workers.~~,~~ T~~t~~he others will be motivated to do the same if you ~~give~~ provide recognition or incentives ~~for those health workers~~. The incentive can even be in the form of a certificate."* (KI15, 42 years old)

> Another participant stated, *"s~~S~~ince the PMT members are the backbone of ~~the~~ health facilities, designing motivating mechanisms is important."* (KI9, 29 years old)

Continuous supply of resources, improving the infrastructures, and strengthening the HIS.

"*Ensuring continuous supply of necessary materials~~,~~ and recognizing best performers should be introduced.*" (KI9, 29 years old)

Enhancing the quality of routine data collected was also unanimously believed to enhance data use practice.

"In order to use ~~the~~ data for a decision, first I have to receive the data properly. A timely, reliable and complete data should be collected." (KI1, 34 years old)

Discussion

~~Adapting the Consolidated Framework for Implementation Research (CFIR),~~ T~~t~~his study explored experiences, facilitators and barriers of the ~~of~~ PMT, using the adapted Consolidated Framework for Implementation Research (CFIR), to generate~~in the use~~ information for decision-making at points of healthcare delivery. ~~.~~ The findings revealed that the PMT in most facilities were established according to the standard criteria ~~for establishment of PMT~~ set by the Ministry of Health (MOH) of Ethiopia~~.~~ [12]. In line with previous studies, our study indicated that the primary responsibility of the PMT was to improve data quality and ~~use of information to~~ regularly monitor progress and improve health service performance [11, 12].

Although some irregularities were reported, the monthly meetings were conducted ~~immediately~~ right after each unit submitted their monthly report and before submitting their report to the next level to monitor progress and improve performance. The MOH guideline indicates that the meeting dates, venue and its members should be officially communicated in advance and the meeting should be conducted at least a day ahead of submission of the monthly report to the next level [11, 12, 25].

The present study found ~~that~~ the ~~major focus area of~~ PMT meetings focused on ~~were~~ data quality, performance improvement, and evaluation of previous action plans. Previous s~~S~~tudies show PMT meetings should start with~~on this indicate,~~ follow-up activities for gaps highlighted from~~in~~ the

previous PMT meeting as the~~should always be the~~ first item on the agenda, followed by an assessment of the progress on those gaps [12, 25]. Meetings and collected data have no value~~ in themselves~~ unless action items from meetings are implemented and data are analyzed into meaningful actions [26].

Health facilities should design strategies to minimize the number of committees and integrate similar committees to improve their service provision. Most PMT members in our study were usually involved in at least two other committees in their health facility. This creates~~has its own share of~~ poor follow up of the activities set out by the PMT~~;~~ with the committee members being overburdened by~~ committee~~ meetings and assignments,~~;~~ creating fatigue and a shortage of time to accomplish PMT assignments.

Evidence indicates data triangulation through~~ the use of~~ other sources, such as original research, community feedback, expert opinions, vital registration, censuses, and routine HMIS data can yield better results [12,25,26]. Although there were practice of use of routine internal data, information use from external sources was limited.

Previous studies indicated that an organizational context that supports data collection, availability, and use, the technical aspects of data processes and tools, and the behavior of individuals who produce and/or use data are the main elements of health information use [25, 27]. The major challenges of data use reported in the present study emanate from organizational, behavioral and technical sources including poor data quality, competing priorities, shortage of skilled human power, and lack of performance-based motivation for the health workers.

Healthcare organizations are increasingly required to gather and report data about their performance and respond to the results of consequential quality measurements [28]. Quality data enables healthcare organizations to monitor progress, making decisions for continuous improvement, and provide effective and efficient health services [29]. Our study revealed that although poor quality of data was one of the major challenges for an informed decision making, it has been used for priority setting as well as designing and implementing action plans. This requires attention as decisions taken by healthcare managers might be misleading and fails to address the actual problem. Previous study Addis Ababa indicated that the PMT meetings that were designed for the sole purpose of improving data quality were not effective [30]. Another qualitative study

to explore the facilitators and barriers of digital health technology use also identified data quality as a potential barrier [31]. Lack of available data for several indicators and a lack of validated indicators for important dimensions of quality were identified as major challenges to improve Primary Health Care (PHC) Performance [6].

Behavioral factors provide crucial insight into the way in which health workers, managers and policymakers use information [25]. The attitude, motivation, and value decision makers attach to information play a big role in determining data use [2, 25]. Concurrent with these, poor commitment, skill gap, lack of accountability for failing to accomplish tasks, work overload, poor documentation, and resource mismanagement were the main behavioural level challenges reported in this study.

Engagement with different committees increased PMT workload and compromised the effectiveness of data use. Participation in quality improvement collaborative activities may improve health professionals’ knowledge, problem-solving skills and attitude; teamwork; shared leadership and habits for improvement. A review of literature indicated shifting roles and expectations in the workplace for health care leadership and management as a major challenge for health leadership and workforce [1]. Interaction across quality improvement teams may generate normative pressure and opportunities for capacity building and peer recognition [11].

Health professionals either do not consider recording and reporting data as part of their routine activities or they just give more priority to the clinical provision and lesser attention to data [30]. Although there is a need to make significant investments in workforce development and training [33], tackling of behavioral factors require interventions that go beyond simple trainings and should focus more on initiatives that enhances positive attitude towards data value [25].

The individual determinant of motivation among healthcare workers are altruism, attaining professionalism, educational opportunity, and being more experienced [32]. In the present study, it was indicated that health facilities use capacity buildings, and some form of non-monetary incentives to improve performance, while most informants indicated dissatisfaction with the absence of such motivating mechanisms. Career development opportunities, in-service trainings, and regular recognition for good performance were also reported to be practiced at the health facilities.

Provision of inputs necessary for HIS such as laptops, internet modems; as well as awarding the best performing health facilities were other motivational methods. Since health professionals can be motivated by a range of extrinsic and intrinsic factors, policy makers need to look beyond traditional financial incentives when designing policies to improve health service performance. [34] Studies indicate training opportunities, transformative leaders, and nonmonetary incentives, staff development and promotion, availability of necessary resources, and ease of communication are found to be effective motivation mechanisms for health care workers [32, 35].

Conclusion

This study has generated important insights into PMT establishment, ~~its~~ implementation strategies~~y~~, barriers to~~for~~ data use, and recommendations to enhance data use practice at the~~a~~ point of healthcare service delivery. The study found that most performance monitoring teams in health facilities were established and functioning according to the national standard. Additionally, t~~T~~he study revealed~~also underscored~~ that the~~at~~ barriers to effective data use include the~~engagement of~~ PMT attending~~with~~ multiple committee~~s~~ meetings (increasing workload), poor data quality~~ of data~~, lack of accountability, and poor documentation practices. Recommendations to enhance data use practices include n~~N~~on-monetary incentives and recognition~~s~~ for exemplary health workers~~ were recommended as means of enhancing data use practice~~. Improving routine data~~the~~ quality~~ of routine data~~, integrating various~~different~~ teams into PMT, establishing an accountability framework, and designing documentation methods~~-~~ can all~~have the potential to~~ improve informed decision-~~-~~making. While a comprehensive countrywide study of~~ the~~ PMTs is required, policy makers, stakeholders working in HIS, and health managers should work on improving routine data quality~~,~~ and design motivational strategies, including recognition and non-monetary incentives to improve data use which has the potential to improve health service performance.

Acknowledgements

The authors are thankful to the Ethiopian Ministry of Health~~ of Ethiopia~~, Dire Dawa and Harari R~~r~~egional H~~h~~ealth B~~b~~ureaus, health facilities, data collectors, and study participants.

Reference

1. Figueroa CA, Harrison R, Chauhan A, Meyer L. Priorities and challenges for health leadership and workforce management globally: a rapid review. *BMC Health Services Research*. 2019;19(1):239.

2. Senkubuge F, Modisenyane M, Bishaw T. Strengthening health systems by health sector reforms. *Global health action*. 2014;7(1):23568.

3. Witter S, Anderson I, Annear P, Awosusi A, Bhandari NN, Brikci N, et al. What, why and how do health systems learn from one another? Insights from eight low- and middle-income country case studies. *Health Research Policy and Systems*. 2019;17(1):9.

4. Nordsteien A, Byström K. Transitions in workplace information practices and culture. *Journal of Documentation*. 2018;74(4):827-43.

5. Veillard J, Cowling K, Bitton A, Ratcliffe H, Kimball M, Barkley S, et al. Better measurement for performance improvement in Low-and Middle-Income countries: the primary health care performance Initiative (PHCPI) experience of conceptual framework development and indicator selection. *The Milbank quarterly*. 2017;95(4):836-83.

6. Tantivess S, Walt G. The role of state and non-state actors in the policy process: the contribution of policy networks to the scale-up of antiretroviral therapy in Thailand. *Health policy and planning*. 2008;23(5):328-38.

7. Fryatt R, Bennett S, Soucat A. Health sector governance: should we be investing more? *BMJ global health*. 2017;2(2):e000343.

8. Lippeveld T. Routine health facility and community information systems: creating an information use culture. Global Health: Science and Practice; 2017. p. 338-40.

9. Nordsteien A, Byström K. Transitions in workplace information practices and culture: The influence of newcomers on information use in healthcare. *Journal of Documentation*. 2018.

10. Jylhä V. Information management in health care: a model for connecting information culture and patient safety. 2017.

11. Adane T, Tadesse T, Endazenaw G. Assessment on Utilization of Health Management Information System at Public Health Centers Addis Ababa City Administrative, Ethiopia. *Internet Things Cloud Comput*. 2017;5(1):7-18.

12. MOH. Information Use Training Module. In: PPMED. Addis Ababa; June 2018.

13. Aqil A, Hozumi D, Lippeveld T. Tools for data demand and use in the health sector: Performance of Routine Information Systems Management (PRISM) Tools. *Measure Evaluation*. 2011.

14. Watson DE, Broemeling A-M, Reid RJ, Black C. A results-based logic model for primary health care : laying an evidence-based foundation to guide performance measurement, monitoring and evaluation. 2004.

15. Endriyas M, Alano A, Mekonnen E, Ayele S, Kelaye T, Shiferaw M, et al. Understanding performance data: health management information system data accuracy in Southern Nations Nationalities and People's Region, Ethiopia. *BMC Health Services Research*. 2019;19(1):175.

16. MOH. Guidance on developing key performance indicators and minimum data sets to monitor healthcare quality. 2013.

17. Rendell N, Lokuge K, Rosewell A, Field E. Factors That Influence Data Use to Improve Health Service Delivery in Low- and Middle-Income Countries. *Global Health: Science and Practice*. 2020;8(3):566.

18. Maphumulo WT, Bhengu BR. Challenges of quality improvement in the healthcare of South Africa post-apartheid: A critical review. *Curationis*. 2019;42(1):1-9.

19. Lee CH, Yoon H-J. Medical big data: promise and challenges. *Kidney research and clinical practice*. 2017;36(1):3.

20. Zamboni K, Baker U, Tyagi M, Schellenberg J, Hill Z, Hanson C. How and under what circumstances do quality improvement collaboratives lead to better outcomes? A systematic review. *Implementation Science*. 2020;15(1):27.

21. West M, Dawson J. Employee engagement and NHS performance: King's Fund London; 2012.

22. Lippeveld T. Routine Health Facility and Community Information Systems: Creating an Information Use Culture. *Global Health: Science and Practice*. 2017;5(3):338.

23. CSA. Population and Housing Census Of Ethiopia. 2007.

24. Commission PC. Summary and statistical report of the 2007 population and housing census. Population size by age and sex. 2008.

25. Mnistry Of Health. Information use participants manual, Training Module. In: Policy Plan, and Evaluation Directorate. Addis Ababa. 2018.

26. Obwocha W, Ayodo G, Nyangura A, Thomas O. Utilization of Healthcare Information Among Healthcare Workers in Gucha Subcounty, Kisii County, Kenya. *J Health Edu Res Dev*. 2016;4(192):2.

27. Vaughn VM, Saint S, Krein SL, Forman JH, Meddings J, Ameling J, et al. Characteristics of healthcare organisations struggling to improve quality: results from a systematic review of qualitative studies. *BMJ Quality & Safety*. 2019;28(1):74.

28. Pine KH, Bossen C, Chen Y, Ellingsen G, Grisot M, Mazmanian M, et al., editors. Data work in healthcare: challenges for patients, clinicians and administrators2018.

29. MOH. Health Data Quality Training Module Participant Manual. In: Policy P, and monitoring and evaluation Directorate editor. Addis Ababa: Federal democratic republic of Ethiopia ministry of health; Noveber 2018.

30. Haftu B, Taye G, Ayele W, Habtamu T, Biruk E. A mixed-methods assessment of routine health information system (RHIS) data quality and factors affecting it, Addis Ababa City administration, Ethiopia, 2020. *Ethiopian Journal of Health Development*. 2021;35(1).

31. Slevin P, Kessie T, Cullen J, Butler MW, Donnelly SC, Caulfield B. Exploring the barriers and facilitators for the use of digital health technologies for the management of COPD: a qualitative study of clinician perceptions. *Qjm*. 2020;113(3):163-72.

32. Muthuri RNDK, Senkubuge F, Hongoro C, editors. Determinants of motivation among healthcare workers in the East African Community between 2009–2019: a systematic review 2020: Multidisciplinary Digital Publishing Institute.

33. Hripcsak G, Bloomrosen M, FlatelyBrennan P, Chute CG, Cimino J, Detmer DE, et al. Health data use, stewardship, and governance: ongoing gaps and challenges: a report from AMIA's 2012 Health Policy Meeting. *Journal of the American Medical Informatics Association*. 2014;21(2):204-11.

34. Lagarde M, Huicho L, Papanicolas I. Motivating provision of high quality care: it is not all about the money. *BMJ*. 2019;366.

35. Weldegebriel Z, Ejigu Y, Weldegebreal F, Woldie M. Motivation of health workers and associated factors in public hospitals of West Amhara, Northwest Ethiopia. *Patient Prefer Adherence*. 2016;10:159-69.

		**Experience, Challenges, and Facilitators of Health Data Use among Performance Monitoring Teams (PMT) of Health Facilities in Eastern Ethiopia: A Qualitative Study.**	
		**Reviewer's 2 Comments**	**Authors' Responses**
	Methods and Materials	Study Setting: Back ground information for both Cities is not similar. See disaggregated data by sex and number of health professionals. It should be consistent.	First of all the authors would like to acknowledge the editor and reviewers for insightful comments to make the manuscript better.<br><br>• The study setting has been made consistent by removing less relevant information on sex disaggregation.
		How the result organized after the socio demographic characteristics better follow the logical flow of the topic for easy understanding by readers. Sub titles could be given.<br>1. Experience<br>2. Barriers<br>3. Facilitators	✓ Many thanks for the important insight that the authors were also discussing when writing the paper.<br>• Although it is quite right to present the results consistent with the title, we think rather than chopping down these themes into experience, barriers and facilitators, it would be more informative to classify them into the five themes to reflect the findings. In addition, these themes emerged from the data and there were a lot of negotiations among the investigators to reach to these themes.<br><br>• Overall, the first two themes (**Membership and roles of PMT members** and **Relevance of PMT strategy and its implementation)** represent the experience, and the last 2 themes **(motivation mechanisms and recommended mechanism)** represent facilitators. **Hence, we believe the components have been addressed.**<br>• We would like to ask the reviewer to consider our reason for presenting the results in such manner.

2	Results	Most importantly, the authors mentioned in CFIR in their methodology.<br>But in the result, the frame work (the five themes) has hardly reflected. They might have applied appropriately but that should be shown in the result for its scientific merit. It would have been possible that the three titles along with their sub themes could be summarized in CFIR framework.	• Thank you again for this important comment and raising the issue.<br><br>• Yes in this study, we have used CFIR framework with the five components to describe the data use experience and potential barriers and facilitators. The primary use of the framework is that it considers holistic view of what affects a certain phenomenon with the view of improving and informing implementation of an intervention.<br>• However, in this study we employed the framework to have a comprehensive look of data use approaches considering all the five constructs (inner setting, outer setting, individuals involved, implementation process and intervention characteristics). Although all of the constructs have been utilized in this study, we focused on those issues related to the experience, barriers and facilitators of data use by the PMT. And the authors believed that presenting the data in terms of the experience, barriers and facilitators will inform programs and policy much better than the constructs in the CFIR framework.
4.	Results	I wonder if the investigators might have explored the PMT's experience of conducting root cause analysis for potential intervention	• Thank you again for this important point.<br><br>• Yes, we have explored one of the major role of the PMT i.e. Root cause analysis and we think it is important to include it here. By **“identifying gaps”,** we were referring to the conduct of root cause analysis but it merits to be clearly indicated in the roles of PMT section. We have now included additional sentence and explained it.
5.	Results	What is the experience of the PMT's evidence of having meeting regularly. like having minute and signed by each meeting participant	• Thank you for the comment. However, this study have not explored this as it was assessed quantitatively in a separate paper.

6.	Results	How do the investigators explain poor data in the context of PMT's experience of using routine data, highlighted above, (which is poor in this finding) for setting action plan and improve the performance. Doesn't this lead to poor/wrong action plan and poor/wrong performance?	• This is an important point to note. Numerous researches indicate that quality of routine data is a major problem in the health sector in many part of the country but at the same time participants responded that they use it for priority settings and other interventions as it is the only (major) source of evidence at health facilities. Data quality at health facilities is measured by different dimensions including timeliness and completeness.<br>• Yes, we agree this has its own impact on the performance and action plan being set and this gap has to be explained/discussed.<br>• A discussion point regarding these contradictory concepts have been included in the discussion section.
8.	Results	The investigators attempt to explore the barrier with potential solution is remarkable. However, it would have been good if they could include for the aforementioned barriers too.	• Thank you for your comment on this.<br>• The findings obtained from the study is what we presented here. Yes it would be important to include the possible interventions for the barriers but those mentioned by the key informants have been explained in the present study.
9.	Discussions:	See the above comment on CFIR under the result.	• This has been explained above
15.	General:		

Academic Editor's questions	Author's responses
Make sure that the formatting of the manuscript is according to the journal's requirements. Include required sections following the conclusions statement.	Firstly, we would like to thank the editor for providing the comments to improve our manuscript.<br><br>The required sections following the conclusions have been included. The journal guideline indicates that only references and acknowledgements need to be included after the conclusion. Funding and other statements were included in the submission system.
Use square brackets (i.e., [ ]), rather than ( ) for inside text citation of references. Full stops should come after the in-text citations (i.e., [1,3].)	• These have been corrected throughout the paper
Briefly explain how the CFIR framework has been applied to guide the present study.	• CFIR framework was briefly explained under study design (1st paragraph).
The organization of the methods sections has unclarity. The description of sample size and sampling strategy should be provided prior to data collection procedures. Thus, it is highly recommended if it is restructured as follows:<br><br>• Study setting<br>• Study design and participants: here try to briefly describe the study design, sample size, sampling strategy, and inclusion/exclusion criteria.<br>• Data collection<br>• Data quality control: here briefly explain the strategies used to ensure the quality of data.<br>• Data processing and analysis<br>• Ethical consideration	The methods section has been revised based on these suggestions.

There are grammar and language flaws that requires revisions. For instance: take a look at the statement in page 11 that starts with: 'planning, identifying gaps and intervening****'; page 12: 'the focus of PMT meeting****etc..."	We have critically went over the manuscript for English language. In addition, the paper was sent for a native speaker for further refinement. The revised parts can be seen with the track changes.
The paper has reported a very good results, however the discussion is weaker. More evidences/literatures should be used to support the justifications and the implications of observed results should be briefly presented. Moreover, authors are highly suggested to discuss the limitations of the present study (if any)	•
