# Supplementary material for: Experiences, barriers, and facilitators of health data use among performance monitoring teams (PMT) of health facilities in Eastern Ethiopia: A qualitative study
Source: PLoS One. 2023 May 11;18(5):e0285662. doi: 10.1371/journal.pone.0285662 (PMC10174501; doi:10.1371/journal.pone.0285662)
Supplement: Attachment — Submitted filename: Response to Reviewers.docx [file pone.0285662.s002.docx]

| **Section** | **Reviewer’s comment** | **Authors’ responses** |
| --- | --- | --- |
| **General** |  | The authors are really thankful for the insightful comments and feedback from the reviewer to improve our paper. We have critically looked at all the reviewer’s comments and addressed all of them in detail. The addressed parts are explained below on a point-by-point response |
| **Abstract** | Typo errors suggested | All typos have been corrected which can be seen in the revised manuscript. |
| **Introduction** | Typo errors suggested | All typos have been corrected which can be seen in the revised manuscript. |
| **Methods and Materials** | The sentence is repetition of the last sentence in a paragraph above. You may consider choosing which to keep (page 5, last paragraph) | The repeated sentence has been removed to make the writing brief. |
|  | This covers forward translation. What about the backward translation to confirm accuracy of the translation? (page 6, first paragraph) | Additional sentence indicating the backward translation is included in the revised version. We have conducted the translation but we failed to include it in the initial version. Thank you for pointing this out. |
|  | Can you further indicate on how trustworthiness was ensured (page 6, first paragraph) | How the rigor and trustworthiness of the study was ensured has been explained under data analysis section. |
| **Results** | A fifth theme may also be necessary.  This should be on relevance of PMT strategy and its implementation. Some sub-themes listed under key theme 1 do not necessarily blend well to the theme title | We found this comment useful and a fifth theme has been included as per your suggestion. The authors would like to thank you on this. |
|  | A fifth theme may also be necessary.  This should be on relevance of PMT strategy and its implementation. Some sub-themes listed under key theme 1 do not necessarily blend well to the theme title (page 8, first paragraph) | A fifth theme has been added by classifying the first theme into *membership and roles of PMT at health facilities* and *relevance of PMT strategy and its implementation. Many thanks for the suggestion on this.* |
|  | Given the sample size for the study and generally qualitative studies, this identifier is likely to compromise anonymity. Please consider using other more neutral identifier like participants ID. With other facilities, it happens that there is only one program planning team leader and subsequently one could easily identify who said what. (page 8, second qoute paragraph) | Dear reviewer, I think this is an important issues to note. We agree on this point on ensuring the ethical issues and confidentiality of the participants. To remedy this, we have removed the working position of the key informants and replaced it with neutral identification (KI1, KI2, etc.) which stand for Key Informant 1, 2,…. And age of the respondent in the direct quotes. |
|  | It is not clear why this is in bold letters. By the look, it seems as a sub-theme. However, there was no indication in the data analysis section that there were also such.  Please synchronise | Again, this comment is valid and accepted. To make sure the writing is consistent, we have clearly indicated the themes and sub-themes in Table 2. All the sub-themes are written in bold letters in the revised manuscript to make the writing clearer for readers and the themes are presented as a heading. Thank you for the feedback. |
|  |  |  |
|  | There is no data from participants presented under this section. (under roles and responsibilities of PMT result) (page 9, first paragraph) | The reviewer have raised a number of important feedback on lack of direct quotes from respondents. This comment has been accepted. Throughout the entire paper, we have tried to make significant revisions by adding more direct quotes from key informants. Please look at the revisions made for the whole results. |
|  | I suggest you also indicate under methods for data collection sub-themes that emerged (page 9, fourth paragraph) | All the themes and sub-themes emerged has been presented clearly in Table 1 under the methods section in the revised version. Kindly check table 1. |
|  | Please confirm if: 1. PMT members are paying for attending the meeting on Saturdays, or 2. PMT member are paid and get pocket money for attending meeting over weekend.  As it stands, this sentence of the transcription is confusing. (page 10, fourth paragraph) | This sentences has been revised to make the meaning clearer. The PMT members were paid by the health facility when they attend the meeting on weekends. Please check the revised version under adequacy and convenience of time of PMT meeting sub-theme. |
|  | It becomes more rich when participants quotations are provided to show what they reported above what the author will present as having been reported. In this paragraph, and several others, authors do not show what participants reported yet there are strong findings which one would wish to verify and compare to the presentation. (page 11, 3rd paragraph) | This well accepted. Throughout the entire paper, we have tried to make significant revisions by adding more direct quotes from key informants. Please look at the revisions made for the whole results. Almost all results have been supported by the direct quotes from key informants in the revised version. |
|  | Effect of multiple committees can be better positioned under barriers. Esp under organizational (Page 11, 4^th^ paragraph) | The effect of multiple committee on data use has been moved to organizational barrier. |
|  | It is not clear whether this paragraph discusses or introduces views of participants. If it introduces reported organisational barriers, as it should, please also put direct quotes from participants indicating these barriers. Moving effects to this position may remedy this. (Page 12, 2^nd^ paragraph) | It introduces the reported barriers and as explained above, we have now revised this and added direct quotes in the writings. |
|  | Incentives and recognition results are motivating factors and might not jell with barriers. Please move to motivational mechanisms below (Page 13, 2^nd^ paragraph) | This is accepted and moved motivational mechanisms sub-theme. |
|  | Health workers' work ethics might be more relevant term than health workers behavioural factors (Page 13, 3^rd^ paragraph) | Thank you for the feedback on this. We believe some of the factors initially were under organizational barriers (such as attitude, skill gap and knowledge) move beyond work ethic. However, commitment, poor documentation practice are indeed work ethics issues. To accommodate these concepts, we have revised the sub-theme as “health workers work ethics and behavioural barriers” We hope this can cover all the concepts raised by the key informants. |
|  | This is a key theme  Please reconsider titling/heading of your themes and sub-themes for consistency (Page 16, 1^st^ paragraph) | Accepted and revised. |
| Discussion | A section under findings, which did not reflect on actual data from participants, suggested that participants reported some form of incentives. This is not precise reflection of findings. The same section reflected dissatisfaction about the lack of these mechanisms. Please confirm and create synergy | Dear reviewer, thank you for the insightful comment on this. We have revised this so that it directly reflects the finding of the study. The discussion part is revised to make it consistent with the result. |
|  | This has not transpired under findings. Please discuss your results OR confirm this in your results. (first sentence of the last paragraph on page 20) | This sentence was not the finding of the study rather we were discussing finding from other literature. It was not clear and has been revised in the revised manuscript. In addition, the references that were not in the list were omitted. It was a typo. |
| **Grammatical errors and typos in the entire paper** |  | **We are really grateful for reviewing the manuscript in detail looking for typos and grammatical errors. All of these have been accepted and corrected per your comment in the revised paper.** |
