# Supplementary material for: Experiences, barriers, and facilitators of health data use among performance monitoring teams (PMT) of health facilities in Eastern Ethiopia: A qualitative study
Source: PLoS One. 2023 May 11;18(5):e0285662. doi: 10.1371/journal.pone.0285662 (PMC10174501; doi:10.1371/journal.pone.0285662)
Supplement: Attachment — Submitted filename: Academic Editor Responses.docx [file pone.0285662.s005.docx]

| **Academic Editor’s questions** | **Author’s responses** |
| --- | --- |
| Make sure that the formatting of the manuscript is according to the journal’s requirements. Include required sections following the conclusions statement. | Firstly, we would like to thank the editor for providing the comments to improve our manuscript.  The required sections following the conclusions have been included. The journal guideline indicates that only references and acknowledgements need to be included after the conclusion. Funding and other statements were included in the submission system. |
| Use square brackets (i.e., [   ]), rather than (   ) for inside text citation of references. Full stops should come after the in-text citations (i.e., [1,3]. ) | - These have been corrected throughout the paper |
| Briefly explain how the CFIR framework has been applied to guide the present study. | - CFIR framework was briefly explained under study design (1^st^ paragraph). |
| The organization of the methods sections has unclarity. The description of sample size and sampling strategy should be provided prior to data collection procedures. Thus, it is highly recommended if it is restructured as follows:   - Study setting - Study design and participants: here try to briefly describe the study design, sample size, sampling strategy, and inclusion/exclusion criteria. - Data collection - Data quality control: here briefly explain the strategies used to ensure the quality of data. - Data processing and analysis - Ethical consideration | The methods section has been revised based on these suggestions. |
| There are grammar and language flaws that requires revisions.  For instance: take a look at the statement in page 11 that starts with: 'planning, identifying gaps and intervening****'; page 12: 'the focus of PMT meeting****etc..." | We have critically went over the manuscript for English language. In addition, the paper was sent for a native speaker for further refinement. The revised parts can be seen with the track changes. |
| The paper has reported a very good results, however the discussion is weaker. More evidences/literatures should be used to support the justifications and the implications of observed results should be briefly presented. Moreover, authors are highly suggested to discuss the limitations of the present study (if any) |  |
