# Supplementary material for: Experiences, barriers, and facilitators of health data use among performance monitoring teams (PMT) of health facilities in Eastern Ethiopia: A qualitative study
Source: PLoS One. 2023 May 11;18(5):e0285662. doi: 10.1371/journal.pone.0285662 (PMC10174501; doi:10.1371/journal.pone.0285662)
Supplement: Attachment — Submitted filename: Author Responses_V4.docx [file pone.0285662.s007.docx]

|  |  | **Experience, Challenges, and Facilitators of Health Data Use among Performance Monitoring Teams (PMT) of Health Facilities in Eastern Ethiopia: A Qualitative Study.** | |
| --- | --- | --- | --- |
|  |  | **Reviewer’s 2 Comments** | **Authors’ Responses** |
| 1 | Methodology | 1. Within the manuscript briefly describe the themes and sub-themes related to experience, barriers and facilitators  Sub titles could be given.  1. Experience  2. Barriers  3. Facilitators | - Many thanks again for the important comments to improve the MS. - These have been revised and the themes have been re-organized per the reviewer’s suggestion. (3 major themes and the other emerging themes as sub-themes). Please see the revised manuscript. |
| **2** | Results | In the methods, provide more information regarding how CFIR framework guided the present research and at what stage of the research. | - Thank you again for this important comment. - We have tried to provide I specifically how the CFIR framework was used for this particular study. We have also indicated which of the 5 domains were applied for our study. Thanks for the perspective in this regard. |
